# Development of guidelines for the surveillance of invasive mosquitoes in Europe

**DOI:** 10.1186/1756-3305-6-209

**Published:** 2013-07-18

**Authors:** Francis Schaffner, Romeo Bellini, Dušan Petrić, Ernst-Jan Scholte, Hervé Zeller, Laurence Marrama Rakotoarivony

**Affiliations:** 1 Avia-GIS, Risschotlei 33, B-2980 Zoersel, Belgium; 2 Centro Agricoltura Ambiente “G. Nicoli”, Via Argini Nord 3351, 40014 Crevalcore, Italy; 3 University of Novi Sad, Faculty of Agriculture, Laboratory for Medical Entomology, Trg D. Obradovića 8, 21000 Novi Sad, Serbia; 4 National Centre for Monitoring of Vectors, Dutch Food and Consumer Product Safety Authority (NVWA), Geertjesweg 15, P.O. Box 9102, 6700, HC Wageningen, The Netherlands; 5 ECDC, European Centre for Disease Prevention and Control, Tomtebodavägen 11A, 17183 Stockholm, Sweden

**Keywords:** Invasive mosquitoes, Aedes, Surveillance, Monitoring, Vector, Dengue, Chikungunya, Europe, Guidelines

## Abstract

**Background:**

The recent notifications of autochthonous cases of dengue and chikungunya in Europe prove that the region is vulnerable to these diseases in areas where known mosquito vectors (*Aedes albopictus* and *Aedes aegypti*) are present. Strengthening surveillance of these species as well as other invasive container-breeding aedine mosquito species such as *Aedes atropalpus, Aedes japonicus*, *Aedes koreicus* and *Aedes triseriatus* is therefore required. In order to support and harmonize surveillance activities in Europe, the European Centre for Disease Prevention and Control (ECDC) launched the production of ‘Guidelines for the surveillance of invasive mosquitoes in Europe’. This article describes these guidelines in the context of the key issues surrounding invasive mosquitoes surveillance in Europe.

**Methods:**

Based on an open call for tender, ECDC granted a pan-European expert team to write the guidelines draft. It content is founded on published and grey literature, contractor’s expert knowledge, as well as appropriate field missions. Entomologists, public health experts and end users from 17 EU/EEA and neighbouring countries contributed to a reviewing and validation process. The final version of the guidelines was edited by ECDC (Additional file 1).

**Results:**

The guidelines describe all procedures to be applied for the surveillance of invasive mosquito species. The first part addresses strategic issues and options to be taken by the stakeholders for the decision-making process, according to the aim and scope of surveillance, its organisation and management. As the strategy to be developed needs to be adapted to the local situation, three likely scenarios are proposed. The second part addresses all operational issues and suggests options for the activities to be implemented, *i.e.* key procedures for field surveillance of invasive mosquito species, methods of identification of these mosquitoes, key and optional procedures for field collection of population parameters, pathogen screening, and environmental parameters. In addition, methods for data management and analysis are recommended, as well as strategies for data dissemination and mapping. Finally, the third part provides information and support for cost estimates of the planned programmes and for the evaluation of the applied surveillance process.

**Conclusion:**

The ‘Guidelines for the surveillance of invasive mosquitoes in Europe’ aim at supporting the implementation of tailored surveillance of invasive mosquito species of public health importance. They are intended to provide support to professionals involved in mosquito surveillance or control, decision/policy makers, stakeholders in public health and non-experts in mosquito surveillance. Surveillance also aims to support control of mosquito-borne diseases, including integrated vector control, and the guidelines are therefore part of a tool set for managing mosquito-borne disease risk in Europe.

## Background

**Figure 1:**
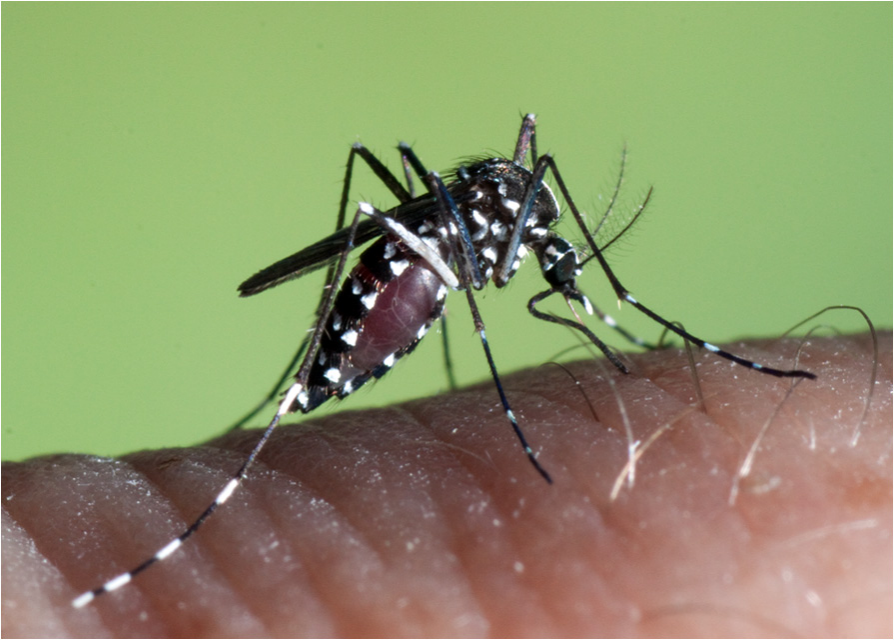
***Aedes albopictus*, female.** Source: F.Schaffner/ECDC.

Vector-borne diseases are a specific group of infections that present a (re-)emerging threat to Europe and therefore require particular attention [1]. The recent notifications of autochthonous transmission of dengue and chikungunya fevers in Europe [2-5], and the outbreak of dengue in Madeira [6], demonstrate the region’s vulnerability to these diseases in areas where an effective vector, *Aedes albopictus* (Skuse) (Figure 1) or *Aedes aegypti* Linnaeus, is present. Strengthening the surveillance of these two species as well as the other exotic and invasive mosquito species (Table 1), *Aedes atropalpus* (Coquillett), *Aedes japonicus japonicus* (Theobald), *Aedes koreicus* (Edwards) and *Aedes triseriatus* (Say) in areas at risk of importation or spread of mosquitoes and risk of virus transmission is therefore required [1]. This is particularly important in the context of environmental changes in, for example, land cover or weather patterns that might lead to an increase of vector populations, vector-host contact and virus amplification [7-9].

**Table 1:** Mosquito species names

**Traditional name (1906–2000)**	**Reinert *et al*. 2004**	**Reinert *et al*. 2006**
*Aedes (Stegomyia) aegypti*	*Stegomyia aegypti*	St. (Ste.) aegypti*
*Ae. (Ste.) albopictus*	*St. albopicta*	
*Ae. (Ochlerotatus) atropalpus*	*Ochlerotatus (Och.) atropalpus*	*Georgecraigius (Gec.) atropalpus*
*Ae. (Finlaya) japonicus*	*Oc. (Fin.) japonicus*	*Hulecoeteomyia japonica*
*Ae. (Fin.) koreicus*	*Oc. (Fin.) koreicus*	*Hl. koreica*
*Ae. (Protomacleaya) triseriatus*	*Oc. (Pro.) triseriatus*	

Major generic changes within the tribe Aedini were recently published [12-14], leading to scientific debate and two or more names being simultaneously used for a single taxon. In this article we use the traditional names [15,16], with alternate names shown in the table. * Subgenus *Stegomyia* re-defined in [17].

**Figure 2:**
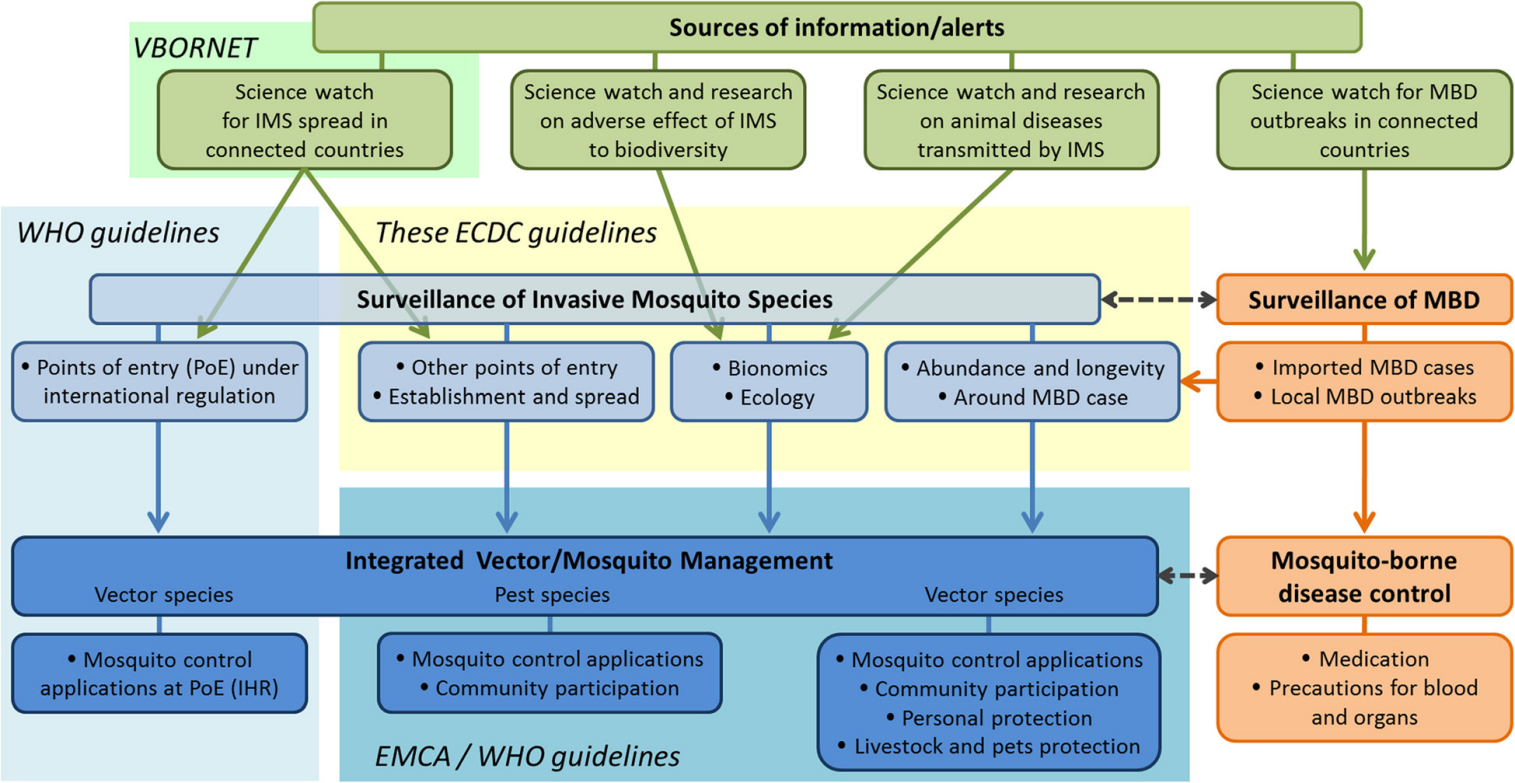
**Procedures and main issues of invasive mosquito species and mosquito-borne disease surveillance in Europe.** Green rounded rectangles show sources of information and alerts on risks for IMS and MBD that justify surveillance; part of it is performed within VBORNET, the European network of medical entomologists and public health experts (upper light green rectangle). These guidelines focus and develop the central part (central yellow rectangle). The left part is already addressed within the WHO guidelines (light blue rectangle), whereas the lower part is dealt with by the EMCA/WHO initiative on guidelines (dark blue rectangle). Blue rounded rectangles show procedures for surveillance (light blue) and control (dark blue) of IMS. Red rounded rectangles show procedures that are addressed within MBDs risk plans alongside IMS surveillance and control. *WHO Guidelines* (http://www.who.int/ihr/en): In the context of the application of the International Health Regulations (IHR 2005), WHO aims to strengthen national capacities by developing and updating guidelines and tools on vector surveillance and control. Thus, a web-based global point of entry (PoE) vector identification platform is under development, as well as a ‘Handbook on vector surveillance and control at points of entry’. This handbook focuses on actions that can be performed at PoE and on conveyances, containers, cargo, postal parcels and baggage. It considers all vector species (including mosquitoes) relevant to major MBDs. *EMCA/WHO Guidelines:* EMCA and WHO European Region have recently launched an initiative to develop ‘Guidelines for the control of invasive mosquitoes and associated vector-borne diseases on the European continent’, based on pan-European consultations. The first deliverable will be a strategy document with special emphasis on control issues.

Early detection of invasive mosquito species (IMS) enables appropriate and timely response measures and subsequent prevention of mosquito-borne disease (MBD) [10]. In addition, however, in areas where IMS have become established, timely surveillance of their abundance and spread is needed to assess the risk of pathogen transmission to humans [1]. In order to encourage the Member States to collect appropriate data on IMS in the field and further harmonise surveillance procedures within Europe, the European Centre for Disease Prevention and Control (ECDC) launched the production of ‘Guidelines for the surveillance of invasive mosquitoes in Europe’ as part of a toolset for assessing and controlling a number of risks posed by IMS and MBDs (Figure 2). This document describes the guidelines in the context of the key issues surrounding IMS surveillance in Europe.

## Methods

The mosquito species considered here are all exotic species that have been introduced into Europe in recent decades and have proven or are suspected to be invasive.

The proposed surveillance methods are applicable in the whole of geographical Europe (all European Union/European Economic Area and neighbouring countries), including European Union Outermost Regions, but they are not suitable for the Overseas Countries and Territories, which have different vector species, diseases, environment, and climate to the European continent.

**Figure 3:**
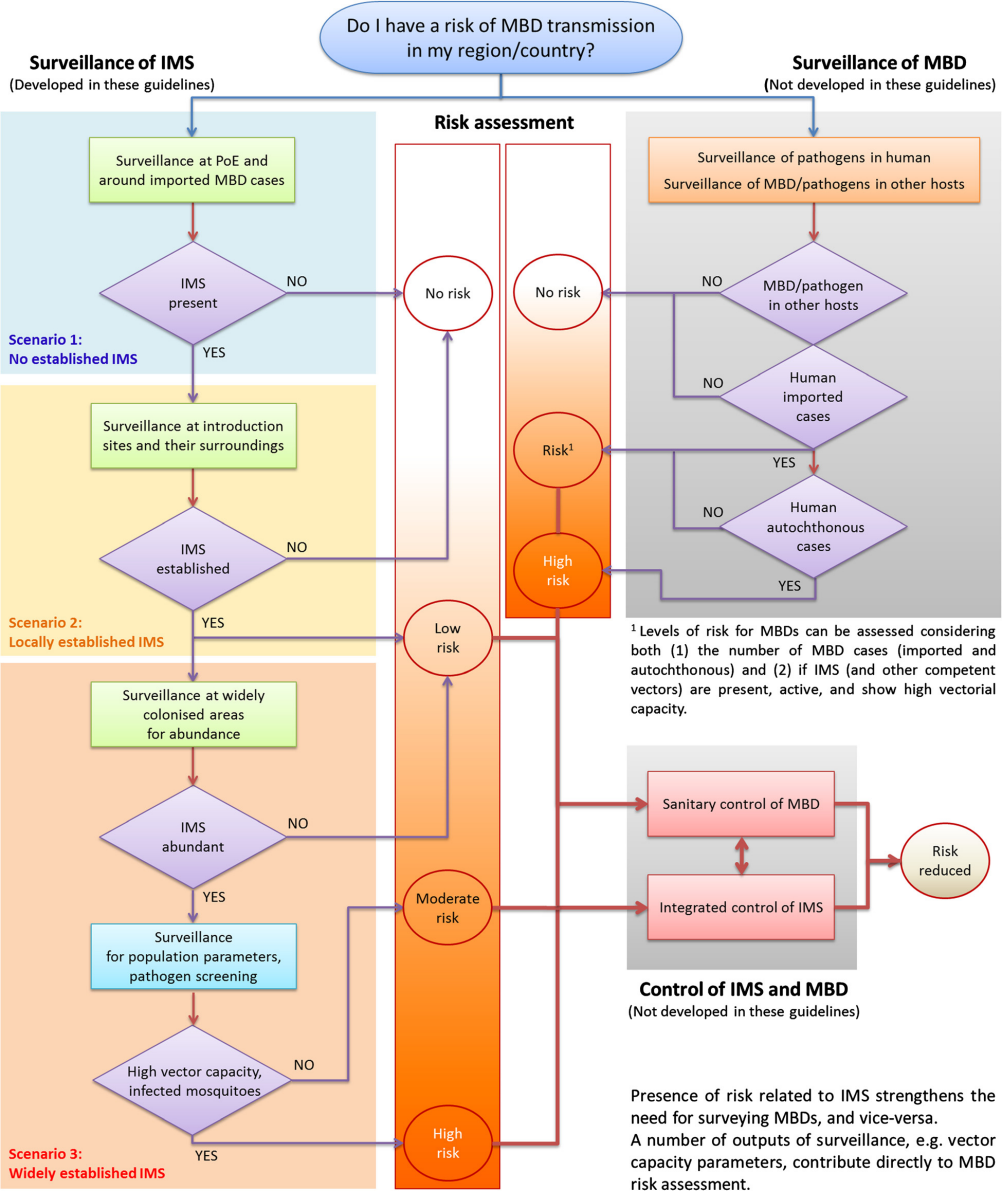
**Decision diagram for the implementation of surveillance of invasive mosquito species, in relation to mosquito-borne disease risk assessment and management.** The large blue, orange and red rectangles show activities and decisions related to IMS surveillance, that are covered by the three scenarios used for defining the surveillance strategies described in the guidelines. Grey rectangles show activities and decisions to be implemented alongside IMS surveillance, within MBD risk plans, including surveillance of MBDs and control of IMS and MBDs. Depending on the MBD, indigenous mosquitoes may also be targeted.

‘Surveillance’ (as opposed to ‘monitoring’), is defined here as a set of procedures developed in response to a recognised risk and carried out to support subsequent actions. Surveillance of mosquito vectors in Europe can therefore contribute to a global plan for risk assessment and management of MBDs (Figures 2, Figure 3).

### Development of these guidelines

In order to produce a draft version of these guidelines, ECDC launched an open call for tenders on 6 April 2011 (OJ/06/04/2011-PROC/2011/023). After a thorough evaluation of all applications, a contract was signed with the authors, representing a pan-European spectrum of complementary experience and knowledge in mosquito surveillance as applied to IMS.

The guidelines are based on a review of published and grey literature as well as on field experience of the contract team and external experts from two major European networks: VBORNET (the European network of medical entomologists and public health experts, http://www.vbornet.eu); and EMCA-AIM-WG (the *Aedes albopictus* and other invasive mosquitoes Working Group of the European Mosquito Control Association, http://www.emca-online.eu). Moreover, in order to obtain up-to-date information about mosquito surveillance activities in Europe, two missions were performed in Spain and Portugal: Spain has over five years experience of IMS surveillance, while Portugal has only recently implemented mosquito monitoring with little focus on IMS. An additional mission was carried out in the north-eastern United States (interviewing research units and mosquito control abatements from Connecticut, Michigan, and New Jersey) where some vectors, pathogens, and consequently surveillance strategies are different from those implemented in Europe.

A draft version of the guideline document was reviewed during an *ad hoc* meeting at ECDC in Stockholm. Entomologists, public health experts and end users from 17 EU/EEA and neighbouring countries (Albania, Austria, Belgium, Bulgaria, Croatia, Denmark, France, Germany, Greece, Italy, Portugal, Romania, Serbia, Spain, Switzerland, the Netherlands, and the United Kingdom) took part in the meeting to review, improve and agree on the guidelines [11]. As an outcome of this process, a final version of the guidelines was edited by ECDC (see Additional file 1).

## Results and discussion

### The guidelines

The guidelines provide accurate information and technical support for focused surveillance activities and data collection in the field. They also provide cost estimates and suggest adaptations according to the local context and the evolution of the epidemiological situation. They are intended to describe all procedures to be applied to the surveillance of IMS.

The first part addresses strategic issues and steps to be taken by the stakeholders for the decision-making process. According to the aim and scope of surveillance, advice is provided to define the organisation and management of the process, as well as the surveillance strategy to be developed. Three likely scenarios are proposed:

• Scenario 1 – No established IMS: There is a risk of introduction and establishment of IMS but this has not yet been reported. Surveillance activities are designed to detect possible introduction and establishment of IMS at specific points of entry.

• Scenario 2 – Locally established IMS: An IMS population is locally established in a small area, but with no evidence of spreading. Surveillance aims to quantify establishment and detect possible spread of IMS.

• Scenario 3 – Widely established IMS: At least one IMS population has colonised a large area by spreading locally. Surveillance aims to assess IMS population abundance and dynamics.

The risk estimate here is based on presence and abundance of IMS, not on the likelihood of transmission of MBDs. If the country already faces an outbreak of a MBD, then surveillance activities may need to be extended/strengthened, according to complementary guidance for the surveillance of MBDs and control of vectors and MBDs.

The second part addresses all operational issues and steps for the activities to be implemented, *i.e.* key procedures for field surveillance of IMS, methods of identification of IMS, key and optional procedures for field collection of population parameters, pathogen screening, and evaluation of environmental parameters. This part also recommends methods for data management and analysis, as well as strategies for data dissemination and mapping. Practical information is given in annexes, tailored to different audiences, *e.g*. general information on mosquito biology for non-entomologists, original mosquito identification keys for entomologists, practical tips for implementing trapping activities for field technicians.

Finally, the third part provides cost estimates for the planned programmes and sets out the procedures needed to evaluate the surveillance process. It aims at supporting planning and cost estimation prior to surveillance implementation, and at promoting surveillance evaluation and improvement/readjustment of the procedures.

The guidelines contribute to the harmonisation of surveillance methods and information records at the European level so that data and experience from different countries/areas can be compared over time. They are intended to provide support to professionals involved in implementing IMS surveillance or control; to decision- and policy-makers and stakeholders in public health; and also to non-experts in mosquito surveillance and control.

### Why survey mosquitoes in Europe?

Mosquitoes may be of public health relevance either when they transmit disease to humans, or when they occur in sufficient numbers to cause a nuisance. Both indigenous and invasive mosquito species comprise efficient vectors of pathogens (*e.g.* the Asian tiger mosquito, *Ae. albopictus*, is competent to transmit at least 22 arboviruses, and the common house mosquito *Culex pipiens pipiens* at least 6 arboviruses) as demonstrated by the recent outbreaks of chikungunya, dengue, and West Nile fevers in the Mediterranean basin [6,18,19]. In addition to viruses, mosquitoes may transmit malaria parasites (vector species belonging exclusively to the genus *Anopheles*) and dirofilaria worms in Europe. Indeed, the rapid spread of *Ae. albopictus* throughout Italy is likely to have broadened the range of *Dirofilaria immitis* and *D. repens* to include southern regions not previously infected despite the presence of *Culex pipiens pipiens*, which is considered the main indigenous vector of both *Dirofilaria* spp. in Europe [20]. The sympatric occurrence of both vectors, with both diurnal and nocturnal biting activities, may further enhance the risk of transmission to dogs and humans in many parts of Europe [20]. In recent decades, human contact with mosquitoes has become more frequent as suburbs that sprawl into previously undisturbed natural areas provide a greater number and variety of mosquito breeding places than do inner-city areas [21]. In addition, urbanised areas are facing invasion by container-breeding mosquitoes such as *Ae. albopictus* which has an aggressive nuisance behaviour during the day when females are seeking blood meals from humans and domestic animals.

### Why focus on invasive mosquitoes?

**Figure 4:**
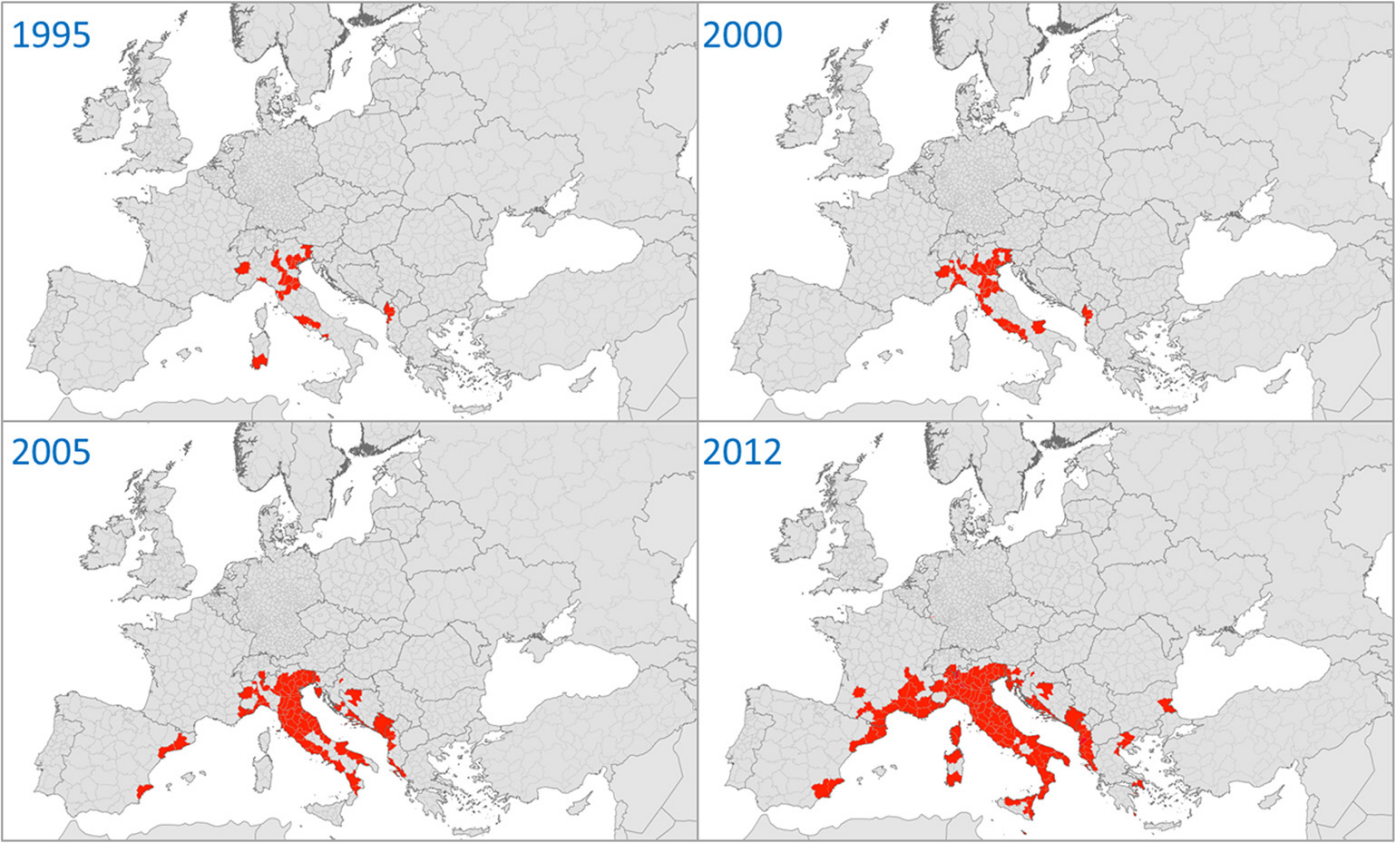
**Spread of the Asian tiger mosquito *Ae. albopictus *within Europe, 1995–2012.** Red mapping units (territorial units for statistics NUTS 3) = presence; grey units = absence or no available information. The figure has been adjusted and updated compared to the figure given in the guidelines.

**Figure 5:**
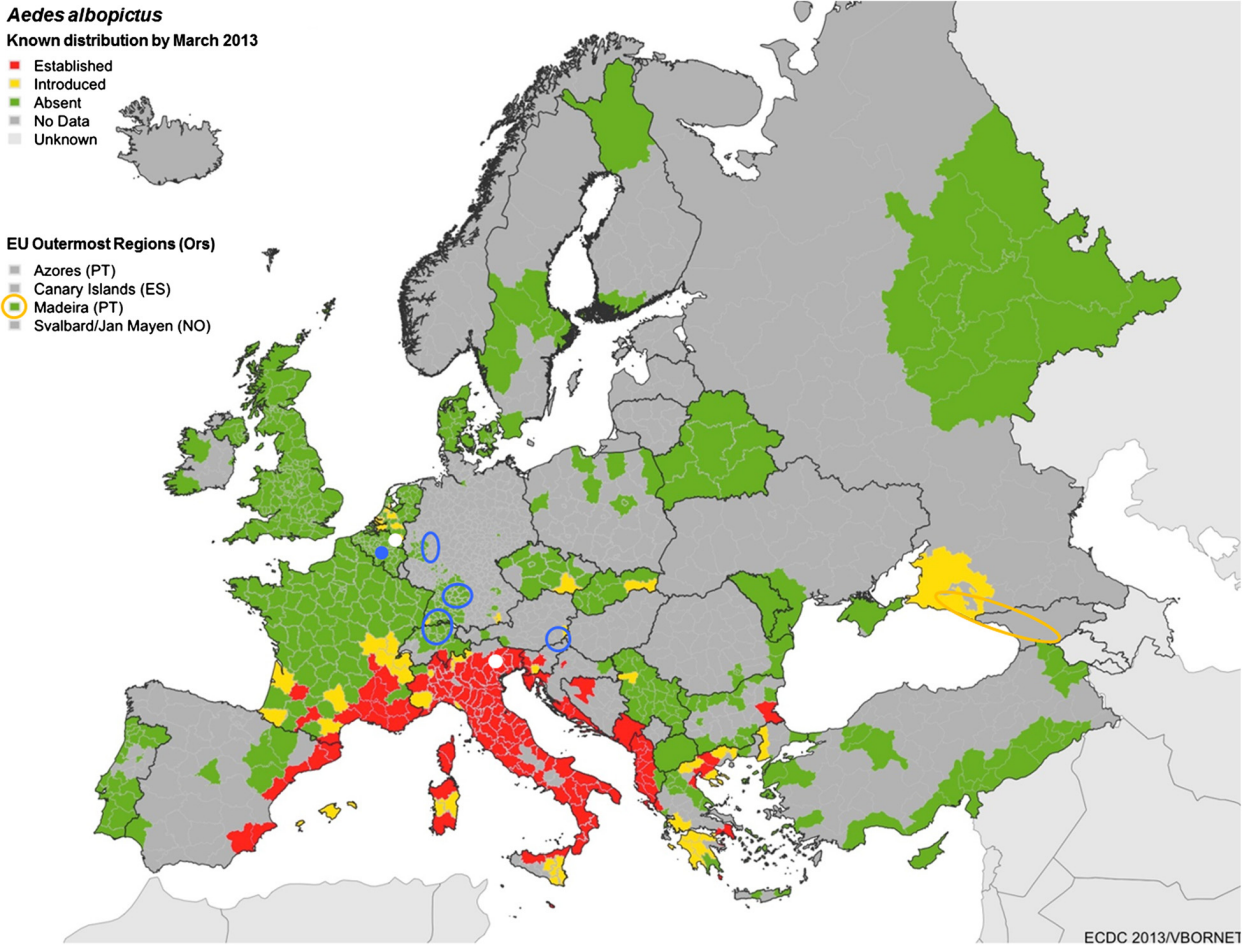
**Known distribution of targeted invasive mosquito species by March 2013 (with details on countries and first reports in legend).** Background map: distribution of ***Ae. albopictus*** (**red:** established; **yellow:** introduced, without confirmed establishment)**:** First reports: Albania 1979, Italy 1990, spreading into 11 countries of the Mediterranean; localized in Bulgaria 2011; sporadic records without confirmed establishment in Belgium 2000 (not shown), The Netherlands 2005–2012, Germany 2007/2011/2012, Serbia 2009/2011/2012, Turkey and Russia 2011, Austria, Czech Republic and Slovakia 2012. Mapping units used are territorial units for statistics NUTS 3. ‘Absent’ (green colour) means that surveillance of mosquitoes has been implemented during the last 5 years without reports of introduction or establishment. Other colours: see legend on the map. ***Ae. aegypti *(orange circles):** Russia 2001, Portugal-Madeira 2004, Abkhazia and Georgia 2007, The Netherlands 2010 [eliminated: not shown]; ***Ae. japonicus***, **(blue circles):** France 2000 [eliminated: not shown]), Belgium 2002 [localized], Switzerland and Germany 2008, Austria and Slovenia 2011; ***Ae. koreicus *(white dots):** Belgium 2008 [localized], Italy 2011. **Not shown: *Ae. atropalpus*:** Italy 1996 and France 2003 [eliminated], The Netherlands 2009-2011 [eliminated]; ***Ae. triseriatus*** France 2004 [intercepted].

IMS are defined by their ability to colonise new territories and to cause or to be likely to cause harm to the economy, environment, or human health [22]. Human activities are the primary means of IMS introduction. A considerable increase in the spread of IMS has been observed within Europe since the late 1990s, since then *Ae. albopictus* has continuously expanded its distribution (Figure 4) and several other container-breeding *Aedes* species have been reported from new countries every year (Figure 5; details about successive introductions and spread in Europe are given in [18]). To date, *Ae*. *albopictus* has colonised most Mediterranean countries, and the Asian bush mosquito *Ae. japonicus* is spreading widely in Central Europe. Two other species, *Ae. atropalpus* and *Ae. koreicus*, have been introduced on several occasions, leading to the establishment of populations at few foci. *Aedes triseriatus* was intercepted at a point of entry, and its establishment was prevented by the implementation of immediate control measures. Finally, the yellow fever mosquito *Ae. aegypti*, which had been introduced into Europe during the 17th-19th centuries existed in coastal areas of southern Europe until its disappearance during the 20th century, probably linked to malaria vector control activities and/or urbanisation and improvement of hygiene, especially in water-supply (piped water). This species has now returned, having recently become established on Madeira as well as around the Black Sea coast (Russia, Abkhazia, Georgia). These invasive mosquito species are well adapted to anthropogenic settings where they exploit the abundant sources of feeding, resting places and larval breeding sites (mainly man-made water containers) [18]. They may also reduce biodiversity as they outcompete native mosquito species, but the main hazard they pose is the threat to both human and animal health.

### Economic and social issues

A considerable amount of money is invested in reducing the nuisance caused by mosquitoes in Europe, mainly in tourist regions around the Mediterranean Sea, but also in flood plains (*e.g.* Danube, Po, Rhine, or Rhone valleys) and irrigated agricultural areas (*e.g*. northern Italy, northern Greece) [23]. Mosquito control is most often managed by public agencies implementing medium-term programmes. The arrival of IMS in cities and peri-urban areas can affect public perception of the effectiveness of control programmes already in place. Also control methods must be adapted to the mosquito species, as controlling mosquitoes in containers around human settlements is clearly different to controlling cohorts of flood plain/marshland mosquitoes, in terms of available techniques, equipment, and biocides. In addition, higher suppression efficiency will be expected for vector control during an outbreak compared to control of biting nuisance in a MBD-free context. Indeed, different types of organisations may be involved for different mosquito types. Local government and environmental agencies usually deal with nuisance species, whereas state and public health units are involved in the control of species that transmit pathogens.

Epidemics of MBDs may also have considerable economic impact. A burden of disease analysis performed on the chikungunya epidemic on La Réunion island in the Indian Ocean (2005–2006, 204,000 cases) estimated the total cost of medical expenses at 43.9 million euros, of which 60% was attributable to direct medical costs and 40% to the disease related loss of productivity [24]. This represents 56.10 euros per island inhabitant over two years. Besides medical costs, similarly high expenditures were involved in combating the disease (including vector control measures). These costs can be compared to cost of activities currently supported by the Emilia-Romagna region of Italy, where 5–6 million euros are spent yearly on a prevention plan for dengue and chikungunya (including the direct costs associated with surveillance, control and information management) [25]. This represents approximately 1.4 euros per person in the area at risk.

### Current impact of MBDs and threat for the future

Although MBDs (Table 2) currently represent a lower disease burden in temperate than in tropical regions where they have a substantial impact on the countries’ socio-economic development, there have always been both endemic and epidemic autochthonous MBDs in Europe. However, mosquitoes introduced into the area may increase risk to human health by (i) concurrently harbouring novel pathogens, (ii) transmitting native pathogens, or (iii) transmitting novel pathogens that were independently introduced [26].

**Table 2:** Important mosquito-borne pathogens that cause disease in humans

**Arboviruses**	**Transmission in Europe**	**Important vectors to human**
Chikungunya	Italy 2007; France 2010	*Ae. aegypti, Ae. albopictus*
Dengue 1–4	Until early 20th century in southern Europe; Croatia and France 2010, Portugal (Madeira) 2012	*Ae. aegypti, Ae. albopictus*
Eastern equine encephalitis, La Crosse encephalitis, Rift Valley fever	–	*Aedes* spp., *Culex* spp.
Japanese encephalitis, Murray Valley encephalitis, St Louis encephalitis, Ross River fever, Venezuelan equine encephalitis, Western equine encephalitis	–	*Culex* spp.
Sindbis	Endemic in northern Europe	*Ae. cinereus*, *Cx. pipiens*
West Nile	Endemic in southern Europe	*Cx. modestus, Cx. pipiens, Cx. perexiguus*
Yellow fever	Until 19th century, mainly in ports and occasionally inland in southern Europe	*Ae. aegypti*, *Ae. africanus*, *Haemagogus* spp.
***Plasmodium *protozoa**		
Malaria	Endemic until mid-20th century; since then sporadic cases; epidemic in Greece 2011, 2012	*Anopheles* spp.

In certain areas, IMS may remain undetected for a while, as for *Ae. japonicus* in Switzerland, where a first field investigation triggered by a citizen complaint revealed a colonised area of approximately 1,400 km^2^, suggesting that the species had been unnoticed for several years [27]. *Aedes albopictus* was present in Albania and Italy for 30 and 17 years, respectively, before the first outbreak of MBD attributed to this mosquito was reported in Italy. In France, however, autochthonous cases of chikungunya and dengue were detected only four years after the species was established. This suggests that the global context is becoming more favourable to pathogen introduction (*e.g*. frequency and intensity of epidemics in dengue-endemic areas) and that the local conditions that make the transmission of diseases carried by IMS possible are now frequently found in Europe. This is correlated with the vectorial capacity of the established mosquito populations and the frequency of vector-host contact [28]. Changes in eco-systems, land cover, human behaviour, and climate may impact MBD transmission [8,29]. Some of the factors affect several steps of the transmission cycle: for example, weather conditions may have a direct influence not only on the pathogen itself (*i.e.* higher temperatures allow a faster replication / dissemination of the pathogen in the mosquito) but also affect the vector’s reproduction, activity and survival [7,9,30]. These relationships can be used to extrapolate the future possible distribution of a mosquito species based on its ecological requirements and projected scenarios of climate change [31-34]. However, so far, human-induced environmental changes combined with globalisation and absence of or inefficient public health measures have been shown to be the primary driving forces for the emergence and global spread of dengue in the past 40 years [35].

## Conclusions

Mosquito-borne diseases are (re-)emerging threats to Europe. The collection of information and data on insect vectors are crucial to understand the levels of risk that countries face, and to define the actions that need to be taken. The ‘Guidelines for the surveillance of invasive mosquitoes in Europe’ aim to support the implementation of tailored surveillance of IMS of public health importance. They provide accurate information and technical support for focused field data collection, proposing adaptations dictated by the local context and the epidemiological situation, and taking into account estimated costs. They may also contribute to harmonising surveillance methods and information records at the European level so that data from different countries/areas can be compared over time and between different areas. They are also intended to provide support to non-experts in mosquito surveillance, stakeholders in public health, decision/policy makers, and professionals involved in implementing IMS surveillance or control.

Currently, the targeted mosquito species are all exotic invasive *Aedes* species that have been reported as introduced into Europe to date, including *Ae. aegypti*, *Ae. albopictus*, *Ae. atropalpus*, *Ae. japonicus*, *Ae. koreicus*, and *Ae. triseriatus*. They share the common traits of being container-breeding species, invasive, anthropophilic, and showing significant vectorial capacity. Of the range of pathogens that IMS can transmit, dengue and chikungunya are considered as the main threats to human health, and have been locally transmitted by *Ae. aegypti* and *Ae. albopictus* in Europe and outermost regions. Threats to animal health and to the environment (particularly to biodiversity) can also be addressed by adapting the surveillance methods described in these guidelines. The proposed methods are applicable in the whole of geographical Europe, including European Union Outermost Regions, but not Overseas Countries and Territories.

Surveillance of IMS aims to support MBDs control, including integrated vector control. Assessing and managing the risk of introduced MBDs that have become established in Europe is now a necessity and should also become a priority, in particular in countries where *Ae. albopictus* and/or other IMS are established. The guidelines are therefore part of a tool set for managing MBD risk in Europe. A first evaluation of these guidelines has been performed in Belgium within a pilot study implemented in 2012 and results will be published elsewhere. Further updates are scheduled for three-year intervals, or whenever a major change in vector fauna or MBD risk occurs.

## Competing interests

The authors declare that they have no competing interests.

## Authors’ contributions

All authors contributed to writing the manuscript. LMR managed the production of guidelines, FS co-ordinated the writing phase. FS, RB, DP, and EJS wrote the guidelines, FS produced the figures. LMR and HZ performed the day-to-day reviewing and organised the internal (ECDC) and external reviewing process, and the editorial work at ECDC (Additional file 1). All authors read and approved the final version of the manuscript.

## Supplementary Material

Additional file 1 Content: ECDC guidelines for the surveillance of invasive mosquitoes in Europe; Full content. Click here for file
