# Supplementary material for: Development of guidelines for the surveillance of invasive mosquitoes in Europe
Source: Parasit Vectors. 2013 Jul 18;6:209. doi: 10.1186/1756-3305-6-209 (PMC3724590; doi:10.1186/1756-3305-6-209)
Supplement: Additional file 1 — Content: ECDC guidelines for the surveillance of invasive mosquitoes in Europe; Full content. [file 1756-3305-6-209-S1.pdf]

ecdc
EUROPEAN CENTRE FOR DISEASE PREVENTION AND CONTROL

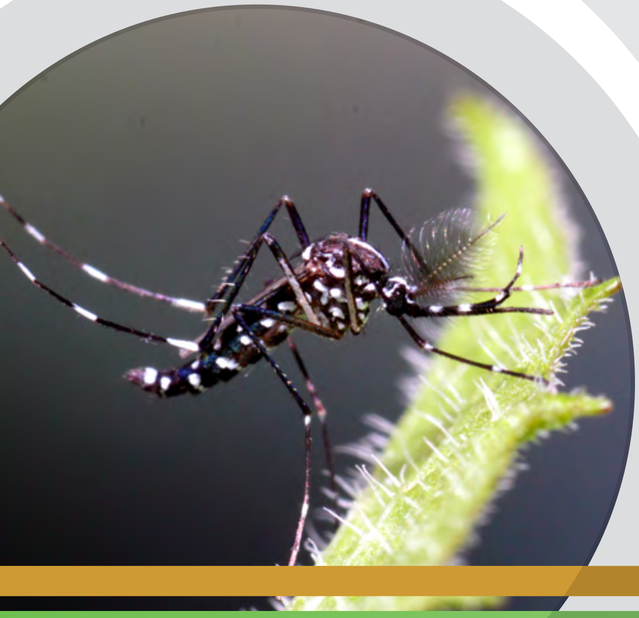

TECHNICAL REPORT

Guidelines for the surveillance of invasive mosquitoes in Europe

www.ecdc.europa.eu

ECDC TECHNICAL REPORT

Guidelines for the surveillance of invasive mosquitoes in Europe

EUROPEAN CENTRE FOR DISEASE PREVENTION AND CONTROL

This report was commissioned by the European Centre for Disease Prevention and Control (ECDC), coordinated by Laurence Marrama-Rakotoarivony and Hervé Zeller, and produced under contract ECD.2842 by Francis Schaffner (Avia-GIS bvba, Belgium), Romeo Bellini (Centro Agricoltura Ambiente 'G. Nicoli', Italy), Dušan Petrić (University of Novi Sad, Serbia), Ernst-Jan Scholte (National Centre for Monitoring of Vectors, Netherlands), with contribution of experts from Portugal, Spain, and the USA.

Acknowledgements

We would like to thank Willy Wint for his help in editing the text.

Note

A draft version of this guideline document was reviewed and improved during an ad hoc meeting by entomologists, public health experts, and end users from most of the EU countries.

Suggested citation: European Centre for Disease Prevention and Control. Guidelines for the surveillance of invasive mosquitoes in Europe. Stockholm: ECDC; 2012.

Stockholm, August 2012

ISBN 978-92-9193-378-5

doi 10.2900/61134

© European Centre for Disease Prevention and Control, 2012

Reproduction is authorised, provided the source is acknowledged.

Contents

Abbreviations iv
Glossary iv
Executive summary 1
Introduction 4

1 Surveillance of invasive mosquitoes: the decision-making process 9
  1.1 Aim and definition of scope 9
  1.2 Organisation and management 10
  1.3 Surveillance strategies 12

2 Operational process: The surveillance of invasive mosquitoes 14
  2.1 Methods of collection of invasive mosquitoes 14
  2.2 Methods of identification of invasive mosquitoes 17
  2.3 Procedures for the determination of mosquito population parameters 21
  2.4 Procedures for mosquito pathogen screening and identification 24
  2.5 Procedures for the collection of environmental parameters 26
  2.6 Methods for data management and analysis 28
  2.7 Strategies for data dissemination and mapping 30

3 Cost and evaluation of the defined surveillance programme 33
  3.1 Estimating cost of surveillance activities 33
  3.2 Evaluation of the operational surveillance process 35

Annex 1: Basic information on the biology of mosquitoes and the transmission of vector-borne diseases 36
Annex 2: Organisation and management of IMS surveillance in the European context 40
Annex 3: Detailed procedures for surveying IMS 43
Annex 4: Identification of invasive mosquito species 63
Annex 5: Principles and detailed methodologies for the determination of mosquito population parameters 73
Annex 6: Complementary information on methods for pathogen screening in field collected mosquitoes 83
Annex 7: Complementary information on measurement of environmental parameters 86
Annex 8: Complementary information on data management and analysis 90
Annex 9: Complementary information on methods of dissemination of surveillance results 91

Abbreviations

CDC	Centers for Disease Control and Prevention, USA
EDEN	FP6 research programme ‘Emerging diseases in a changing European Environment’
EDENext	FP7 research programme ‘Biology and control of vector-borne infections in Europe’
EEA/EFTA	European Economic Area/European Free Trade Association
EMCA	European Mosquito Control Association
EMCA-AIM-WG	EMCA ‘*Aedes albopictus* and other invasive mosquitoes’ working group
ENIVD	European Network for Diagnostics of ‘Imported’ Viral Diseases
EU	European Union
IHR	International Health Regulations
IMS	Invasive mosquito species
IPCC	International Panel on Climate Change
KOM	Kick-off meeting
MBD	Mosquito-borne disease
MIR	Minimum infection rate
MoH	Ministry (in charge) of health
NUTS	Nomenclature of Territorial Units for Statistics
PH	Public health
PoE	Point of entry
SOVE	Society for Vector Ecology
VBD	Vector-borne disease
VBORNET	European Network for Arthropod Vector Surveillance for Human Public Health
WHO	World Health Organization

Glossary

An **exotic** plant or animal species (synonyms: alien, foreign, non-indigenous, non-native) is a species that is not native to an ecosystem and, if present, has been introduced.

An **invasive** species is an exotic species that establishes and proliferates within an ecosystem, and whose introduction causes or is likely to cause economic or environmental impact or harm to human health.

A **native** or **indigenous** species is a species that occurs within its natural geographical range (past or present) and dispersal potential (i.e. within the range it occupies naturally or could occupy without direct or indirect introduction or other human intervention).

Introduction is the process of bringing a species from its endemic range into a biogeographic area to which it is completely foreign.

Interception is the detection and elimination of an exotic species during inspection of an imported consignment, preventing establishment of the species.

Establishment is the perpetuation, for the foreseeable future, of an invasive species within an area following introduction.

Surveillance consists of procedures developed in response to a risk and carried out to support subsequent actions.

Monitoring consists of procedures implemented for temporary or continuous observation (e.g. of species dynamics) and is not followed by any additional activities.

Executive summary

Vector-borne diseases are a specific group of infections that present a (re-)emerging threat to Europe and require particular attention. The recent notifications of autochthonous transmission of dengue fever and chikungunya fever cases in Europe show its vulnerability to these diseases in areas where the vector, the invasive mosquito *Aedes albopictus*, is present. Strengthening surveillance of exotic mosquito species such as *Ae. albopictus*, *Ae. aegypti*, *Ae. atropalpus*, *Ae. japonicus*, *Ae. koreicus* and *Ae. triseriatus* in areas at risk of importation or spread of mosquitoes and risk of virus transmission is therefore required. This is particularly important in the context of environmental and climate changes which might allow an increase of vector populations and virus amplification.

Figure 1: Known distribution of targeted invasive mosquito species, June 2012

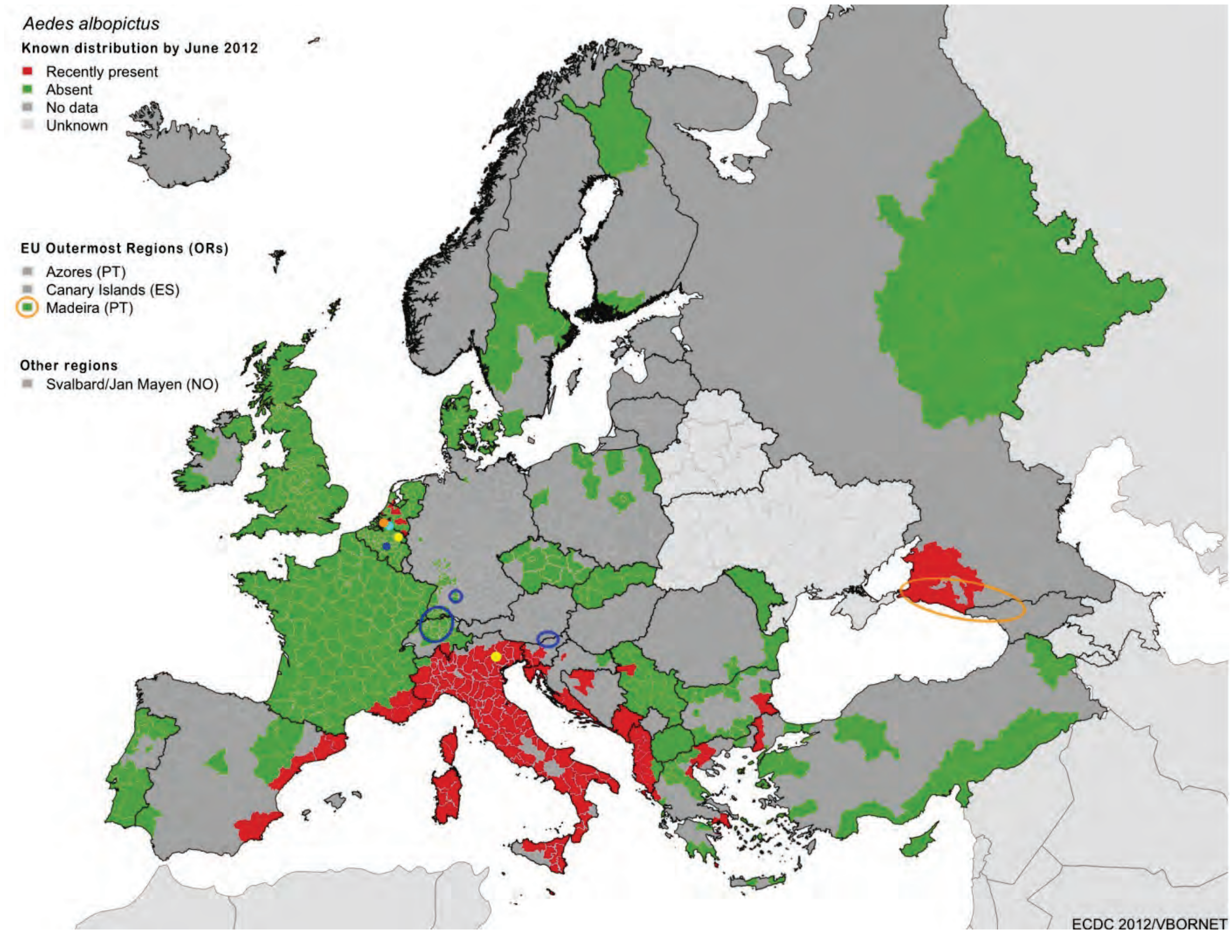

Distribution of Ae. albopictus *(red): first reports: Albania 1979, Italy 1990, spreading into 11 countries of the Mediterranean; introduced (and now with limited local distribution in the Netherlands) in 2005, Serbia 2009, Bulgaria, Russia and Turkey 2011; introduced but not established in Belgium in 2000 and Germany in 2007 and 2011.*
'Absent' means that surveillance of mosquitoes has been conducted over the last five years, with no reports of establishment. Mapping units used are NUTS 3. Other colours: see legend.

Ae. aegypti *(orange dot/oval): Russia 2001, Portugal-Madeira 2004, Abkhazia and Georgia 2007, Netherlands 2010 [limited local distribution];*
Ae. atropalpus *(light blue dot): Italy 1996 and France 2003 [eliminated], Netherlands 2009 [limited local distribution];*
Ae. japonicus*, (dark blue dot/oval): France 2000 [eliminated], Belgium 2002 [localised], Switzerland and Germany 2008, Austria and Slovenia 2011;*
Ae. koreicus *(yellow dot): Belgium 2008 [limited local distribution], Italy 2011.*
Not shown: Ae. triseriatus *France 2004 [intercepted].*

ECDC's programme on emerging and vector-borne diseases is developing timely and topical assessments of the risks that vector-borne diseases pose to citizens of the European Union and aims to provide Member States with practical tools and accurate information to support their decision making. In line with these objectives, ECDC identified the need for guidance on customised surveillance methods that encourage the Member States to collect appropriate data on invasive mosquito species (IMS) in the field. Early detection of invasive mosquitoes increases the opportunity for appropriate and timely response measures and therefore mosquito-borne disease (MBD)

prevention. In addition, in areas where invasive mosquito species have become established, surveillance of their abundance and further spread is needed for timely risk assessment of pathogen transmission to humans.

In order to further harmonise surveillance procedures within Europe, ECDC has produced these guidelines which aim to support the implementation of tailored surveillance for invasive mosquito species of public health relevance. This guideline document provides accurate information and technical support for focused data collection in the field, gives cost estimates, and suggests adaptations according to the evolution of the epidemiological situation. It also contributes to the harmonisation of surveillance methods and information records at EU level so that data from different countries/areas can be compared over time. It intends to provide support to professionals involved in implementing IMS surveillance or control, to decision- and policy-makers and stakeholders in public health, but also to non-experts in mosquito surveillance and control. The suggested methods may be applicable in the whole geographic area of Europe (thus in all EU Member States and EEA/EFTA countries, including EU Outermost Regions (ORs)), but not Overseas Countries and Territories (OCTs).

This guideline document describes all procedures to be applied to the surveillance of IMS.

The first part of these guidelines address strategic issues and steps to be taken by the stakeholders for the decision-making process, depending on the aim and scope of surveillance, its organisation and management, and the surveillance strategy to be developed. Three likely scenarios could be identified:

- Scenario 1 – no established IMS (but with risk of introduction and establishment)
- Scenario 2 – locally established IMS
- Scenario 3 – widely established IMS

The risk estimate is here based on presence and abundance of mosquitoes, not on the likelihood of transmission of MBD. If the country already faces an outbreak of MBD, then surveillance activities may need extending/strengthening, according to complementary guidance for the surveillance of MBD and control of vectors and MBD.

A second part addresses all operational issues and steps to be implemented by professionals involved in the applied process, i.e. key procedures for field surveillance of IMS, methods of identification of IMS, key and optional procedures for field collection of population parameters, pathogen screening, and environmental parameters. This part also recommends methods for data management and analysis, as well as strategies for data dissemination and mapping.

Finally, a third part provides cost estimates for the planned programmes and the evaluation of the applied surveillance process.

Box 1: Surveillance of invasive mosquito species (IMS), in relation to mosquito-borne disease (MBD) risk assessment and management

Surveillance of mosquitoes is part of the global response to MBD. Risk assessment and management of threats to human or animal health includes several activities (e.g. surveillance of human health and of intermediate hosts), but surveillance and control of the vector are crucial elements. In the epidemiology of a MBD and in particular for IMS, the vector may be the critical link, so that removing the vector prevents new cases.

Figure 2: Decision diagram for the implementation of surveillance of IMS, in relation to MBD risk assessment and management

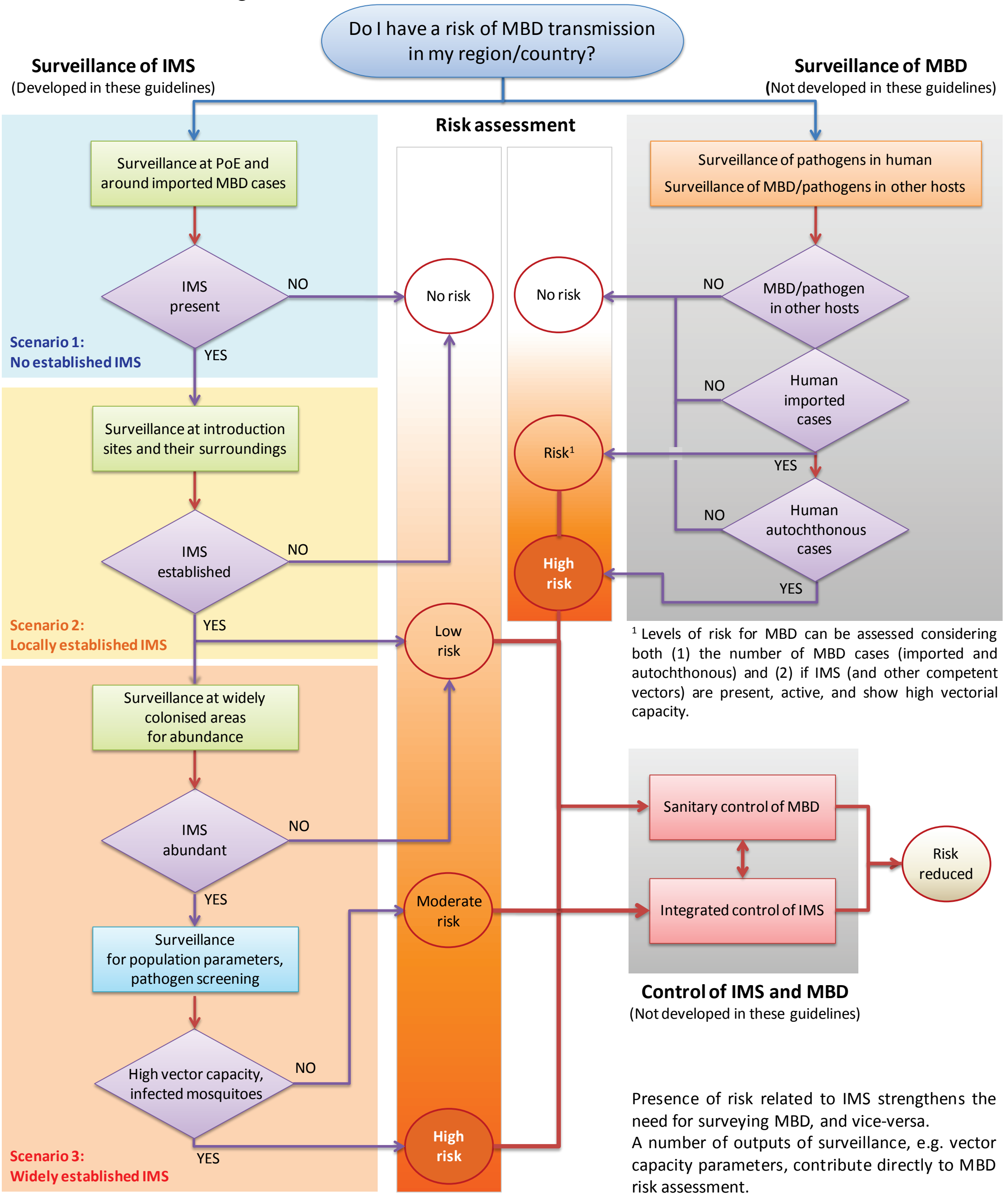

[1] Levels of risk for MBD can be assessed considering both (1) the number of MBD cases (imported and autochthonous) and (2) if IMS (and other competent vectors) are present, active, and show high vectorial capacity.

Presence of risk related to IMS strengthens the need for surveying MBD, and vice-versa.
A number of outputs of surveillance, e.g. vector capacity parameters, contribute directly to MBD risk assessment.

The large blue, orange and red rectangles show activities and decisions related to IMS surveillance that are covered by the three scenarios that were developed to define the surveillance strategy described in this guideline document (see Chapter 1.3). Grey rectangles show activities and decisions to be implemented alongside IMS surveillance, within MBD risk plans, including surveillance of MBD and control of IMS and MBD. Depending on the MBD, indigenous mosquitoes may also be targeted.

Introduction

Mosquitoes and mosquito-borne diseases in Europe: a changing picture

Mosquitoes may be of public health relevance either when they occur in such great density that they cause a nuisance or when they transmit disease agents. Over the last decades, human contact with mosquitoes has become more frequent as suburbs that sprawl into previously undisturbed natural areas provide a greater number and variety of mosquito breeding places than inner-city areas. In addition, urbanised areas are facing an invasion of container-breeding mosquitoes such as the Asian tiger mosquito *Aedes albopictus* which shows an aggressive nuisance behaviour during the day when females are seeking blood meals from humans. Invasive mosquitoes are often also efficient vectors of disease (e.g. *Ae. albopictus* is competent to transmit at least 22 arboviruses) as demonstrated by the recent outbreaks of chikungunya and dengue fevers in the Mediterranean.

Why survey for invasive mosquitoes?

Invasive mosquito species (IMS) are defined by their ability to colonise new territories. Human activity, especially the global movement of trade goods, has led to the passive dispersion of species previously confined to specific regions. A considerable increase in the spread of IMS has been observed within Europe since the late 1990s, with the Asian tiger mosquito *Ae. albopictus* having continuously expanded its distribution (Figure 3) and several other container-breeding *Aedes* species being reported from new countries almost every year (Figure 1). These invasive mosquito species may outcompete native mosquito species, but the main hazard is the threat to both human and animal health.

Figure 3: Spread of the Asian tiger mosquito *Ae. albopictus* in Europe, 1995–2011

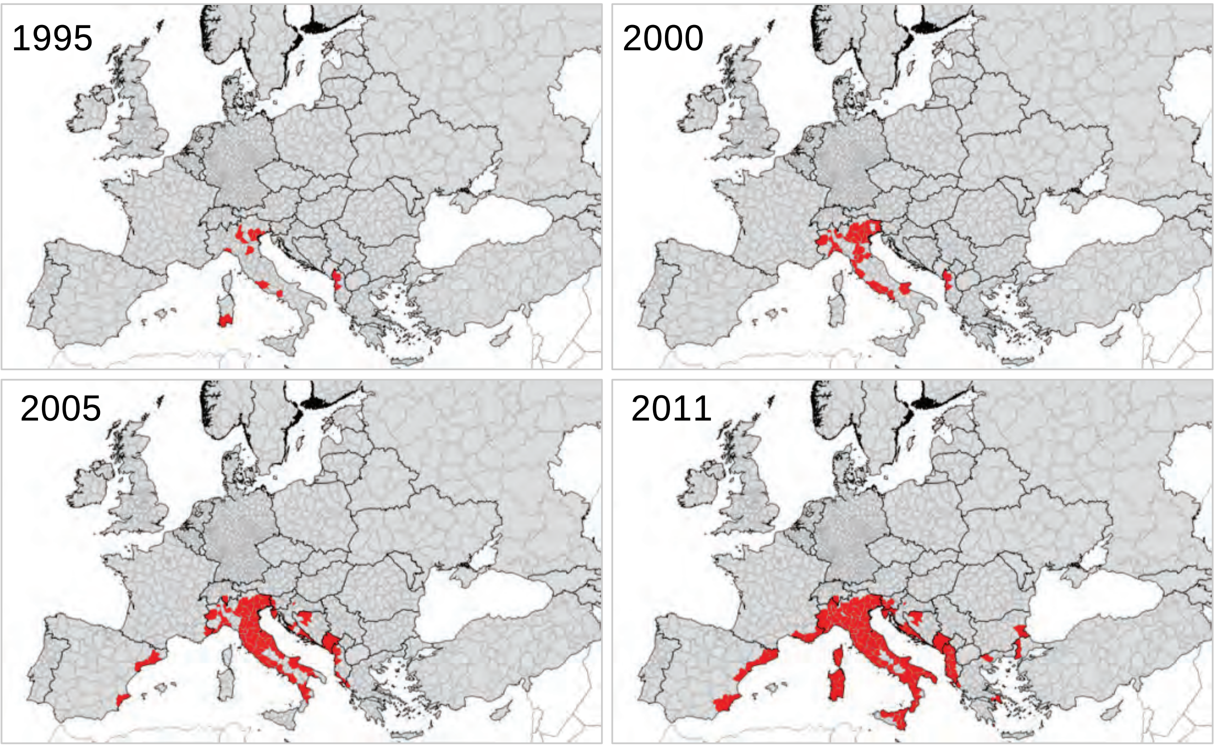

Red mapping units (NUTS 3) = presence; grey units = absence or no available information

Although mosquito-borne diseases (MBD; Table 1) represent a far higher burden in tropical than temperate regions, with a substantial impact on the countries' socio-economic development, there have always been both endemic and epidemic autochthonous MBD in Europe. Concern is now rising as both vectors and pathogens are increasingly being introduced by international travel and trade. Some of these diseases are emerging or are reappearing after a long absence, others are spreading. Their occurrence is often associated with changes in eco-systems, human behaviour, and climate. This is illustrated by the recently reported epidemics of chikungunya and West Nile viruses. In addition to viruses, mosquitoes may transmit malaria parasites and dirofilarial worms in Europe. Numbers of autochthonous infections, though still low, appear to be increasing. Assessing and managing the risk of introduced

MBD that have become established in Europe is now a necessity and should also become a priority, in particular in countries where *Ae. albopictus* and/or other IMS are established.

Table 1: Important mosquito-borne pathogens that cause disease in humans

Pathogens and diseases	Transmission in Europe	Important vectors to human
**Arboviruses**		
Chikungunya	Italy 2007; France 2010	*Ae. aegypti, Ae. albopictus*
Dengue 1–4	Until early 20th century; Croatia and France 2010	*Ae. aegypti, Ae. albopictus*
Eastern equine encephalitis, La Crosse encephalitis, Rift Valley fever	–	*Aedes spp., Culex spp.*
Sindbis	Endemic in northern Europe	*Aedes cinereus, Cx. pipiens*
Japanese encephalitis, Murray Valley encephalitis, St Louis encephalitis, Ross River fever, Venezuelan equine encephalitis, Western equine encephalitis	–	*Culex spp.*
West Nile	Endemic in southern Europe	*Cx. pipiens, Cx. modestus*
Yellow fever	Until 19th century, in ports	*Ae. aegypti, Ae. africanus, Haemagogus spp.*
***Plasmodium* protozoa**		
Malaria	Endemic until mid-20th century; since then sporadic cases; epidemic in Greece 2011	*Anopheles spp.*

More basic information on MBD and the biology of mosquitoes can be found in Annex 1.

Incidence and future of IMS

A considerable amount of money is invested in reducing the nuisance caused by mosquitoes in Europe, mainly in tourist regions around the Mediterranean, but also in flat plains (e.g. Rhone, Rhine or Danube valleys) and irrigated agriculture areas (e.g. northern Italy, northern Greece). Mosquito control is most often managed by public agencies implementing medium-term programmes. The arrival of IMS in cities can affect public perception of the effectiveness of control programmes already in place. Controlling a mosquito that breeds in containers around human settlements and potentially generates MBD transmission is completely different to controlling cohorts of marshland mosquitoes that only occasionally reach the cities and which transmit few MBD. Indeed, different types of organisations may be involved for different mosquito types. Local government and environmental agencies usually deal with nuisance species, whereas state and public health units are involved with species that transmit disease.

Epidemics of MBD may also have considerable economic impact. A cost-of-illness analysis performed on the chikungunya epidemic on La Réunion island (2005–2006, 204 000 cases) estimated the total cost of medical expenses at 43.9 million euros, of which 60% was attributable to direct medical costs and 40% to the disease-related loss of productivity. This represents 56.10 euros per island inhabitant. Besides medical costs, similarly high expenditures were involved in combating the disease (including vector control measures). MBD outbreaks may also affect blood supplies and organ donations. These costs can be compared to cost of activities currently supported by the Emilia-Romagna region of Italy, where 5.5 million euros were spent in 2011 on a prevention plan for dengue and chikungunya (including the direct costs associated to monitoring, control and information). This represents 1.4 euros per person in the area at risk.

IMS may remain undetected for a while in certain areas, as was the case for *Ae. japonicus* in Switzerland, where a first field investigation triggered by a complaint revealed a colonised area of approximately 1 400 km^2, suggesting that the species had been unnoticed for several years. The tiger mosquito *Ae. albopictus* was present in Albania and Italy for 30 and 17 years, respectively, before the first outbreak of MBD attributed to it was reported. In France, however, autochthonous cases of chikungunya and dengue were detected only four years after the species was established. This suggests that the factors which make the transmission of diseases carried by IMS possible are now frequently found in Europe. These factors are first related to the introduction of pathogens, the vectorial capacity of the established mosquito populations, and the frequency of vector-host contact. Climatic conditions may also have a direct influence not only on the pathogen itself (i.e. higher temperatures allow a faster replication/dissemination of the pathogen in the mosquito) but also affect the vector's reproduction, activity and survival. These relationships can be used to extrapolate the future possible distribution of a mosquito based on its ecological requirements and projected scenarios of climate change (Figure 4).

Figure 4: Suitability maps for the presence of *Ae. albopictus* using multi-criteria decision analysis, for current climate (left) and for IPCC climate change scenario, long-term (2030) and minimal impact

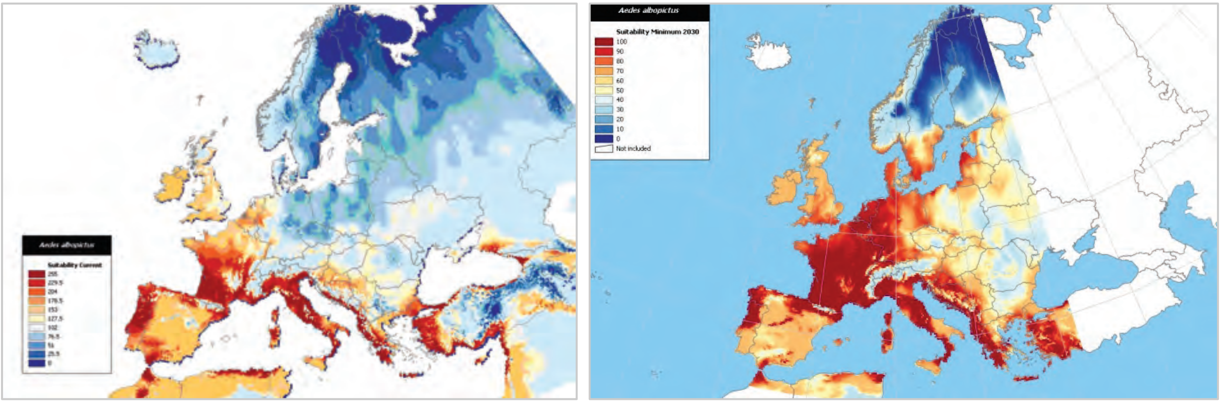

Predictor variables are annual precipitation, summer temperature, and temperature in January. Colours show the suitability levels, from the less suitable areas (dark blue) to the most suitable areas (dark red).

Source: European Centre for Disease Prevention and Control. Development of Aedes albopictus *risk maps. Stockholm: ECDC; 2009.*

Rationale and scope

Vector-borne diseases (VBD) are (re-)emerging threats to Europe. The collection of information and data on vectors of public health significance are of crucial importance to understand the levels of risk that countries face, and to define the actions that need to be taken.

These guidelines aim to support the implementation of tailored surveillance for invasive mosquito species (IMS) of public health relevance. It provides accurate information and technical support for focused field data collection, proposing adaptations dictated by the epidemiological situation and taking into account estimated costs. It may also contribute to harmonising surveillance methods and information records at the EU level so that data from different countries/areas can be compared over time. It intends to provide support to non-experts in mosquito surveillance, stakeholders in public health, decision/policy makers, and professionals involved in implementing IMS surveillance or control.

The targeted mosquito species are all exotic invasive *Aedes* species that have been reported as introduced into Europe to date, including *Aedes aegypti*, *Ae. albopictus*, *Ae. atropalpus*, *Ae. japonicus*, *Ae. koreicus*, and *Ae. triseriatus* (see Annex 1 for other names in use and Figure 1 for their known current distribution in Europe). They share the common traits of being container-breeding species, invasive, anthropophilic, and showing significant vectorial capacity. *Aedes aegypti* and *Ae. albopictus* are so far the only confirmed vectors of dengue and chikungunya occurring in Europe. Of the range of pathogens that IMS could transmit these two diseases are considered as the main threat to human health. Threats to animal health and to the environment (to biodiversity) can also be addressed by adapting the surveillance methods described in this guideline document. The proposed methods are applicable in the whole of geographical Europe (all EU Member States and EEA/EFTA countries), including EU Outermost Regions (ORs), but not Overseas Countries and Territories (OCTs).

Also addressed is the 'surveillance' of mosquitoes (as opposed to 'monitoring'), defined as a set of procedures developed in response to a recognised risk and carried out to support subsequent actions (cf. Glossary). Surveillance of mosquito vectors may be part of a global plan for risk assessment and management of MBD (Box 1). This guideline document is the final part of an ECDC-developed toolset for assessing and controlling a number of risks posed by invasive mosquitoes (Figure 5).

Development of these guidelines

In order to produce a draft version of this guideline document, ECDC launched an open call for tenders on 6 April 2011 (OJ/06/04/2011-PROC/2011/023).

After a thorough evaluation of all applications, a contract was signed with a team of experts from Belgium, Italy, Serbia and the Netherlands, representing a pan-European spectrum of complementary experience and knowledge in mosquito surveillance as applied to invasive species.

This guideline document is based on a review of published and grey literature as well as on field experience from the contract team and external experts from two major European networks (VBORNET and EMCA-AIM-WG). In order to gain up-to-date information about mosquito surveillance activities in Europe, two missions were performed in Spain and Portugal: Spain has long-standing experience (> five years) in invasive mosquito monitoring, while

Portugal has only recently implemented mosquito surveillance. An additional mission was carried out in the United States (New Jersey) where surveillance strategies are different from those implemented in Europe.

A draft version of this guideline document was reviewed during an ad hoc meeting in Stockholm organised by ECDC. Entomologists, public health experts and end users from 17 EU, EFTA and candidate countries (Albania, Austria, Belgium, Bulgaria, Croatia, Denmark, France, Germany, Greece, Italy, Portugal, Romania, Serbia, Spain, Switzerland, the Netherlands, and the United Kingdom) took part in the meeting to review, improve and agree on the guidelines (see meeting report on ECDC website). This version represents the updated guideline document.

Updates are planned after the completion of a pilot study in a yet to be determinded European country. Further updates are scheduled for three-year intervals, or whenever a major change in vector fauna or mosquito-borne disease risk occurs.

Figure 5: Procedures and main issues of IMS and MBD surveillance

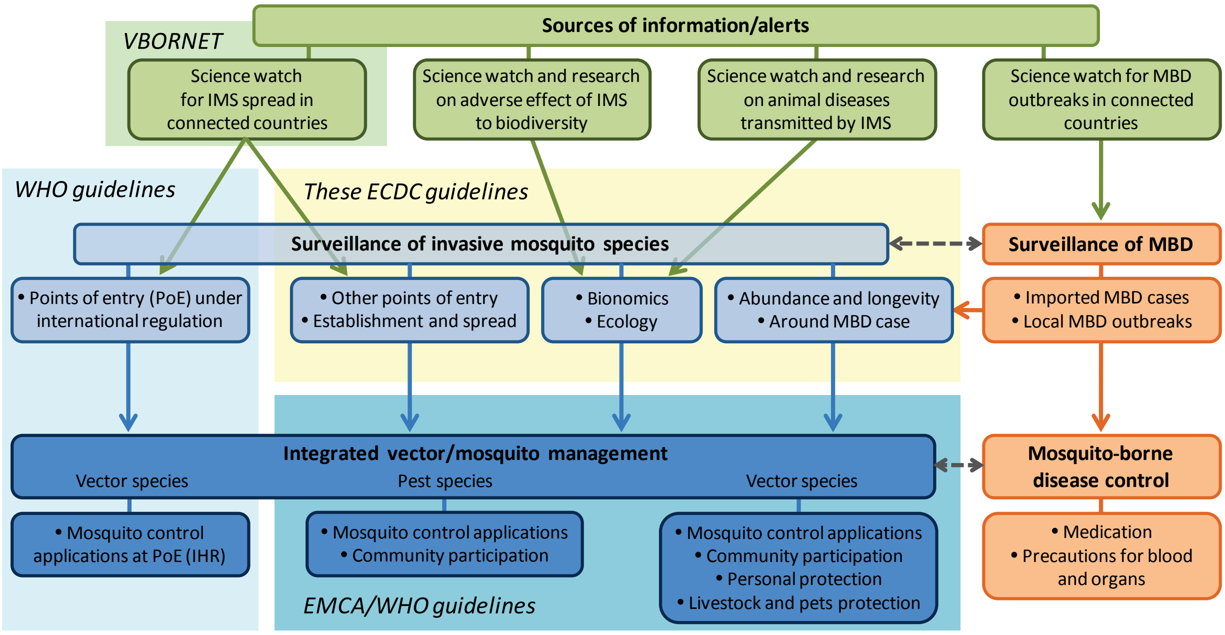

WHO guidelines (www.who.int/ihr/en)

In the context of the application of the International Health Regulations (IHR 2005), WHO aims to strengthen national capacities by developing and updating guidelines and tools on vector surveillance and control. Thus, a Web-based global point of entry (PoE) vector identification platform is under development, as well as a 'Handbook on vector surveillance and control at points of entry'. This handbook focuses on actions that can be performed at PoE and on conveyances, containers, cargo, postal parcels and baggage. It considers all vector species (including mosquitoes) relevant to major vector-borne diseases.

EMCA / WHO guidelines (www.emca-online.eu)

EMCA and WHO have recently launched an initiative to develop 'Guidelines for the control of invasive mosquitoes and associated vector-borne diseases on the European continent', based on pan-European consultations. The first deliverable is a strategic document with special emphasis on control issues.

This guideline document focuses on the central part of the above diagram (central yellow rectangle). The left part is already addressed by the WHO guidelines (light blue rectangle), whereas the lower section will be addressed by the EMCA/WHO initiative on guidelines (deep blue rectangle). Blue rounded rectangles show procedures for surveillance (light blue) and control (deep blue) of IMS. Orange rounded rectangles show procedures which are addressed within risk plans other than IMS surveillance and control. Green rounded rectangles show sources of information and risk alerts for IMS and MBD; some of these tasks are performed by VBORNET (upper light green rectangle).

This guideline document describes all procedures to be applied to the surveillance of IMS (Figure 6).

Chapter 1: Strategic issues and steps to be taken by stakeholders for the decision-making process, depending on the aim and scope of surveillance (1.1), its organisation and management (1.2), and the surveillance strategy to be developed – based on three basic scenarios (1.3).

Chapter 2: Operational issues and options to be implemented by professionals involved in the operational process: key procedures for field surveillance of IMS (2.1); methods of IMS identification (2.2); key and optional procedures for field collection of population parameters (2.3); pathogen screening (2.4); environmental parameters (2.5); data management and analysis (2.6); strategies for data dissemination and for mapping (2.7).

Chapter 3: Evaluation of the defined surveillance programme, including cost estimates (3.1) and for the planning of an evaluation of the surveillance process (3.2).

Annexes: Complementary information.

Figure 6: Procedures to be developed for the surveillance of IMS

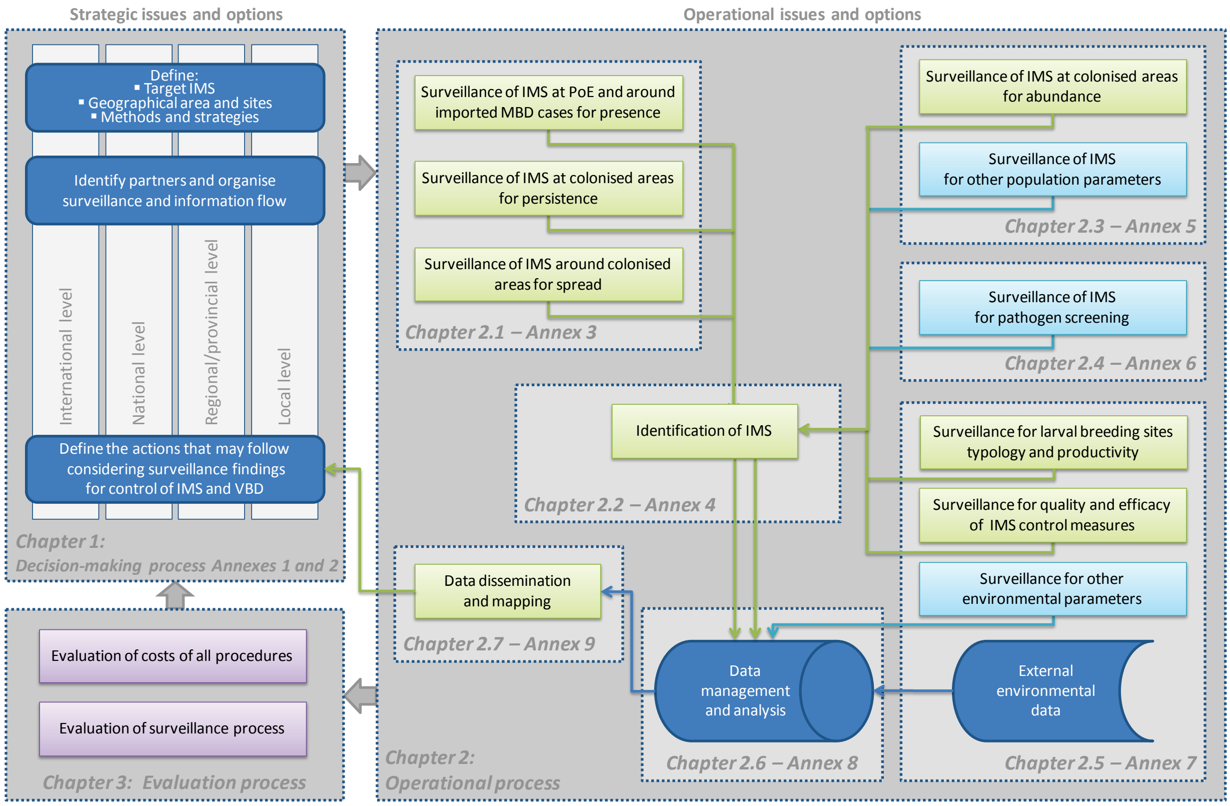

A careful evaluation of the situation will allow the definition of appropriate strategic and operational issues. Processes to be applied comprise key procedures (green rectangles) and optional procedures (blue rectangles). Procedures are listed according to the different chapters of this guideline document.

1 Surveillance of invasive mosquitoes: the decision-making process

1.1 Aim and definition of scope

The first step is to clearly define the aim and scope of the surveillance, as many subsequent choices depend on these definitions. They include (i) the objectives of the surveillance and the subsequent actions that result from the findings, (ii) the target IMS (one or more), (iii) the geographical area and the sites to be surveyed, and (iv) the methods and strategies to be implemented for each site category. Hence, surveillance of IMS should aim to implement the following:

a. Early detection of the introduction of IMS to a new territory, and survey of its possible establishment and spread

For this main objective, the surveillance network must be set up to allow the early detection of an introduction/initial spread of IMS before it establishes permanent populations and spreads over a wide area. The network should optimise resources by intelligently targeting the surveillance operations at as many previously defined high-risk sites as possible or at points of entry (PoE) (see Chapter 2.1 and Annex 2). Based on observed colonisation dynamics in Europe and other continents, specific sites at risk of introduction should be identified; surveillance measures need to be customised and proportionally tailored to the level of identified risk, based on the level of trade and travel with regions that are already colonised by IMS. The early detection of new foci of introduction during the first phase of the colonisation process, i.e. when the species is not yet well-established locally, will allow the rapid adoption of intensive control measures and thus provide the best chance of rapidly eliminating the threat.

b. Assessment of sanitary/disease risks to human health related to IMS

If the IMS is already established and widely spread (i.e. the species is found in several villages or towns) and its population is expanding, surveillance activities should support the assessment of hazards to human health, including impact of nuisance (hypersensitivity reactions to bites, mosquito phobia, etc.) and MBD transmission. All Surveillance efforts should enable the decision-making units to make quantitative estimates to support the assessment and management of sanitary and disease risk, while at the same time providing evidence for the planning of measures for both sanitary control (reducing nuisance) or disease prevention/control (reducing MBD transmission risk). If needed, activities can be extended to address risks to animal health or the possible adverse effects on biodiversity, e.g. invasive species outcompeting native mosquito species.

c. Implementation of IMS control measures and assessment of their efficacy

If the IMS is already established and widespread, the surveillance network should provide details on vector density and longevity reduction in space and time which are needed for the optimal implementation of mosquito control measures. The surveillance network should also provide all information necessary to assess the short- and medium-term efficacy of the control measures taken during and after a campaign to measure cost-effectiveness.

Box 2: Checklist – key issues of the decision-making process when planning a surveillance programme

- Identify the situation and define the aim of the surveillance to be implemented.
- Identify all partners and capacities, and define their tasks with respect to:
  - organisation and management;
  - field data collection;
  - mosquito identification;
  - data management and analysis;
  - data dissemination and mapping; and
  - pathogen screening.
- If there is a lack of qualified personnel, hold a training course.
- Organise the information flow:
  - Communication of information to stakeholders: from local units to the regional governments, to the state, to ECDC.
  - Promptly inform the central authority (contact point/person) if an IMS is detected.
  - Investigate trade connections (national and international) if an IMS is detected; inform other surveillance structures/units (national and international) about possible passive transport.
- Define subsequent control actions (directed at IMS and MBD) by analysing surveillance findings and identifying adequate partners and capacities.

1.2 Organisation and management

Four organisational levels can be conveniently identified, namely (i) international, (ii) national, (iii) regional/provincial, and (iv) local. Particular actions and tasks may best be assigned to one or more of these levels, as befits the risk and the national context (see Tables 2, 3, and Annex 2 for more detailed information and examples of organisation and management in continental Europe).

Once the aim of the surveillance is defined, the second step will be to identify potential partners and their various tasks, in order to ensure sufficient capacity. If specific capacities are missing, collaboration should be established with external providers (e.g. universities, consultancies) to ensure an adequate supply of skills; alternatively, a capacity building process has to be initiated. Responsibilities and tasks of the surveillance programme will be allocated to the different partners (see Table 3).

If risk assessment and management of threats to human or animal health is envisaged, the surveillance programme should be part of a global plan, alongside other measures (cf. Box 1 and Figure 5). In such instances, national regulations usually stipulate the nomination or identification of partners and define their responsibilities and roles. Field data sets should be incorporated into GIS applications so that spatio-temporal distribution maps of IMS (see Chapter 2.7) can be produced, thus helping decision makers in the planning of appropriate procedures for risk assessment and management.

Even though not directly related to IMS surveillance, feedback procedures should be added to the surveillance programme to assess any side effects of the control measures, i.e. impact on non-target fauna, impact on human health, and insecticide resistance management (see EMCA/WHO guidelines; cf. Figure 5).

Table 2: Actions to be taken at the different geographic levels, according to type of risk

	International	National	Regional/provincial	Local
**Risk of introduction of IMS**	Updating of regulations and recommendations aimed at reducing the risk of passive spread of IMS.	Application of International Health Regulations (IHR) to free ports and airports of vector.	Identification of critical issues and preventive measures to be discussed at (inter-)national level.	Identification of critical issues and preventive measures to be discussed at regional level.
**Risk of establishment and spread of IMS**		Development of national risk assessment and management plans for IMS and MBD:<br>• analysis and identification of introduction pathways and PoE<br>• listing and mapping of risk of PoE;<br>• defining IMS at risk for introduction and establishment;<br>• defining mosquito collection strategy and methods;<br>• defining mosquito and pathogen identification capacities;<br>• developing a national IMS and MBD control plan.	Development of regional plans for IMS and MBD surveillance:<br>• organisation and implementation of surveillance activities at sites at risk of IMS entry;<br>• collection of biological parameters of IMS;<br>• when IMS introduced, identification of origin and pathways of introduction;<br>• identification of possible spread and passive dispersal of IMS;<br>• implementing the IMS and MBD control plan if necessary.	• Providing resources and ensuring cooperation for the application of surveillance and control measures at sites at risk for IMS and MBD.<br>• Cooperation in dissemination of information to residents, to obtain community participation and avoiding local conflicts.
**Assessment of sanitary risk**				
**Management/control of sanitary risk**				

Detailed procedures for identifying sites at risk for introduction of IMS are given in Annex 3.

Table 3: Ideal allocation of responsibilities and tasks to potential partners in surveillance activities

**Ministry in charge of health (state)**	• International negotiations on regulations to prevent/limit the worldwide spread of IMS and MBD.<br>• Responsible for the application of IHR (2005).<br>• Development and coordination of risk assessment and management plans for IMS and MBD, including identification of capacities, training activities, and allotment of responsibilities.
**Ministry in charge of agriculture (state)**	Contributions to the coordination of risk assessment and management plans for zoonotic MBD.
**Ministry in charge of environment (state)**	Contributions to the coordination of risk assessment and management plans for biodiversity; assistance in the assessment of side effects of mosquito control measures.
**Public health services (state, regional/provincial, local)**	Contributions to the surveillance of IMS and MBD (i.e. mosquito pathogen screening, human cases) and to the assessment of health impact of control measures.
**Veterinarian services (state, regional/provincial, local)**	Surveillance of zoonotic MBD (i.e. animal cases among wildlife[1], livestock and pets, mosquito pathogen screening).
**Regional/provincial government (depending to the degree of autonomy)**	Definition and implementation of regional/provincial risk assessment and management plans for IMS and MBD.
**Research institutions**	• Contributions to the surveillance of IMS.<br>• Support for risk assessment/management activities, the efficacy/quality assessment of mosquito control applications and their side effects (impact on non-target fauna, impact on dispersal of mosquitoes, impact on human health), pesticide resistance management.<br>• Support for the collection of data on bionomics of IMS in specific contexts, determining spread, nuisance and vector potential. Contributions to the training of field/lab workers.
**Municipalities**	Participations for the implementation of surveillance and mosquito control measures.
**Mosquito abatement agencies (public or private)**	Management of surveillance and mosquito control applications[2].

[1] The ministry in charge of environment may contribute to wildlife surveys.

[2] Mosquito control measures could also be carried out by pest control companies if trained in mosquito control applications and subject to external efficacy/quality control.

1.3 Surveillance strategies

A framework for defining surveillance strategies for IMS, based on three probable scenarios

Surveillance methods and strategies must be customised to the various local situations, which in turn determine the strategies and methods to be implemented. The following paragraphs describe three likely scenarios that may be encountered. The risk estimate given in these scenarios is based on presence and abundance of mosquitoes, and not the likelihood of transmission of MBD such as dengue and chikungunya. If the country already faces an outbreak of MBD, then surveillance activities may have to be extended and/or strengthened according to a set of complementary guidelines for the surveillance and control of vectors and MBD (cf. Figure 5).

Surveillance plans can be implemented over a range of geographic scales, from country, region/province to smaller localities. Optimal organisation will, however, require centralised implementation (national or regional/provincial) for efficient and rapid customisation or, if necessary, extension of measures.

Box 3: Summaries of the three scenarios used for the development of surveillance strategies

Scenario 1: No established IMS

There is a risk of introduction and establishment of IMS but it has not been reported yet.
Surveillance aims at detecting possible introduction and establishment of IMS at PoE.

- Surveillance is ready to be implemented around MBD cases.
- IMS elimination plan is defined and ready to be activated.
- Information flow is defined and ready to be activated.

Scenario 2: Locally established IMS

An IMS population is locally established in a small area, with no evidence of spreading.
Surveillance aims at quantifying establishment and detecting possible spread of IMS.

- Surveillance is still performed at PoE and ready to be implemented around MBD cases.
- Surveillance of colonised locations is intensified and carried out in surrounding areas.
- Mosquito control plans are activated for all areas and events where/when IMS are reported.
- Surveillance of IMS populations is performed to assess efficiency of control measures.
- Communication plan is activated.

Scenario 3: Widely established IMS

At least one IMS population has colonised a large area by local spreading.
Surveillance aims at assessing IMS population dynamics.

- Surveillance is still performed at PoE and ready to be implemented around MBD cases.
- Surveillance is maintained at colonised area to assess abundance and seasonal dynamics.
- Surveillance is extended to the surroundings for detecting spread.
- Mosquito control plans are activated for all areas where/when IMS are reported.
- Surveillance of IMS population is performed to assess quality/efficiency of control measures.
- During outbreak, surveillance is performed to detect pathogens in field-collected mosquitoes.
- Communication plan is activated.

Scenario 1: No established IMS (but with risk of introduction and establishment)

- There are no indications of established IMS and no reports of IMS exist; alternatively, initial findings of IMS could not be confirmed over the course of months or years.
- There is a risk of introduction of IMS to a region or country related to the levels of:
  - commercial trade of goods associated with transport of IMS (primarily used tyres);
  - ground traffic originating in colonised areas within 600 km; and
  - air traffic originating in any colonised and MBD-endemic/epidemic areas.
- There is a risk of establishment and spread of IMS if models show that climatic and environmental factors match the mosquito's specific ecological requirements. In this scenario, IMS could be reported repeatedly without becoming established (over the course of at least two seasons), possibly due to unfavourable climatic or environmental conditions, or to immediate application of efficient mosquito control measures.

Scenario 2: Locally established IMS (with low risk of spreading into new areas)

- Locally established IMS indicates a situation where the species is still at the very beginning of the colonisation phase; the population remains restricted to a small area with an indicative maximum area of 25 km² (i.e. a road service area at or nearby a country border crossing, a port, a village).

- The absence of evidence for spreading may indicate that the IMS population is not (yet) adapted to the local conditions or has not reached the density needed to spread further; thus, the situation can still be considered low risk.

Scenario 3: Widely established IMS (with high risk of spreading into new areas)

- Widely established IMS populations indicate a situation in which the species has already colonised at least several patches or villages, or a large area/town, with an area of more than 25 km². This indicates that the local spread has already started and that the IMS population is large enough to pose a high risk of further spread.

For each scenario, a series of key surveillance measures should be implemented (see Box 4). Scenario 2 includes measures listed for Scenario 1, and Scenario 3 includes all measures listed for Scenarios 1 and 2. Thus, for example, a region facing expansion of one IMS must still survey PoE for other IMS. Small countries will face only one scenario, whereas large countries can simultaneously be presented with different scenarios in different provinces. Optional surveillance measures can also be implemented to get complementary information. Key and optional surveillance measures will be described in later sections (Chapter 2 and Annex 3). More detailed information about the aims of surveillance according to each scenario is given in Annex 2.

Box 4: Checklists of surveillance measures for three scenarios used for the development of surveillance Strategies

Checklist 1, for Scenario 1: no established IMS

- Active surveillance of possible introductions at PoE (used tyre and Lucky Bamboo trade, airports, ports, major ground transportation routes, etc.).

If autochthonous MBD cases are reported which are suspected to be transmitted by IMS:

- Domestic reports: active surveillance in areas reporting cases, including neighbouring areas.
- From neighbouring countries: active surveillance to be intensified.
- Information procedure (to central/local authorities) defined and ready to be activated if IMS detected.
- Mosquito elimination plan defined and ready to be activated if IMS detected.

Checklist 2, for Scenario 2: locally established IMS

- Active surveillance of the colonised area to assess the persistence of the IMS population.
- Active surveillance of the surroundings of the colonised area to determine the possible extent and the routes of spread of the IMS.
- Investigation of the IMS introduction pathway and attempted identification of its origin.
- Information transfer procedures activated: rapidly inform central/local authorities about findings and sanitary risks; inform international authorities to alert other regions/countries at risk.
- Mosquito elimination plan activated: implementation of control measures at all sites and episodes where/when IMS are reported from a perspective of elimination of new foci.
- Active surveillance of IMS populations to assess quality/efficacy of control measures.

Checklist 3, for Scenario 3: widely established IMS

- Active surveillance of the colonised area to assess the abundance of the IMS population and its seasonal dynamics.
- Information transfer procedures intensified for MBD risk plan definition and implementation.
- Mosquito elimination plan extended: implementation of control measures at colonised areas from a perspective of controlling nuisance and MBD transmission risk.

Checklist 4, optional surveillance measures for all scenarios

- Pathogens screening plan defined and ready to be activated, in case of (1) pathogen (relevant for IMS, e.g. dengue and chikungunya) introduction/circulation in the area, (2) direct IMS importation from a region endemic for such MDB, and (3) report of MBD cases possibly related to IMS in the area (see Chapter 2.4).
- Environmental parameters for IMS populations (see Chapter 2.5).

Checklist 5, optional surveillance measures for Scenario 3

- Other population parameters for established IMS populations (see Chapter 2.3).

2 Operational process: The surveillance of invasive mosquitoes

2.1 Methods of collection of invasive mosquitoes

Figure 7: Diagram of IMS surveillance procedures, according to the identified risk and Scenarios 1 through 3

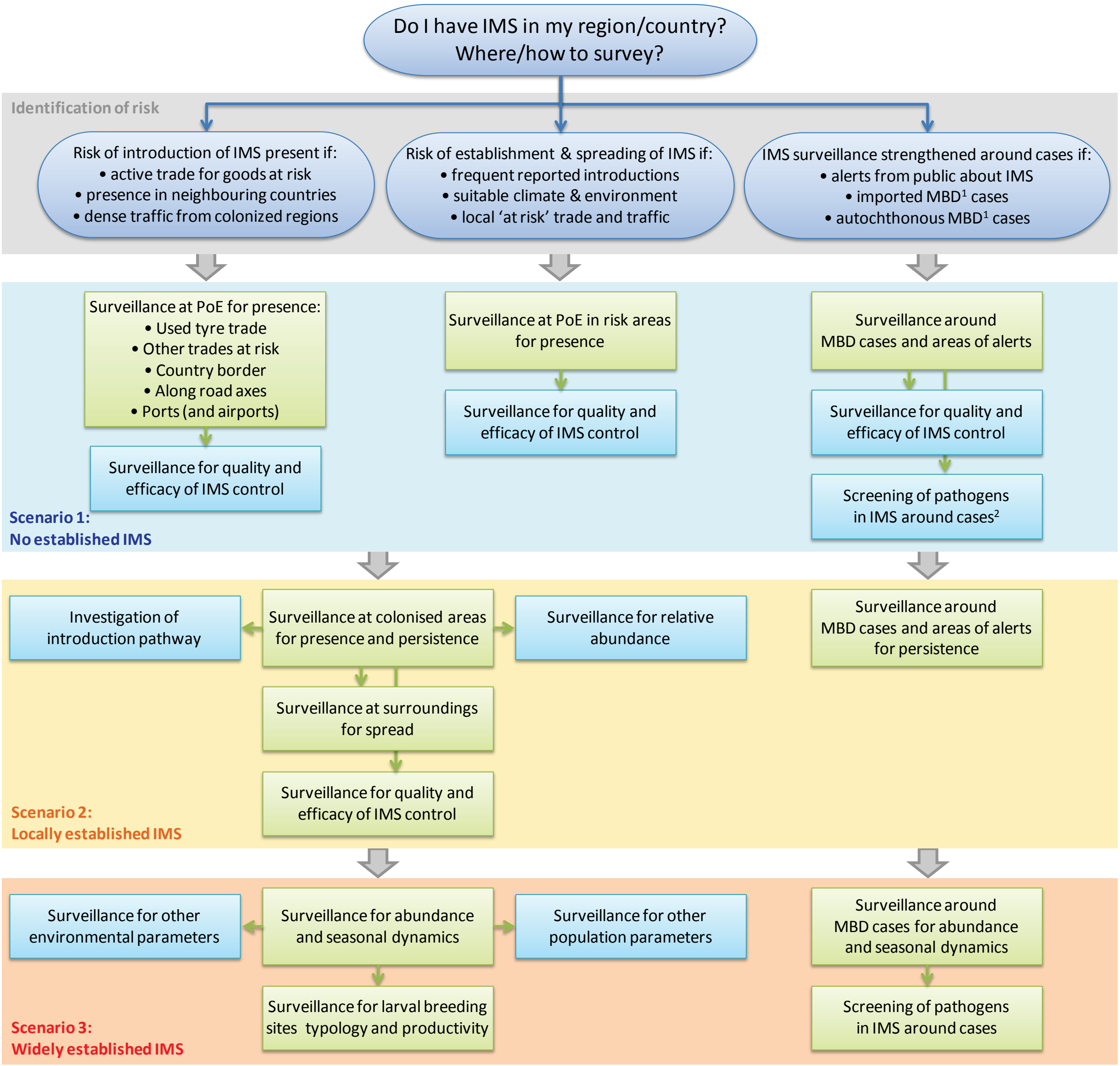

Key procedures (green rectangles) and optional procedures (blue rectangles) should be implemented according to the estimated risk and information gathered during the preparation phase. The process must be adapted in real time and take into account surveillance results as well as additional external information. Procedures listed for low-risk scenarios should also be included in the higher risk scenario(s). An optional procedure in a low-risk scenario may become a key procedure in a higher risk one.

[1] *Suspected to be transmitted by IMS*

[2] *Can also be performed on imported IMS at PoE.*

Mosquitoes can be collected at different stages of their life cycle: as larvae, adults or eggs (see information on the mosquitoes' life cycle and biology in Annex 1). Mosquito surveillance/collection strategies should be determined by the specific environmental characteristics of the surveillance sites (see Chapter 2.5) and the most probable species at risk of introduction, as inferred by previous analysis (cf. Chapter 1). Logistic and cost factors may also affect the choice of surveillance methods to be used.

Surveillance of larval (juvenile) stages is of key relevance for detecting IMS, allowing targeted and rapid sampling efforts and an optimal cost–benefit ratio. As the IMS considered in this guidance document are largely container-breeding species, therefore larval sampling in urbanised areas must focus on containers, both man-made and natural, rather than other stagnant bodies of water (ditches and ponds with vegetation, fen, flooded meadows or forest) as these are never colonised by the species being considered. Mosquito larvae can be collected by surveying techniques such as netting, dipping, and aspiration, depending on the size of sampled container.

Adult mosquito females can be caught by direct aspiration from resting sites or hosts and by netting from the vegetation. Human landing collection (HLC) can also be performed if necessary, provided the ethical issues involved are fully taken into account. Adult surveillance is, however, best carried out using traps which attract the insects. For IMS, the most efficient traps are suction traps baited with chemical lures or/and with carbon dioxide, and those which attract ovipositing females. Trap type choice depends on the most probable mosquito species to be found, the environmental conditions at the selected trap sites, and the availability of resources (skilled technicians, hosts, dry ice, power supply, etc.).

Surveillance of IMS adult presence and activity can also efficiently be based on so-called ‘ovitraps’ that attract adults which lay their eggs in the traps. However, as only eggs are collected, only indirect data on adult populations are obtained. Detailed information on collection/trapping methods is given in Annex 3.

Choice of the method of collection

Recommendations for the choice of IMS collection methods according to surveillance strategies and procedures, with indications of required trap density, frequency and sample period are given in Table 4. Table 5 provides reporting schemes for the recording of results from the most common types of mosquito traps. Both tables should be considered together in order to select/adapt the appropriate trapping strategy according to (1) the aim of the surveillance and its expected outcomes and (2) the targeted IMS because the effect of species on trapping efficiency is not well understood and may vary considerably. Surveillance strategies should also take a number of additional factors related to the local context and constraints into account e.g. survey area size, site accessibility, and the numbers of traps needed for each type of environment present.

A number of methods are available to collect IMS population parameters (see Annex 5, Table A). Adult trapping for pathogen screening is best performed using gravid traps that attract egg-laying females (which have already taken a blood meal and thus may be infected), in combination with CO_2-baited traps to enlarge the trapping radius. Detection of IMS at PoE should be performed with a wide spectrum of traps and methods (including larval search and HLC). By contrast, long-term surveillance may be based on cheap and easy-to-check traps (e.g. ovitraps) and/or on traps that can run permanently or for several days, which limits the need for trips to service the traps (e.g. BG-Sentinel or Mosquito Magnet (MM) traps). These are, however, relatively expensive to operate, but whilst CO_2-baited suction traps may be cheaper, they are usually battery operated, and the endurance of the CO_2 supply is limited. Additional factors determining trap choice are the combination of parameters required from a single trapping programme, the availability of material already acquired, and the range of funding possibilities. Surveillance costs can be reduced if the traps are run by local operatives who send the samples to a central processing centre.

Table 4: Recommended mosquito collection methods according to aims of IMS surveillance, with indications of density, frequency and period of the year

Scenario 1	Scenario 2	Scenario 3	Surveillance aim and sites	Methods and traps	Density of traps	Frequency of trapping	Period of trapping
			**Introduction at points of entry**				
✓	✓	✓	Storage sites for imported used tyres	• BG-Sentinel or MM<br>• HLC<br>• Larval search	• 1/5000m²<br>• 1 or 2<br>• 1/10 tyres	Twice a month<br>Twice yearly<br>Twice-yearly	Apr–Nov<br>Jul–Nov<br>Jul–Nov
✓	✓	✓	Shelters/greenhouses for imported plants like *Dracaena* spp. (Lucky Bamboo)[1]	• BG-Sentinel or MM<br>• HLC<br>• Larval search	• 1/5000m²<br>• 1 or 2<br>• 20 vessels	Twice a month<br>Twice yearly<br>Twice yearly	Jan–Dec<br>Jan–Dec<br>Jan–Dec
✓	✓	✓	Main parking lots at country borders, highways and road axes that originate in colonised areas[2]	• Ovitraps<br>• HLC<br>• Larval search	• 1/2500m²<br>• 1 or 2<br>• 10 vessels	Twice a month<br>Twice yearly<br>Twice yearly	Apr–Nov<br>Apr–Nov<br>Apr–Nov
✓	✓	✓	Ports[3]	Ovitraps	1/5000m²	Twice a month	Apr–Nov
(✓)	(✓)	(✓)	Airports	• Ovitraps<br>• BG-Sentinel or MM	• 1/1 ha<br>• 1/2.5 ha	Monthly<br>Twice a month	Apr–Nov<br>Apr–Nov

Scenario 1	Scenario 2	Scenario 3	Surveillance aim and sites	Methods and traps	Density of traps	Frequency of trapping	Period of trapping
(✓)	✓	✓	Quality and efficacy of control measures	• Ovitraps<br>• BG-Sentinel, HLC	• 20/site<br>• 4/site	Before and after applications	
			**Persistence in colonised area**				
	✓		Inspection of colonised area	• Ovitraps (BG-Sentinel, MM)<br>• HLC<br>• Larval search	• 1/5 ha<br>• 1/20 ha<br>• 3 or 4<br>• 40 vessels	Twice a month<br>Twice a month<br>Twice a month<br>Twice a month	Apr–Nov<br>Apr–Nov<br>Apr–Nov<br>Apr–Nov
	(✓)	✓	Abundance and seasonal dynamics	• Ovitraps (BG-Sentinel<br>or<br>• CO_2-baited traps or gravid traps)	• 6/site<br>or<br>• 2/site	Twice a month<br>Twice a month	Jan–Dec[4]<br>Jan–Dec[4]
		(✓)	Other mosquito population parameters (e.g. biting behaviour)	• Baited traps<br>• HLC<br>• Aspirators	2/site	Monthly	Jun–Sep
		✓	Infection of IMS by pathogens	• Gravid traps<br>• BG-Sentinel	2/site	Weekly	During and after outbreaks
	✓	✓	Quality and efficacy of control measures	• Ovitraps<br>• BG-Sentinel<br>• HLC	• 20/site<br>• 4/site	Before and after applications	
			**Spread into areas surrounding colonised areas**				
	✓	✓	Inspections around colonised areas	Ovitraps	1/15 ha	Monthly	Apr–Nov
		✓	Quality and efficacy of control measures	• Ovitraps<br>• BG-Sentinel<br>• HLC	• 20/site<br>• 4/site	Before and after applications	

According to the scenario, procedures are listed as key (in bold) or optional (in brackets); several complementary sampling/trapping methods can be performed simultaneously (e.g. ovitraps and adult traps); required densities and frequencies are indicative averages which must be adapted to the risk level and the size of the area; ovitraps are run permanently over the period, other traps for 24h, 48h or one week, depending to the available power source; HLC can be performed when visiting the trap sites; trapping periods are given for central Europe and must be adapted to local climate.

[1] Storage sites for goods or equipment able to retain rain water could also be surveyed (e.g. sites with stone fountains, junkyards with damaged vehicles or equipment).
[2] Includes parking lots at commercial centres close to country borders.
[3] Ports with international trade; tourist ferry ports and marinas with colonised areas.
[4] Required during the first year; can later be limited to the period of development in the local climate.

Table 5: Methods of collection of IMS adult or detection by egg collection and their efficacy according to mosquito species

Targeted species	Host-seeking females					Oviposition-seeking females		
Trap models	HLC	CO_2 traps	MM (CO_2)	Light traps	BG-Sentinel	Gravid traps	Sticky traps	Ovitraps
Ae. aegypti	+++	+/-	+	-	++	+/-	++	++
Ae. albopictus	+++	+/-	+	-	++	+/-	++	++
Ae. atropalpus	++	+	+	-	+/-	-	?	+
Ae. japonicus	+	+/-	+	+	+/-	++	+	+/-
Ae. koreicus	?	?	?	?	?	-	?	+
Ae. triseriatus	+++	++	++	?	++	+	+	++

HLC = human landing collection; CO_2 traps = CO_2-baited suction traps; MM = MosquitoMagnet, CO_2-baited suction traps with chemical attractant; light traps = light-baited suction traps; BG-Sentinel or Mosquitaire = odour-baited suction traps; gravid traps = infusion-baited suction traps; sticky traps = water/infusion-baited oviposition trap with sticky element; ovitraps = water/infusion-baited oviposition traps (only eggs are collected); - low efficacy; + fair efficacy in some situations; ++ good efficacy; +++ excellent performance; ? not known

Complementary information is given in Annex 3, including strengths and weaknesses of the methods for collecting adult IMS or detecting their activity by collecting eggs, examples of trap positioning on field maps, illustrations of trap models, and links to complementary information.

2.2 Methods of identification of invasive mosquitoes

Methods for collection, handling and storage of mosquito samples for identification

Mosquito **larvae** are collected from larval breeding sites, by netting, dipping, or aspiration (see Chapter 2.1 and Annex 3). In all cases the collected material should be inspected in a white plastic tray (1 litre) filled with clear water from the site and the juveniles transferred with a plastic pipette into a fully labelled vial filled with water for subsequent rearing to L4 or adults. For long-term storage they can be kept in airtight tubes filled with 70% ethanol. If frequent inspection is likely, they can be mounted on slides.

Pupae can be collected and stored in the same way as the larvae. However, it is recommended to keep them alive after collection (in water from the collection site) and to rear them to the adult stage in the laboratory. Pupae often drown during transport, but survival rates can be increased if there are regular stops to allow pupae to suck in air.

Adult females can be caught by aspiration from resting sites (indoors in shelters, or outdoors from the vegetation) or from hosts (animal or human), and by netting amongst the vegetation. However, routine surveillance of IMS is more effectively performed with traps (for choice of methods see Chapter 2.1 and Annex 3). They can be stored dry (in vials with silica gel and cotton to immobilise the sample and prevent damage to legs and wings) or frozen. For long-term preservation or identification they must be pinned, labelled and stored in an insect collection box.

Adult males are rarely collected, as they are not usually attracted by common trapping techniques, but can be attracted by hosts or mosquito females. Thus, males are usually caught by netting or aspiration from resting sites in the vegetation. Specimens in good condition (both male and female) can be obtained by rearing juveniles (see Annex 4B). Males should be stored in 70% ethanol but not silica gel as desiccation will damage the genitalia. For long-term preservation or identification they must be mounted on slides (at least the genitalia) and pinned, labelled and stored in an insect collection box like females.

Eggs of invasive *Aedes* spp. on tape paper or oviposition supports (cf. Chapter 2.1) can be stored on their supports in a closed plastic bag, at room temperature or in a fridge (temperature range 5 to 15° C). The bag should not contain any free water, but should still be humid (humidity rate around 55%).

Identification of invasive *Aedes* mosquito species

A list of major available identification tools is given in Annex 4C.

Larvae can be identified by their morphology at almost all instars (L1 to L4). However, descriptions and identification keys are usually based on L4 or on the exuvia shed during pupation. The most frequently used morphological characters are setae and other features (presence/absence, number of branches, shape) located on the head and on the abdominal terminal segments. Some characters may vary from one instar to the other (e.g. number of branches of a seta) whereas others are more consistent (e.g. presence/absence of a seta). Identification can usually be performed under a binocular microscope, but some characters may need higher magnifications and therefore require inspection of slide-mounted specimens using a more powerful microscope (Figure 8c). Specimens can also be identified by molecular tools (see Annex 4F). An identification key for larvae of mosquito species of Europe, breeding in containers, is given in Annex 4D.

The identification of **pupae** is possible to genus level but infrequently to species level, as there are generally few taxonomic diagnostic characters; no pupal identification key exists for pupae and complete descriptions are rare. It is therefore recommended to collect them live and to rear them to obtain fresh adults for identification (See Annex 4B). Well-developed male pupae can, however, be dissected and the genitalia used to identify them in the same way as for adult males. Like larvae, pupae specimens can also be identified by molecular tools (see Annex 4F).

Adults have first to be sorted by gender (see Annex 4A). **Adult females** can be identified to species level by morphology using a binocular microscope. The main diagnostic features are coloration pattern of legs, abdomen, and thorax, produced by patches of coloured scales (see Figures 8a-b, 9). Fresh specimens are easier to identify. When old females are caught or trapped, scales are often missing and identification using coloration becomes more difficult. Thus, identification by molecular tools will be very helpful (see Annex 4F). An identification key for adult females of mosquito species in Europe breeding in containers is given in Annex 4D. **Adult males** can be identified similarly. Most of the morphological characters used for females are also valid for males (see Figure 9). Identification can also be made solely with slide-mounted genitalia (Figure 8c), based on the shape of some organs and their features. Molecular tools can be also be used for the identification (see Annex 4F) of females and juveniles.

Identifying the **eggs** of invasive mosquitoes can be challenging. Eggs can be recognised by their general shape (Figure 8d), their size, and the structure of the outer chorion (egg shell). Not all species, however, have their eggs described, and identification keys only exist for a few species. It is usually easiest to let the larvae hatch from the eggs and identify the resulting larvae or adults (see Annex 4B). Egg identification requires laboratory equipment. For identifying or sorting eggs before the larvae hatch, a classic microscope or binocular can be used (100x

magnification is ideal). For identifying by checking the sculptures on the outer chorion surface, a microscope with a reflecting (or episcopic) lightening is needed (400x magnification). Eggs can be also be identified with molecular methods (see Annex 4F). References for description of eggs of invasive and indigenous European container-breeding *Aedes* species are given in Annex 4E.

Figure 8: Conservation of collected mosquitoes

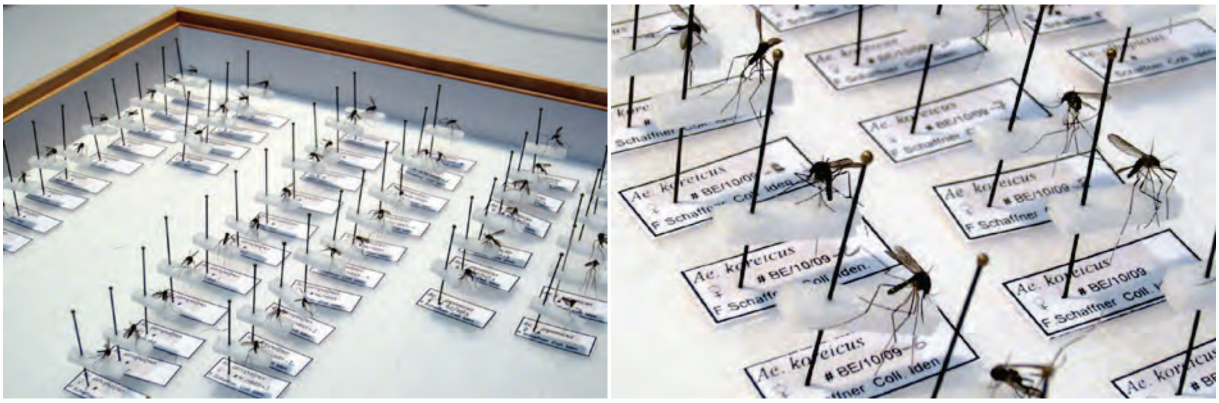

Above: pinned adults

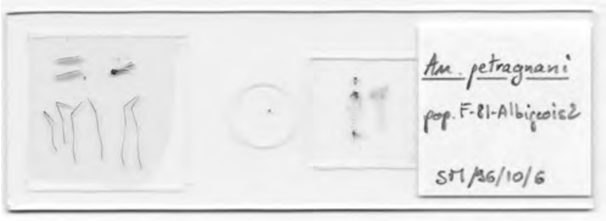

Above: Mounted individual (left to right: parts of adult, male genitalia, larvae and pupal exuviae)

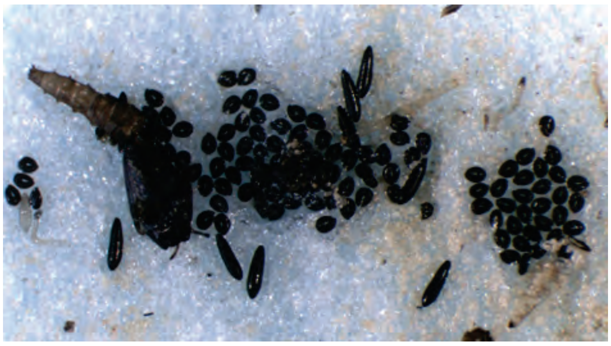

Above: Eggs on polystyrene from an ovitrap (oblong black eggs: Ae. albopictus*; rounded eggs:* Psychodidae*)*

Photos: F. Schaffner

Finding a species new to a country / region

As a rule, when finding a new species by using identification keys, a complete description of the species should be checked and compared with descriptions of other closely related species that may be present. Specimens may also be compared with voucher specimens of the same species if available. If additional confirmation is needed, or if specimens are unidentifiable by visual examination, molecular identification can be performed (see Annex 4F). Such identifications should also be confirmed by an acknowledged expert in mosquito taxonomy before it is formally reported. In addition, experts should be asked to validate the entire sampling and collection procedure and to verify the identification process on a randomly chosen sub-sample.

Box 5: Key issues and recommendations for the identification of IMS

Larvae

- Inspection in a white plastic tray with clear water from the site and transfer to a labelled vial with water or directly in 70% ethanol; later kept in 70% ethanol or mounted on slides.
- Identification keys are usually based on L4 stage, which is easier to identify (or on L4 exuviae after pupation).

Pupae

- Stored like the larvae; see above.
- No identification key available; should be reared to the adult stage.

Adults

- Specimens can be stored dry, frozen, or pinned in an insect collection box.
- Gender identification through rapid check of the head (shape of palpi and antennae).
- Females can often be easily identified to species level by morphology (fresh specimens) by using identification keys; males can also be identified from slide-mounted genitalia.

Eggs

- Storage on their support (tape paper/oviposition support) in a closed plastic bag (temperature range 5° C to 15° C; humidity rate 55%)
- Species identification is challenging; it is easier to let the eggs hatch and identify the obtained larvae or adults.

Overall

- In certain cases or areas, IMS larvae should be reared to adults to allow accurate species identification.
- Molecular tools are also available to identify mosquitoes at any stage of the life cycle.

Figure 9: Main diagnostic morphological characters for adults of IMS

Figure 9: Main diagnostic morphological characters for adults of IMS. A. Thorax (scutum, dorsal side); B. Abdomen (dorsal side); C. Hind tarsus (last segments of the third leg). Males have a more hairy and slender abdomen. A. Thorax (scutum, dorsal side); B. Abdomen (dorsal side); C. Hind tarsus (last segments of the third leg).
Males have a more hairy and slender abdomen

Ae. aegypti	*Ae. albopictus*	*Ae. atropalpus*	*Ae. j. japonicus*	*Ae. koreicus*	*Ae. triseriatus*
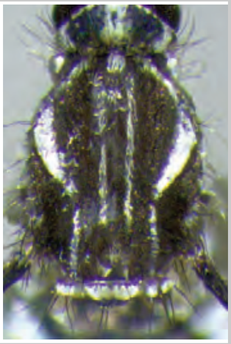	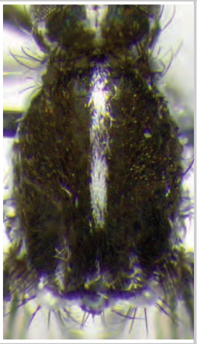	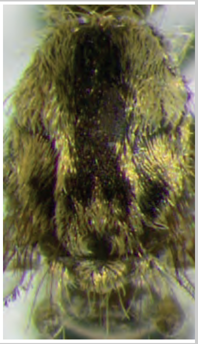	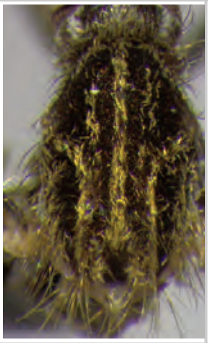	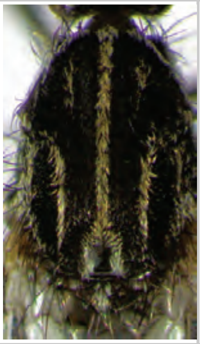	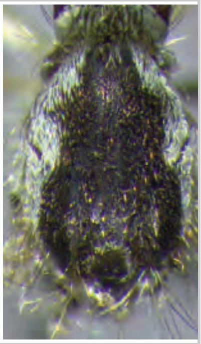
A. Silver scales in a shape of a lyre	A. Median silver-scale line	A. Mixed yellow and dark scales, dark median stripe	A. 5 stripes of golden scales, submedians long	A. 5 stripes of golden scales, submedians short	A. Broad median dark-scale band, sides with silver scales
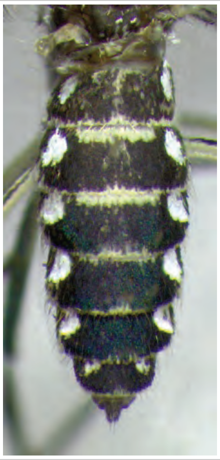	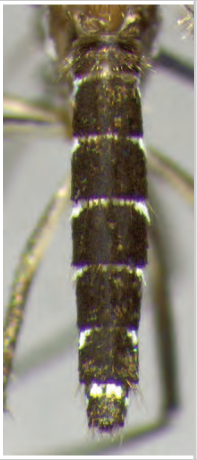	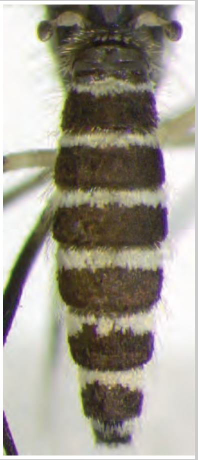	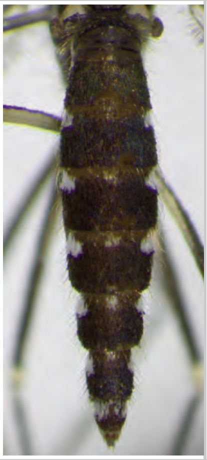	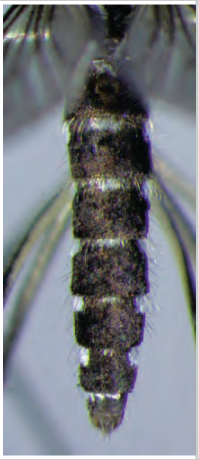	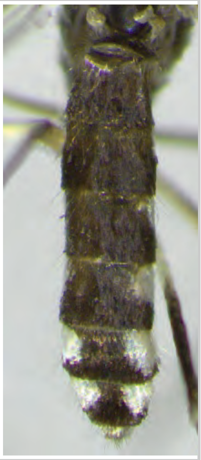
B. Pale lateral marks and thin basal bands	B. Thin basal pale bands, laterally enlarged	B. Pale basal bands	B. Pale lateral and median patches	B. Pale lateral and median patches	B. Pale lateral patches
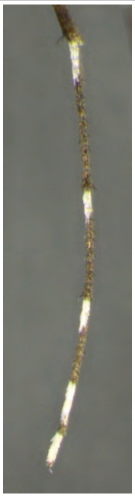	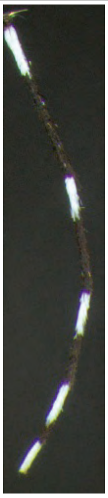	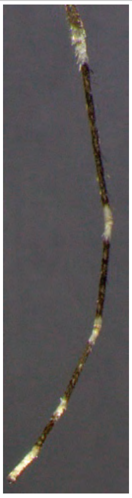	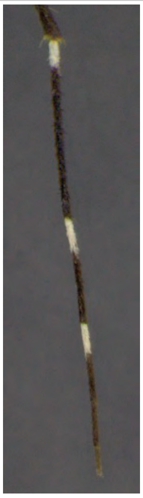	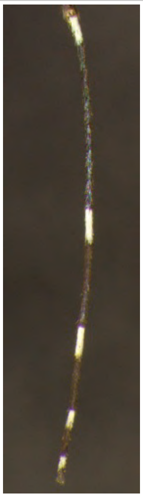	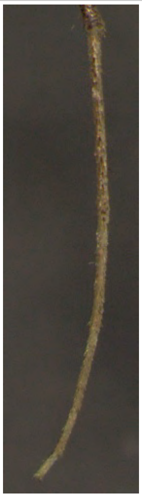
C. Segment V entirely white	C. Segment V entirely white	C. Inter-articular pale bands S. V white	C. Segments IV & V black	C. Segments IV and V with basal pale bands	C. Tarsi entirely dark

2.3 Procedures for the determination of mosquito population parameters

Figure 10: Surveillance procedures for IMS population parameters and their main data sets according to expected outcomes and Scenarios 2 and 3

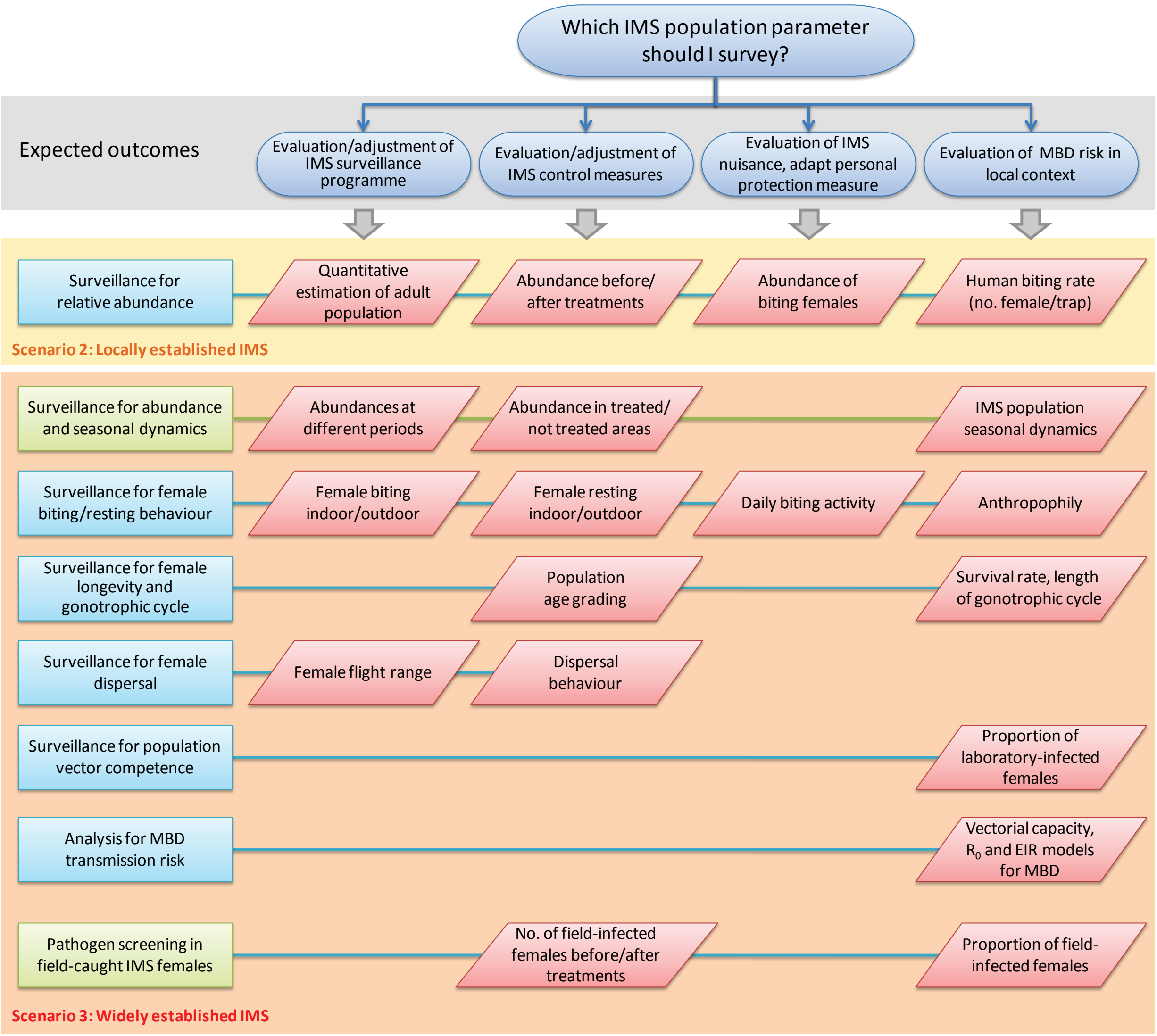

Data sets (red parallelograms) – obtained from key procedures (green rectangles) and optional procedures (blue rectangles) – may be collected according to the required outputs. Surveillance of population parameters is not suggested for Scenario 1. Pathogen screening is treated in Chapter 2.4. Procedures listed for Scenario 2 must also be part of Scenario 3.

If IMS are established over wide areas, standardised IMS surveillance should be performed to assess not only species distributions but also the mosquito population and life-history parameters (cf. Chapter 1.3, Scenario 3). This information will enable the vectorial capacity of the IMS population to be evaluated in the local context, which helps to improve risk assessments for local MBD transmission and thus allows the efficient planning and optimisation of nuisance/vector control operations. Such surveillance can be performed during outbreaks or as a preventive measure, even before there is any evidence of local MBD transmission.

Population estimates of mosquitoes usually describe a wide range of species-specific developmental life-history parameters that are affected by the characteristics of the new environment. They include: abundance, longevity (including the length of the four life stages – egg, larva, pupa, adult – and their survival rates), capacity for dispersal, host preference, the number of gonotrophic cycles (duration of time between two ovipositions) in a mosquito's lifetime and their length, fertility, fecundity, mortality, the intrinsic rate of increase, the net reproductive (replacement) rate, birth rate, death rate, and generation time. These parameters are all mosquito-borne, i.e. parameter values are first and foremost dependent on the mosquito species, which adapts to its new environment, whereas environmental parameters (see Chapter 2.5) are first and foremost determined by the environmental and climatic conditions which are more or less favourable to the IMS. The key population parameters to be considered

are: (1) population abundance and dynamics during the season, (2) female longevity, (3) female biting behaviour, and (4) dispersal capacity. These key parameters combined with vector competence may help to determine the vectorial capacity and basic disease reproduction number (R_0) to provide a basis for MBD risk assessment (see Box 7). Table 6 summarises the strengths and weaknesses of mosquito population key parameters as well as procedures and methods for estimating vector competence and procedures for estimating transmission. For this last procedure, the vector component will be a combination of several population parameters within an equation (i.e. vector competence or R_0) indicating the ability of a mosquito population to maintain MBD transmission (Box 7).

Complementary information is given in Annex 5, including detailed methods, tools, indices and formulas for collecting data for these key parameters. Main key parameter values for each IMS, together with their methods of acquisition, can be found in the VBORNET IMS fact sheets.

When planning IMS surveillance, stakeholders should contact research institutions dealing with IMS and request support in the preparation and implementation phases of Scenarios 1 and 2. If IMS are widely established (Scenario 3), ministries responsible for human and animal health, agriculture and environment should promote the involvement of interdisciplinary research teams (medical entomology, public health, meteorology, GIS, etc.) to investigate population parameters of the locally established IMS populations in order to accurately estimate the risk of MBD transmission. Enlisting such resources would also be helpful in combating outbreaks through a tailored package of IMS control measures.

Box 6: Key issues and recommendations for mosquito population parameters

- The main parameters are (1) abundance, (2) longevity, (3) biting behaviour, and (4) dispersal. Estimates of these parameters are available in the literature. However, it is recommended to also estimate them for the local mosquito population as parameters might vary according to the population and are influenced by environmental factors.
- These parameters are essential in determining the epidemiological status of the vector population, assessing the risk of MBD transmission, and developing/evaluating effective control programmes.

Table 6: Main characteristics of key parameters for mosquito populations

Parameters	Information provided	Strengths	Weaknesses	Data collection methods and equipment
**Population abundance**	Quantitative estimation of IMS adult population; seasonal dynamics; comparative analysis throughout the years; nuisance and MBD risk assessment	Supports the evaluation of nuisance threshold definition, specific risk assessment and control efforts	Good organisation and quality control required	• Breeding site, ovitrap, pupal or adult surveys<br>• Adequate field material
**Female longevity, gonotrophic cycle and dispersal**	Life traits key parameters required to evaluate MBD risk	Valuable data to feed epidemiological equation	High-tech laboratory required; large spatio-temporal variability; needs replications; expensive	• Mark-release-recapture, laboratory experiments<br>• Rearing facilities and specialist equipment
**Female biting behaviour**	Life traits key parameters required to evaluate MBD risk; nuisance protection; nuisance threshold	Valuable data to feed epidemiological equation; information for citizens	High-tech laboratory required; extensive field work; expensive	• Field and laboratory experiment<br>• Traps and laboratory equipment
**Population vector competence**	Life trait key parameters required to evaluate MBD risk for main pathogens	Essential data to feed epidemiological equation	Needs BL3 laboratory; expensive	• Laboratory infections<br>• BL3 rearing facilities and equipment
**Transmission risk**	Synthetic estimation of the MBD risk for main pathogens in local context and throughout the season	Evidence-based support for PH policies	Required: skilled entomologists, epidemiologists; statistics, high-tech laboratory; expensive	• Gathering field and laboratory data<br>• Analysis, modelling, mapping equipment

Box 7: Introduction to MBD transmission models

Many transmission models have been developed for MBD (i.e. primarily for malaria), for example human blood index (HBI), entomological inoculation rate (EIR), vectorial capacity (C), and human biting rate (HBR). The complexity of the vector-borne disease cycles implies that mathematical models describing these cycles have numerous parameters. A discussion of the basic reproductive number for MBD and its implications for MBD control can be found in Smith & McKenzie 2007.

The commonly used basic reproduction number (sometimes incorrectly called 'basic reproductive rate' because it does not consider units of time), R_0, is defined as the expected number of secondary cases produced by a single (typical) infection in a completely susceptible population. It is the multiplication outcome of transmissibility (i.e. probability of infective contact between an infected and a susceptible individual), the average rate of contact between infected and susceptible individuals, and the duration of infectiousness (Hethcote 2000; Heffernan et al. 2005).

When $R_0 < 1$, each infected individual produces, on average, less than one new infected individual, and therefore the infection will eventually disappear from the population. If $R_0 > 1$, the number of infections will increase and the disease will spread further within the susceptible population.

Vectorial capacity (a mosquito parameter component of R_0) is a measure which is essentially independent of the prevalence of pathogen infection. It represents the transmission potential of a local mosquito population and is very similar to R_0 because it represents the expected number of humans infected per infected human per day (assuming perfect transmission) in a completely susceptible human population. Adding transmissibility and the duration of infectiousness produces a measure directly analogous to R_0. (See Annex 5 for references.)

2.4 Procedures for mosquito pathogen screening and identification

Figure 11: Decision tree for screening of pathogens (virus/parasite) in IMS, showing procedures, data sets and their expected outcomes, according to Scenarios 1 through 3

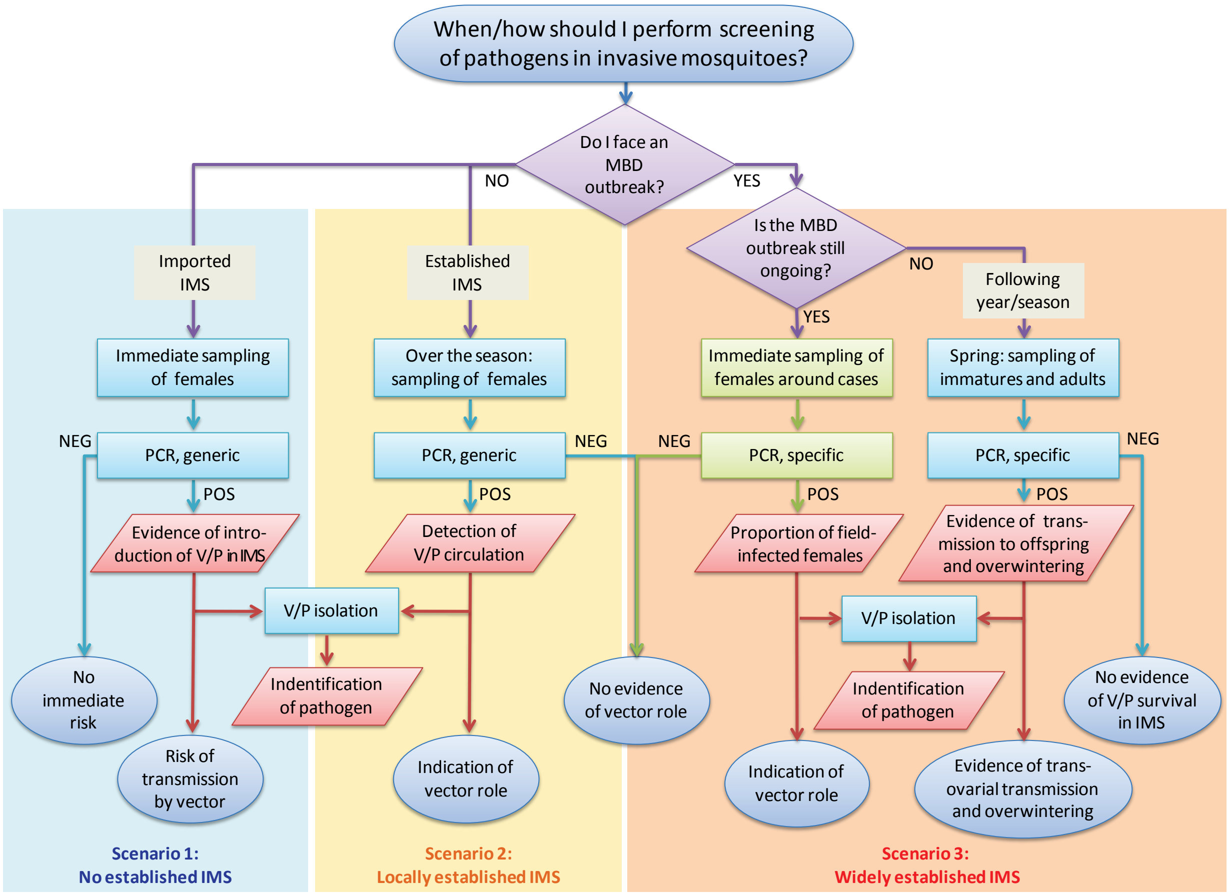

Data sets (red parallelograms), obtained from key procedures (green rectangles) and optional procedures (blue rectangles) may be collected according to the required outputs.

Why and when should pathogens in mosquitoes be screened?

As suggested in Scenarios 1 through 3 (cf. Chapter 1.3), IMS can be collected for the screening of pathogens.

Risk assessment procedures for transmission of the MBD should be immediately implemented if there is an MBD outbreak; if the presence of an MBD pathogen is reported in, or close, to an area under surveillance; or if a significant risk of MBD pathogen introduction exists. If preventative measures are already in place, they should be strengthened. Risk management decisions (including vector control measures) can be restrictive and costly, and therefore need to be based on reliable evidence obtained from a risk assessment programme. If IMS are established over wide areas (Scenario 3) they should be surveyed to assess the abundance of the population and its seasonal dynamics (throughout the year). These data, together with other mosquito population parameters (e.g. host preferences), will help to evaluate the vectorial capacity of the IMS population in the local context (cf. Chapter 2.3).

The risk assessment process is lengthy and complex, and reliable indicators may be obtained faster by active screening of pathogens in mosquito females initially collected around reported MBD cases. Such screening provides limited information, as (1) negative results do not prove the absence of pathogen circulation in the considered species, and (2) positive results do not necessarily prove that the species is an active vector. But if, in addition, efficient vector competence can be confirmed from scientific literature, then it is likely that the species is involved in the transmission process, which in turn informs the risk management measures. At the end of the season, all surveillance results should be analysed, together with other mosquito population parameters, in order to assess the species/population-specific vectorial capacity in the local context.

Active screening of pathogens in larvae, females or males of IMS should be performed during and after MDB outbreaks in order to identify possible trans-ovarial pathogen transmission. A positive result indicates that the

pathogens may be able to overwinter in mosquitoes in temperate regions, which implies that supplementary control measures may be required to prevent/limit further transmission, persistence or spread of the MBD. A negative result, whilst not conclusive, indicates the risk of overwintering is negligible, provided the sampling is sufficiently extensive.

Active screening of pathogens in recently imported or established IMS mosquito females to rule out the possibility of introduction should be carried out even if there is no evidence of MBD pathogen activity. Here, again, negative results do not prove the absence of the pathogen with absolute certainty, but they can be used to reassure the general public and combat media speculation about MBD threats. Also, screening of IMS females contributes to the global screening for a potentially wider circulation of pathogens.

Complementary information provided by screening of pathogens

Pathogen screening in mosquitoes helps to evaluate the virus infection rate, for which the simplest estimate is the minimum infection rate (MIR) for single species or a species group collected in a defined geographical area, and in a defined time period (see Annex 6). Pathogen screening (i) provides a basis for the rapid implementation of risk management measures; (ii) identifies the pathogen species and strain, which may also indicate the pathogen's geographical origin, pathogenicity, and possible genetic mutations/adaptations; and (iii) offers a screening option for pathogens that were not suspected in the area.

It is always an option to implement mosquito/pathogen surveillance, even if there are no reported MBD cases. Surveillance would then serve as a tool for the detection of non-target pathogens; it could also use mosquitoes as 'sentinels', detecting a pathogen before any human cases occur. This approach may, however, be better suited for a bona fide research programme than routine surveillance. Indeed, the economic argument of implementing such methods on a routine basis, particularly if there is no evidence of pathogen circulation, remains questionable and should therefore be evaluated on a case-by-case basis.

Screening for pathogens in mosquitoes is quite expensive, in particular if mosquito sampling is necessary. However, mosquitoes that are collected for surveillance can also be used for the screening of pathogens if trapping and subsequent activities are organised accordingly. In order to optimise detection capacity, mosquitoes need to be collected alive (trap catches collected within 24 hours), transported alive (or stored in dry ice during transport) to the laboratory, rapidly identified using refrigerated plates (chill table), and pooled by species. Pooled samples should be labelled and documented (time, day, and place of collection; see Chapter 2.6). Samples should be stored at −20° C (short term) or at −80° C or below (long term) and should at no stage be thawed and refrozen. If freezing is not possible, samples can be stored in RNA stabilisation buffer (e.g. *RNAlater* by AMBION Inc., USA) at room temperature, but this may hamper successful virus isolation. Pathogen screening should be a two-stage process, first using generic PCR on all samples, followed by specific PCR on samples that tested positive. In all cases, an aliquot of the samples submitted to PCR should be kept for pathogen identification on samples revealed positive by PCR.

Complementary information on methods, analysis and presentation of data from mosquito pathogen screening is provided in Annex 6.

Which pathogen should be checked and how?

Invasive mosquitoes can be screened for arboviruses, or for parasites such as filarial nematode worms, by adapting existing methods. Very few commercial kits are available for analysing samples for arboviruses. However, PCR detection methods are continuously evolving, improving efficiency and reducing costs, and laboratories should be aware of these developments. Optimal handling, preparation and storage of the sampled mosquitoes are prerequisite to obtain good results, and protocols should be defined in collaboration with the laboratory that will perform the analysis. Most European countries have laboratories that are able to provide advice on this topic or provide screening services to determine the infection status of sampled mosquitoes and identify both the species and the strain of pathogens. Some laboratories can also investigate vector susceptibility and competence of mosquito populations. These tests must be performed by laboratories with appropriate biosafety levels.

Once a specific surveillance programme is chosen, it is recommended that networks such as the 'European Network for Diagnostics of "Imported" Viral Diseases' (ENIVD; www.enivd.org) are informed. Surveillance networks can facilitate the search for appropriate laboratories which boast the facilities required for pathogen identification and at the same time meet biosafety requirements. Since there are no diagnostic laboratories focusing only on diagnostics of arboviruses, networks and laboratories in other European countries may be asked for support. In addition, EC-funded projects dealing with vector-borne diseases, e.g. VBORNET (www.vbornet.eu) or EDENext (www.edenext.eu), may be able to assist in identifying the laboratory best able to provide advice and/or support for collaborative studies.

Box 8: Key issues and recommendations for pathogen screening

- Determine surveillance objectives for pathogen screening in IMS according to context.
- Identify a competent and properly equipped laboratory, determine the laboratory analysis method, and adapt the protocol for sample collection and handling accordingly.
- Keep an aliquot of the sample submitted to PCR for further pathogen identification on PCR-positive samples.

2.5 Procedures for the collection of environmental parameters

Figure 12: Surveillance procedures and data collection for environmental parameters and their data sets, according to expected outcomes and Scenarios 1 through 3

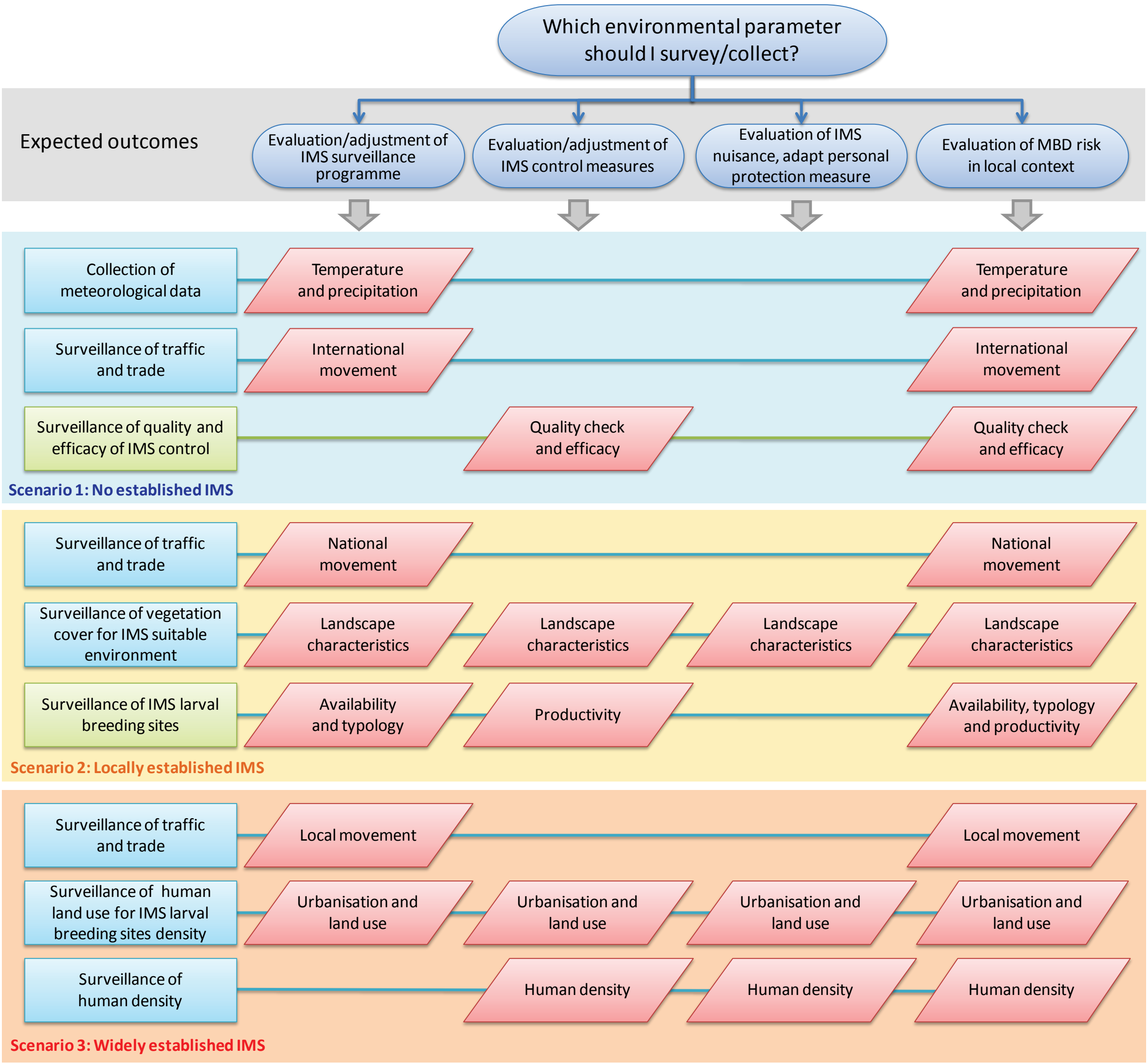

Data sets (red parallelograms), obtained from key procedures (green rectangles) and optional procedures (blue rectangles), may be collected according to the required outputs. Surveillance of traffic and trade is treated in Chapter 2.1 and Annex 3, in conjunction with identification of PoE and spread pathways (not shown here). Procedures listed for one scenario must be maintained in all other scenario(s).

In addition to the biological factors specific to particular mosquito species (see Chapter 2.3 and Annex 5), environmental factors play an important role in determining the IMS' colonisation process, its population size, its vectorial capacity, and consequently the MBD transmission risk. Such factors include (1) availability and type of larval breeding containers, (2) climate change, (3) environmental change, (4) human population density, (5)

increased human travel and goods transport, (6) changes in living and agricultural and veterinary habits (land use), and (7) reduction of resources in the life cycle of mosquitoes by interventions (e.g. source reduction of aquatic habitats).

Human population distributions, urbanisation, and human population movement are the key behavioural factors in most IMS-transmitted diseases because they are related to the global spread of MBD (introduction, reintroduction, circulation) and increase exposure to bites by infected mosquitoes. The world's population is almost equally divided between urban and rural dwellers, and two thirds of Europe's population now live in urban areas, with a similar proportion for the rest of the world projected for 2030. This trend, which is likely to continue for the foreseeable future, may dramatically enhance the reproduction potential of container-breeding IMS by providing more hosts and habitats. The predicted substantial growth of urban and peri-urban agriculture will also create new breeding sites for IMS (see Annex 7) as well as influence the distribution of domestic and wild animals (preferred hosts for *Ae. koreicus*).

In order to obtain spatio-temporal perspectives, environmental data collection and analysis should be carried out when there is a high risk of introduction of IMS to an area. In the case of either scenario 2 or 3 (cf. Chapter 1.3; IMS established over a wide area), the crucial environmental parameters to be considered are the typology, productivity, and distribution of breeding sites. These parameters provide key information needed to calculate population abundance, estimate the spread of invasive mosquitoes, and assess the risk of MBD transmission.

The attractiveness of potential breeding sites to ovipositing mosquito females is affected by many factors, including the types of water containers and their locations. In a recent study in Italy, catch basins in private and public areas were the most productive breeding sites for *Ae. albopictus* among the 10 types checked (catch basins, plant saucers, drums, buckets, tarpaulins, tyres, bathtubs, and assorted containers of three different volumes). The highest number of pupae per premise was found in poorly maintained premises, most often in combination with heavy shade. A thorough knowledge of the most productive breeding sites is needed to choose the most appropriate population index (see also Annex 5, Table A) and establish which site types should be sampled to provide the best indicators of mosquito population abundance. Control programmes may also directly benefit from information on which larval breeding sites are most effective to target. Finally, it would be helpful to obtain information about the quality and efficacy of all conducted IMS control measures (i.e. from Scenario 1; cf. Chapter 1.3), as this will help to later evaluate cost effectiveness and serves to help justify control campaigns.

Table 7 summarises what information needs to be considered for IMS surveillance.

Complementary information on environmental parameters and their acquisition methods can be found in Annex 7.

Box 9: Key issues and recommendations for environmental data

- Key parameters to be collected are (1) quality and efficacy of IMS control measures, and (2) information on larval breeding sites (density, typology and productivity) in order to adapt IMS control.
- Other parameters may be prioritised according to expected outcomes and the scenario.
- The main meteorological factors include (1) temperature, (2) rainfall and humidity, and (3) wind; these data are usually monitored and kept for many locations within each country, but portable meteorological stations can provide data from locations that are not monitored by national meteorological institutions.
- In addition, changes in the monitoring environment (climate, land use) during the colonisation are particularly relevant for the planning of mitigation measures.

Table 7: Main characteristics of environmental parameters to be considered for IMS surveillance

Parameters	Information provided	Strengths	Weaknesses	Data collection methods and equipment
**Breeding sites: typology and productivity**	Information answers the following questions: Where do the mosquitoes breed, what is the relative productivity of the different breeding site types, and what is the geographic distribution throughout the territory?	Good support in the ecological understanding of IMS; identification of targets for IMS control	Requires skilled technicians; high cost	• GIS and field data collection
**Temperature geo-distribution and trend over the year**	Indicates the suitable period for activation of surveillance; feeds the model for IMS risk of establishment and MBD risk assessment; correlates with IMS longevity and vectorial capacity; explains behavioural changes of vector	Data usually available in good detail	Site-specific weather data could not be obtained from local weather stations	• Data from weather stations usually available locally<br>• Field-collected data based on portable weather stations
**Precipitation distribution**	Informs the IMS risk-of-establishment model; correlates with the IMS population density; informs the population estimate models	Data usually available	Large local variability is difficult to define	• Field-collected weather data

Parameters	Information provided	Strengths	Weaknesses	Data collection methods and equipment
**Human population density**	Informs the model for IMS risk of establishment; informs MBD risk assessment	Data usually available in good local detail	Human behaviour can also have an impact on IMS and MBD risks but these data are usually not available	• Socio-statistical data
**Vegetation covering**	Suitability of the area for colonisation and dispersal	Data usually available with good local detail (CORINE data set)	Requires proficient GIS technicians	• Remote sensing data<br>• Satellite imagery
**Human land use in relation to water-keeping habits**	Suitability of the area to be colonised; types of water recipients and land cover to be described in terms of larval breeding sites (potential, availability) and energy resources	Data usually available for public areas, but need to be correlated with specific IMS requirements	Private areas difficult to assess; requires time-consuming research	• Remote sensing data<br>• Satellite imagery<br>• GIS field data collection
**Quality and efficacy of IMS control measures**	Informs the models for cost-effectiveness estimates; evaluation of control methods, including community participation; resistance management	Ensures independent quality control for IMS control programmes	Requires independent, objective and science-based evaluation, as well as skilled technicians	• Internal evaluation<br>• External evaluation

2.6 Methods for data management and analysis

The agencies funding IMS surveillance may consider themselves the sole owners of the collected data and may therefore be reluctant to share them. However, it is important that IMS surveillance data, including pathogen detection data, are made freely available to the public health authorities. Data exchange between competent authorities should be promoted, for example between local and regional/provincial authorities, and between national and international authorities. This is even more crucial if important changes are detected (e.g. introduction of a new IMS, pathogen transmission, or MBD outbreak). The comparison and interpretation of shared data is greatly simplified if data are collected and stored through harmonised procedures.

Basic data management features for storage and analysis

Data collection involves a number of steps including (a) trapping, (b) sample handling, (c) labelling, (d) transportation, (e) diagnostics, (f) populating the database, (g) rearranging and preparing acquired data for analysis. All steps are prone to errors, for example the inaccurate recording of sample locations (can be minimised by using GPS coordinates instead of names, descriptions, or addresses), lost samples, improperly handled/stored samples so further processing and analysis is impossible, labelling errors, unlabelled samples, diagnostic errors, sample mix-ups (‘Did this mosquito come from this vial or that vial?’), data-entry errors, and row/column mix-ups when working on a database spreadsheet. Each of these mistakes will affect the final analysis. Well-thought-through methods and good data management, combined with a heightened awareness of possible mistakes during all steps can help prevent most of these errors.

Box 10: Field sample labelling

Small scale surveillance studies with relatively few samples can probably do with hand-written labels, but if large numbers of samples are expected, labelling needs to be more rigorous. Each label must record a range of information, such as collection date, location (as precise as possible), and type of sampling. One can decide to initially record only the basic sample data on the label and later add the identification results. This approach has the disadvantage that only some information will fit, as space on the label is limited. Consequently, an initial decision has to be made on what to record on the label.

A preferred method is to use unique computer-generated label numbers. When a sample is collected in the field (e.g. a strip with/without mosquito eggs from an oviposition trap), the strip is placed in a sealed bag (only one sample per bag), which is then immediately labelled with a unique number on a sticker. Basic sample data (type of surveillance, date, location) are filled in on either (a) a paper form with a unique number sticker to prevent incorrect numbering, or (b) are entered electronically via a smartphone app connected to a data management system like *Modirisk* (http://www.modirisk.be) or *VecMap* (http://iap.esa.int/vecmap projects). In the laboratory, diagnostics and other useful information derived from the sample should also be entered into the data management system, thus ensuring that each mosquito data set is tied to a unique number and can be traced back.

Data analysis is the end point in a series of steps: (1) defining the type of outputs needed (e.g. presence/absence maps of an IMS in a defined geographical area, and in a defined time period); (2) defining the type of data that is required to produce the desired output (in this example geo-referenced presence/absence data of an IMS in a

defined geographical area, and in a defined time period; in most cases of IMS surveillance, this represents the basic required data); (3) defining the type of surveillance necessary to obtain the required data (see scenarios in Chapter 1.3); and (4) collecting data, which represents the start of data management. Table 8 suggests minimal sets of data for key procedures as well as for some optional procedures. It is suggested that Member States use these standardised data sets to facilitate the collation of data at the EU level.

Table 8: Suggested basic set of variables to be included in databases of surveillance of IMS

8a. Data set for key procedures

Data label	Format
**Level 1 – per sampling**	
**Type of surveillance**	Name
**Type of sampling or trap**	Method or trap model ID
**Date**	DD/MM/YYYY
**Country**	Name
**NUTS**	Code
**Geo-referenced latitude**[1]	DD.NNNNN
**Geo-referenced longitude**[1]	DD.NNNNN
**Altitude**	N
**Data entering**	Name (person)
**Level 2 – per mosquito species**	
**Mosquito species**	Species name or ID
**Presence/absence**	1/0
**Female**	N
**Male**	N
**Pupa**	N
**Larva**	N
**Egg**	N
**Identifying person**	Name (person)
**Method of identification**[2]	Name (from a list)
**Validator**	Name (person)

[1] Latitude and longitude of the sampling point, UTM WGS 84 system, decimal degrees units
[2] Morphology/molecular (gene)/MALDI-TOF

8b. Data set for optional procedures (additional to previous data)

Data label	Format
**Level 1 – per sampling**	
**Temperature**	N
**Relative humidity**	N
**No. of mosquitoes tested**	N
**Data entering**	Name (person)
**Level 2 – per mosquito species**	
**Physiological status**	Blood fed/unfed/gravid/nulliparous
**No. of mosquitoes tested**	N
**No. of pools tested**	N
**Pathogen name, positive pools**	N

N = numeric field

Database

Most researchers are comfortable with spreadsheet software such as Microsoft Excel. Such software is useful for the extraction of data from a large database, but for more complex analysis and mapping, it is recommended that data are stored in a more specific database management system that allows networking. Examples of frequently used relational database management systems (RDBMS) are Microsoft Access, SQL Server, DB2, and Oracle Database. By using an RDBMS, data integrity can be ensured, e.g. a properly defined rule can ensure that data are entered in a valid format. Additionally, these systems can handle an almost unlimited amount of data. These

programmes also allow users to be allocated various levels of access rights to use/import/see the data, by assigning them login and passwords (e.g. the analyst may be allowed/not allowed to modify surveillance data).

An example of a database application is given in Annex 8, Figure A.

For GIS analysis or modelling, data can be imported into statistical software packages or dedicated geographic information systems software such as ArcGIS, GRASS, or QGIS. Currently, a new system named VecMap is under development, integrating the entire process of producing vector risk maps into a single package by combining various functions such as data collection, risk map production, data storage, data analysis, and statistical distribution modelling based on weather data and satellite imagery. The system is funded by the European Space Agency and currently in its demonstration phase. It is expected to be available in 2013 (http://www.esa.int/esaCP/SEMIZ5MSNNG_index_0.html).

In addition, most groups that carry out IMS surveillance simultaneously develop more than one type of surveillance programme. It is advisable for each group to standardise data management and processing as much as possible, entering and storing all surveillance data into one well-designed customised and centralised database with online access, managed by a group of experts with database administration rights. The advantages of a centralised database are that (1) there is no need to extract data from different databases to be able to compare data from separate studies; (2) it is less likely that data get lost; (3) there is a consistent user interface for database access; and, perhaps most importantly, (4) data integrity is ensured (e.g. avoiding 'wrong doubles').

Complementary information on data management and analysis is given in Annex 8.

Box 11: Key issues and recommendations for data management and analysis

- Harmonise data management through standardisation.
- Ensure at each step that samples are corrrectly labelled.
- Ensure long-term storage and continuity of data sets on surveillance activities.

2.7 Strategies for data dissemination and mapping

Figure 13: Dissemination/communication of IMS surveillance outcomes and effects

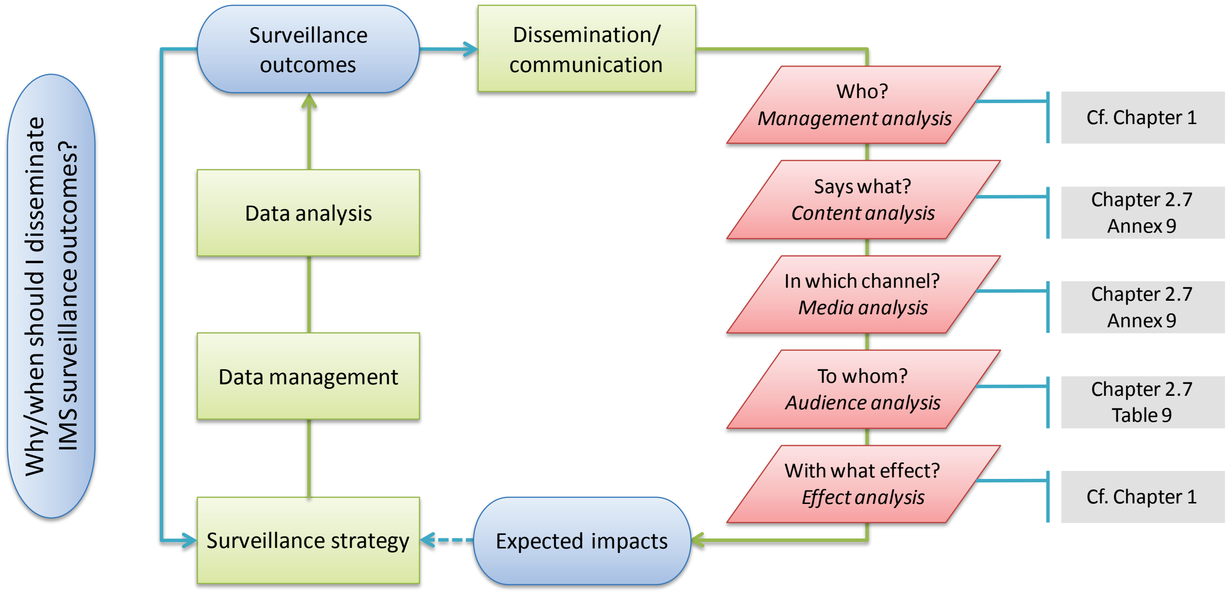

Red parallelogram chain corresponds to Lasswell's communication model

Surveillance of IMS produces a wide range of data that need to be disseminated to different stakeholders. The method of dissemination depends on (a) the communicator, (b) the message and thus the type of data and level of detail, (c) the selected presentation medium (charts, maps, plain text), (d) the recipients (both end-users and non-technical audiences), and (e) the desired social, political, or scientific impact and any feedback envisaged (cf. Figure 13, Lasswell's model). The timing (e.g. during an MBD outbreak) and the cost of the dissemination method also have to be considered.

Dissemination pathways for stakeholders, end-users and other target audiences (general public, non-governmental organisations, etc.) should be carefully developed, taking into account the sensitivity of the information. The choice

of data type, level of detail and presentation style (charts, maps, plain text, etc.) should be tailored to the target group and designed to deliver the desired impact. The language used may depend on the scale of the dissemination campaign. The timing of a campaign should be set according to the desired response from the target group: launching a campaign before the mosquito breeding season differs substantially from a launch during the season when the general public is already acutely aware of the nuisance caused by mosquitoes.

Scope and audience

Table 9 sets out suggestions on target audience in relation to the prioritisation of data dissemination for Scenarios 1 through 3. It is important that the local context is carefully assessed when defining the communication strategy, in particular the mechanisms of collaboration between agencies and the required level of community participation.

Table 9: Main audiences and prioritisation of data dissemination for Scenarios 1 through 3 and related issues

Main surveillance outcomes	National authorities	Regional/pro-vincial authorities	Local authorities	Mosquito control units	Health units	Environment agencies	Scientific community	Media	General public
Scenario 1: No established IMS									
Existing surveillance programme and absence of IMS	1	1	1	2				2	2
Observations of introduction of IMS and implementation of control programmes and their effectiveness	1	1	1	1	2	2	2	2	2
Scenario 2: Locally established IMS									
Presence of IMS and existing surveillance programme	1	1	1	1	2	2	1	1	1
Observed spread of IMS and/or new introductions	1	1	1	1	2	1	1	1	1
Identification of introduction pathways	1	1	2	2	2	2	1		
Implementation of control programmes and their effectiveness	1	1	1	1	2	2	1	1	1
Scenario 3: Widely established IMS									
Presence of IMS and existing surveillance programme	1	1	1	1	2	2	1	2	2
Observed spread of IMS and/or new introductions	1	1	1	1	2	1	1	2	2
Identification of pathogens in mosquitoes	1	1	1	1	1	2	1	2	2
Implementation of control programmes and their effectiveness	1	1	1	1	1	2	1	2	2

1= priority; 2= secondary. Audiences listed as '1' should be sent the surveillance information as soon as possible, whereas audiences listed as '2' may be informed later. However, these priorities must be reassessed/adapted according to the local situation.

The scope and methodologies of IMS surveillance differ greatly between areas, local situations, and scenarios. Consequently, data dissemination and communication approaches will also differ. In general, precautions should be taken to avoid misinterpretations of both surveillance results and established risk levels.

The discovery of IMS (introduction, establishment, spread, infection by pathogens, etc.) should be reported as soon as possible to all national authorities to allow rapid customisation of risk plans and implementation of customised control measures. Data exchange, however, in particular for data on detection and establishment of IMS as well as results of investigations on IMS introduction and spread pathways, should also include neighbouring countries and regions. Informing neighbouring countries promptly will allow them to rapidly implement measures which will be beneficial for the whole region. If a new invasive species is found, it is also advisable to inform the scientific community and in particular the EMCA, preferably through EMCA national directors, as scientists often perform mosquito monitoring even if there are no formally validated surveillance plans. Also, the medical profession in affected cities and regions should be quickly informed about IMS and MBD risk (and MBD symptoms), in particular when there are changes in the IMS and MBD situation (evidence of circulation of pathogens, adult mosquito activity period, etc.).

Since IMS and urban areas are strongly connected, larval control is most efficient if the community participates. In some countries, gaining entrance to private properties to carry out mosquito surveillance and/or control is difficult, which may hinder larval habitat treatment and hampers the elimination of breeding containers (see Annex 9, Box A). It has been shown that community participation can be improved through education and diffusion of information. This can be achieved at the level of municipalities, provinces, and (local or national) public health institutes, but also by providing lesson plans for primary and/or secondary/high schools, public awareness

messages on TV and other media, or door-to-door leaflets, etc. This topic is covered in more detail in the upcoming EMCA/WHO strategic document.

Dissemination methods

Depending on the type of audience, results can be disseminated using a variety of methods which should be tailored to the particular target audiences, designed to achieve the desired impact, and customised to the type of information to be disseminated. A dedicated website should state the rationale behind the campaign, provide programme descriptions, and track progress and results. Online maps are an ideal data presentation format, presenting information in an easy-to-understand way, while also explaining IMS distribution changes in a defined area (see next chapter).

Complementary information the dissemination of surveillance results is provided in Annex 9: Table A lists the most suitable dissemination methods based on the type of surveillance data, while Table B offers an overview of various dissemination approaches and their strengths and weaknesses in terms of data formats, evaluation of efficiency, and intended audiences.

Mapping strategy

The bulk of surveillance data will be geo-referenced information on the presence/absence of IMS over a defined time period (day, week, month, year). It should be noted that when no IMS are reported at a given location, this does not prove that IMS are not present at or near the sample location. Mosquitoes may not be detected because the weather conditions were unfavourable, traps were not effective for that particular species (bait is often specific to a more or less limited range of species), or the mosquito density was below the detection threshold. However, sampling throughout the season will increase data accuracy, making it possible to interpret negative results as true absences. Figure 14 shows different mapping outcomes for surveillance data of *Ae. albopictus* gathered in the Netherlands, 2011, using different scales and based on different levels of data accuracy.

Surveillance data can be disseminated via maps which can depict (1) the presence /absence of IMS for all scenarios, (2) abundance levels of an IMS in scenarios 2 and 3, and even (3) show the presence of MBD pathogens in field-collected mosquitoes in scenario 3. Such data can be extrapolated by modelling for predicting possible IMS distributions or using known IMS vectorial capacity within an R_0 model to mapping MBD transmission risk for a defined period.

Figure 14: Examples of maps on IMS presence/absence, showing surveillance results for *Ae. albopictus* in the Netherlands, 2011

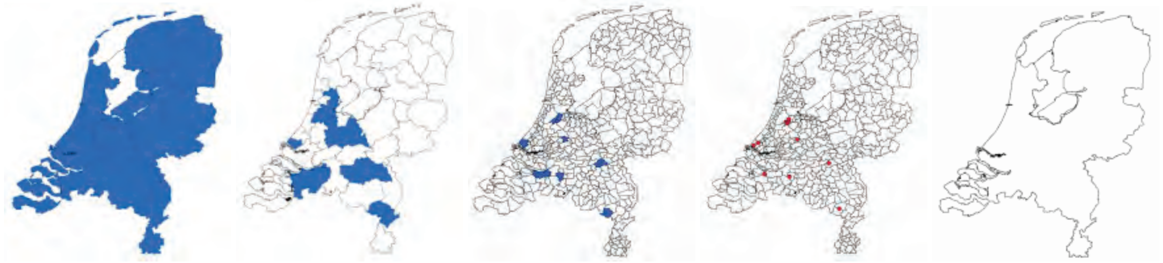

A. Country level (positive/negative for IMS)

B. NUTS 3 level (positive/negative for IMS)

C. Municipality level (positive/negative for IMS)

D. Presence points (positive/negative for IMS)

E. Areas with established population

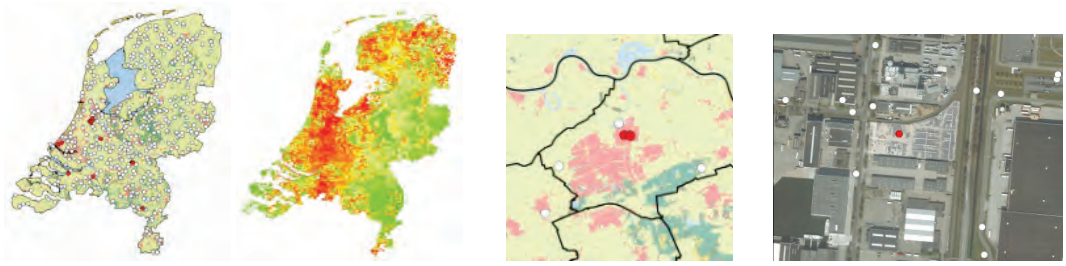

F. Presence/absence points (x, y coordinates) (red = positive, white = negative)

G. Predictive modelling based on presence/absence points

H. Presence points (x, y) at municipality level

I. Presence/absence points outdoors per trap (red = positive, white + negative)

Blue areas and red dots indicate true presences; white areas and white dots can be considered as true absences as they are based on several sampling dates throughout the season. Map G predicts suitability for the indigenous mosquito Culiseta annulata.

Source: Dutch National Centre for Monitoring of Vectors (CMV), NVWA

3 Cost and evaluation of the defined surveillance programme

3.1 Estimating cost of surveillance activities

Surveillance activities associated with the three scenarios (see Chapter 1.3) are supposed to achieve specific goals and deliver risk estimates in the most cost-effective way. The following section provides cost estimates of the various activities associated with all key and some optional procedures. Tables 10a, 10b, and 10c list procedures and tasks and their cost equivalent expressed in working days (upper values). Also listed are additional costs for 12 months of surveillance (lower values; i.e. travel costs, consumables, but excluding investments for equipment, e.g. traps, computers, software).

Table 10a: Cost estimate for key and optional procedures, Scenario 1

Procedures and tasks	No. of sites	Field investigations[1]	Laboratory investigations	Data processing[2]	Communication/ dissemination	Total key procedures	Total optional procedures
**IMS surveillance of introduction at points of entry**						**301 wd<br>27 200 EUR**	**30 wd<br>1 170 EUR**
Storage sites for used tyres	10	80 wd<br>12 000 EUR	20 wd<br>200 EUR	5 wd<br>5 EUR	5 wd<br>10 EUR	110 wd<br>12 215 EUR	-
Shelters/greenhouses for imported cutting plants like *Dracaena* spp. (Lucky Bamboo)	5	40 wd<br>3 600 EUR	10 wd<br>100 EUR	3 wd<br>5 EUR	3 wd<br>10 EUR	56 wd<br>3 715 EUR	-
Main parking lots at country borders, highways and road axes that originate in colonised areas	20	80 wd<br>9 600 EUR	20 wd<br>200 EUR	5 wd<br>5 EUR	5 wd<br>10 EUR	110 wd<br>9 815 EUR	-
Ports	1	16 wd<br>1 440 EUR	5 wd<br>5 EUR	2 wd<br>5 EUR	2 wd<br>5 EUR	25 wd<br>1 455 EUR	-
Airports	1	16 wd<br>960 EUR	5 wd<br>5 EUR	2 wd<br>5 EUR	2 wd<br>5 EUR	-	25 wd<br>975 EUR
Quality and efficacy of control measures	1	2 wd<br>180 EUR	1 wd<br>5 EUR	1 wd<br>5 EUR	1 wd<br>5 EUR	-	5 wd<br>195 EUR

[1] *Includes field work, training, travel*
[2] *Includes management, analysis, dissemination, and mapping*
wd = working day

Table 10a is based on Scenario 1 and lists costs for activities at several sites. In Table 10b, costs are estimated for the additional activities that are required under Scenario 2 (small colonised area). Table 10c is based on Scenario 3 (large colonised area). In Tables 10b and 10c, community participation is assumed. Costs given are based on EU mean prices. The number of sites to be surveyed and the size of the chosen areas are set arbitrarily and should be adjusted to meet local conditions when calculating working days and other costs. Detailed cost estimates are shown in Table 11.

Table 10b: Cost estimate for key and complementary procedures, Scenario 2

Procedures and tasks	Size of area	Field investigations[1]	Laboratory investigations	Data processing[2]	Communication/ dissemination	Total key procedures	Total optional procedures
**IMS surveillance of introduction at points of entry***						**301 wd<br>27 200 EUR**	**30 wd<br>1 170 EUR**
**IMS surveillance of persistence on colonised area**						**44 wd<br>6 725 EUR**	**50 wd<br>1 735 EUR**
Inspection of colonised areas	2 km²	16 wd<br>1 200 EUR	5 wd<br>25 EUR	3 wd<br>5 EUR	5 wd<br>5 000 EUR[1]	29 wd<br>6 230 EUR	-
Quality and efficacy of control measures	2 km²	8 wd<br>480 EUR	3 wd<br>5 EUR	2 wd<br>5 EUR	2 wd<br>5 EUR	15 wd<br>495 EUR	-
Abundance and seasonal dynamics	2 km²	24 wd<br>1 440 EUR	10 wd<br>30 EUR	5 wd<br>5 EUR	2 wd<br>5 EUR	-	41 wd<br>1 480 EUR
Biting behaviour	2 km²	4 wd<br>240 EUR	2 wd<br>5 EUR	1 wd<br>5 EUR	2 wd<br>5 EUR	-	9 wd<br>255 EUR
**IMS surveillance of spread around colonised areas**						**90 wd<br>6 500 EUR**	**-**
Inspections around colonised areas	20 km²	48 wd<br>1 440 EUR	15 wd<br>40 EUR	7 wd<br>5 EUR	5 wd<br>5 000 EUR[1]	75 wd<br>6 485 EUR	-
Quality and efficacy of control measures	2 km²	8 wd<br>480 EUR	3 wd<br>5 EUR	2 wd<br>5 EUR	2 wd<br>5 EUR	15 wd<br>495 EUR	-

[1] Includes field work, training, travel
[2] Includes management, analysis, dissemination, and mapping
** See details in Table 10a*

Table 10c: Cost estimate for key and complementary procedures, Scenario 3

Procedures and tasks	Size of area	Field investigations[1]	Laboratory investigations	Data processing[2]	Communication/ dissemination	Total key procedures	Total optional procedures
**IMS surveillance of introduction on points of entry***						**301 wd<br>27 200 EUR**	**108 wd<br>2 947 EUR**
**IMS surveillance of persistence on colonised area**						**169 wd<br>11 380 EUR**	**24 wd<br>1 850 EUR**
Inspection of colonised areas	50 km²	80 wd<br>2 400 EUR	15 wd<br>150 EUR	7 wd<br>5 EUR	10 wd<br>5 000 EUR[1]	112 wd<br>7 555 EUR	-
Quality and efficacy of control measures	50 km²	8 wd<br>720 EUR	3 wd<br>50 EUR	2 wd<br>5 EUR	2 wd<br>5 EUR	15 wd<br>780 EUR	-
Infection of IMS by pathogens	50 km²	24 wd<br>2 160 EUR	5 wd<br>500 EUR	2 wd<br>5 EUR	1 wd<br>5 EUR	32 wd<br>2 670 EUR	-
Abundance and seasonal dynamics	50 km²	4 wd<br>360 EUR	3 wd<br>5 EUR	2 wd<br>5 EUR	1 wd<br>5 EUR	10 wd<br>375 EUR	-
Biting behaviour	50 km²	16 wd<br>1 440 EUR	4 wd<br>400 EUR	3 wd<br>5 EUR	1 wd<br>5 EUR	-	24 wd<br>1 850 EUR
**IMS surveillance of spread around colonised areas**						**142 wd<br>7 250 EUR**	**-**
Inspections around colonised areas	250 km²	80 wd<br>2 160 EUR	25 wd<br>70 EUR	12 wd<br>5 EUR	10 wd<br>5 000 EUR[3]	127 wd<br>7 235 EUR	-
Quality and efficacy of control measures	50 km²	8 wd<br>720 EUR	3 wd<br>50 EUR	2 wd<br>5 EUR	2 wd<br>5 EUR	15 wd<br>780 EUR	-

[1] Includes field work, training, travel
[2] Includes management, analysis, dissemination, and mapping
[3] Editing and printing costs for a flyer
** See details in Table 10a*

Table 11: Detailed cost estimate by procedure

Table 10a	No. of sites	Distance[1]	Rate (EUR)	No. of visits	EUR	No. of sites/day	wd
Storage sites for (imported) used tyres	10	250	0.3	16	**12000**	2	**80**
Greenhouses	5	100	0.3	24	**3600**	3	**40**
Parking lots	20	100	0.3	16	**9600**	4	**80**
Port	1	300	0.3	16	**1440**	1	**16**
Airport	1	200	0.3	16	**960**	1	**16**
Efficacy of control	1	300	0.3	2	**180**	1	**2**
**Table 10b**	**km²**	**Total distance[2]**	**Rate**	**No. of visits**	**EUR**	**No. of days/visit**	**wd**
Inspection colonised area	2	250	0.3	16	**1200**	1	**16**
Efficacy of control	2	200	0.3	8	**480**	1	**8**
Abundance	2	200	0.3	24	**1440**	1	**24**
Biting behaviour	2	200	0.3	4	**240**	1	**4**
Inspection around	20	300	0.3	16	**1440**	3	**48**
Efficacy of control	2	200	0.3	8	**480**	1	**8**
**Table 10c**	**km²**	**Total distance[2]**	**Rate**	**No. of visits**	**EUR**	**No. of days/visit**	**wd**
Inspection colonised area	50	500	0.3	16	**2400**	5	**80**
Efficacy of control	50	300	0.3	8	**720**	1	**8**
Pathogens	50	300	0.3	24	**2160**	1	**24**
Abundance	50	300	0.3	4	**360**	1	**4**
Biting behaviour	50	300	0.3	16	**1440**	1	**16**
Inspection around	250	900	0.3	8	**2160**	10	**80**
Efficacy of control	50	300	0.3	8	**720**	1	**8**

[1] Mean distance between sites
[2] Total distance to be covered

No. of traps and visits are in accordance with recommendations given in Table 4, for a whole year. For part 10a, formulae are: cost = no. site x distance x km rate x no. visit; working days = no. site/no. sites per day x no. visits.
For parts 10b and 10c, formulae are: cost = total distance x km rate x no. visit; working days = no. visits x no. days per visit.
Visits for 'efficacy of control' are calculated for one control campaign (10c), and for four treatments (10b and 10c).

The time necessary to identify the sites to be surveyed and to make arrangements to obtain the necessary agreements is not included. Investment costs should be added (e.g. for traps: around EUR 150 for a CO_2-baited trap, a light trap, or a BG-Sentinel trap; EUR 600 for an MM trap; EUR 180 for a gravid trap; EUR 3 for a sticky trap; and EUR 1 for an ovitrap). All these costs should be customised to the local context (i.e. number of sites and size of areas, local rates, and specific constraints).

3.2 Evaluation of the operational surveillance process

It is suggested that (1) documentation of the surveillance programme is planned from the beginning in order to facilitate future evaluation, (2) internal evaluations of the implemented surveillance programme are conducted annually, and (3) an external evaluation is carried out every five or ten years.

The purpose of an evaluation is to assess how the surveillance is organised and managed, to critically examine achievements and constraints, to analyse cost effectiveness, and to provide recommendations for possible improvements. The evaluation of the programme should focus on the overall management, the internal coherence and clarity of the objectives, the sharing of responsibility, the internal and external communication procedures, and the quality of the results. Attention should be paid to the sustainability of support and commitment displayed by national/regional/local governments and stakeholders. Networking and partnership activities with other relevant entities should not be neglected.

Annex 1: Basic information on the biology of mosquitoes and the transmission of vector-borne diseases

Box A: Invasive mosquito species (IMS) names

'Major generic changes in the tribe Aedini were recently published (Reinert 2000; Reinert et al. 2004, 2006, 2008), leading to a scientific debate and two or more names being simultaneously used for a single taxon.' (Medlock et al., 2012).
Editors of several scientific journals suggest that the traditional names (JME Editors 2005) should be kept until there is a consensus on this major nomenclature change. In this guideline document we use the traditional names, with alternate names shown here:

Aedes aegypti, also known as *Stegomyia aegypti* sensu Reinert et al. 2004.

Aedes albopictus, also known as *Stegomyia albopicta* sensu Reinert et al. 2004.

Aedes atropalpus, also known as *Ochlerotatus atropalpus* sensu Reinert et al. 2004 and *Georgecraigius atropalpus* sensu Reinert et al. 2006.

Aedes japonicus, also known as *Ochlerotatus japonicus* sensu Reinert et al. 2004 and *Hulecoeteomyia japonica* sensu Reinert et al. 2006.

Aedes koreicus, also known as *Ochlerotatus koreicus* sensu Reinert et al. 2004 and *Hulecoeteomyia koreica* sensu Reinert et al. 2006.

Aedes triseriatus, also known as *Ochlerotatus triseriatus* sensu Reinert et al. 2004.

The biology of mosquitoes

Mosquitoes belong to the family Culicidae (order Diptera, suborder Nematocera), subfamily Culicinae. A total of 3,523 species of Culicidae are currently recognised worldwide (Harbach 2011, Mosquito Taxonomic Inventory, http://mosquito-taxonomic-inventory.info, accessed 24 January 2012). Mosquitoes are ecologically beneficial for their contribution to biodiversity, food chains, pollination, and only some species are sources of threat to human and animal health because of their role as vectors of disease pathogen. About 20 species are known to have significantly extended their distribution range in recent years.

The mosquito biological cycle comprises larva (four instars), pupa, adult, and egg (Figure A).

Eggs are laid by the female, either individually (e.g. *Aedes*) or grouped in egg rafts (e.g. *Culex*), on the water surface (e.g. *Anopheles*, *Culex*), on the edge of water bodies, or on wet ground (i.e. *Aedes*). Eggs laid on the water surface generally hatch soon after deposition, whilst those not laid directly in water bodies hatch after flooding. *Aedes* eggs laid on the ground will remain quiescent for up to a year (univoltine species) or even several years if not flooded. Some species overwinter as mated females (e.g. *Anopheles*, *Culex*) or as eggs which are highly resistant to desiccation and low temperature (e.g. *Aedes*).

From the egg a young larva hatches directly into the water, where it eats by filtering particles suspended in the water or laying on the bottom (organic matter, algae, bacteria, fungi). It grows in stages of four instars and moults, during which its size increases from about 2 mm (L_1) to about 10 mm length (L_4). The final larval moult produces the pupa, which does not feed. During the pupal phase a complete transformation known as metamorphosis takes place, which creates the final adult stage. The four larval instars and the pupa are aquatic, but need air to breathe, and so need to come to the water surface, which means they can survive in polluted water with low oxygen content. However a few species are specialised to exploit oxygen from aquatic plant tissues (i.e. *Coquillettidia*). In the most favourable conditions of food availability and water temperature (20−25 °C), the aquatic phase is completed in less than a week.

Figure A: Biological cycle of a mosquito (subfamily Culicinae)

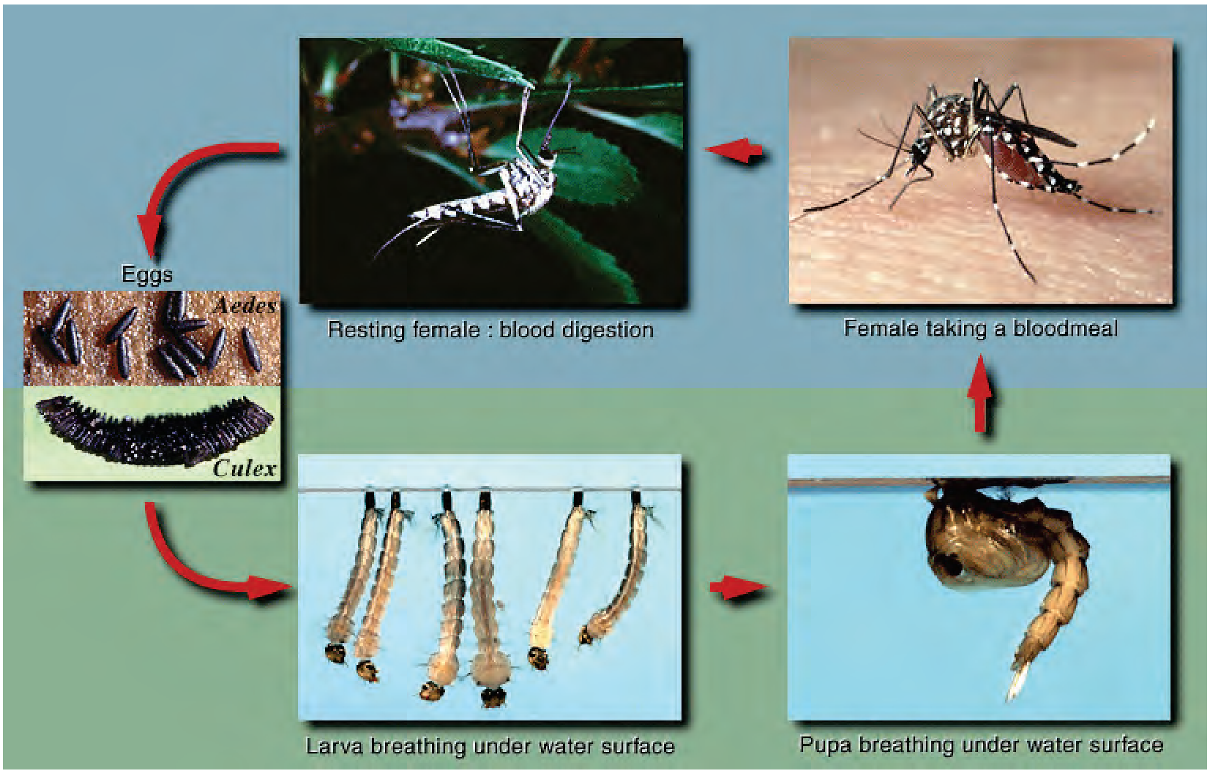

Source: Schaffner F, Angel G, Geoffroy B, Hervy JP, Rhaiem A, Brunhes J. The mosquitoes of Europe. An identification and training programme [CD-ROM]. Montpellier: IRD Éditions & EID Méditerranée; 2001.

The adult, male or female, emerges from the pupa by shedding the pupal skin in water, then rests a short time on the surface and eventually flies away. Mating occurs during flight, with males swarming to attract conspecific females. Monogamy is considered the rule for females, while a male remains sexually active after mating. Adults take sugar meals to obtain energy from plant fluids (i.e. nectars, fluids, decaying fruits). Females also need to take additional blood meals from animals (mammals, birds, amphibians, and reptiles) to provide the proteins needed to mature their eggs. It is this behaviour which makes mosquitoes so important to public health because during blood meals the female may ingest pathogens from an infected host. The female injects saliva into the host when feeding to stop blood coagulation, and a number of pathogens have evolved to accumulate in the salivary glands to exploit this route to parasitise their host. The female may therefore be able to infect a new host during a subsequent blood meal. We therefore call the mosquito a 'vector' (transmitter) due to their capacity to spread viruses, protozoa, filarial worms, or bacteria, causing human and animal diseases which may seriously affect the lives of millions of people. *Aedes* females bite and rest mostly outdoors, whereas *Culex pipiens* (the house mosquito) bites and rests mostly indoors. Females may take several blood meals during their lifespan, which is usually three to four weeks, depending on weather conditions and predation.

Transmission of vector-borne diseases (VBD)

Table A: Important mosquito-borne pathogens that cause disease in humans

Pathogen	Disease	Case fatality rate (%)	Important vectors to human
***Togaviridae* arboviruses**			
**Chikungunya**	Febrile to severe illness	Very low	*Ae. aegypti, Ae. albopictus*
**Eastern equine encephalitis**	Encephalitis	50–75	*Coquillettidia perturbans, Ae. vexans*
**Ross River**	Febrile	0	*Culex annulirostris*
**Sindbis**	Febrile	0	*Ae. cinereus, Cx. pipiens*
**Venezuelan equine encephalitis**	Encephalitis	0.1–20	*Cx. pipiens*
**Western equine encephalitis**	Encephalitis	5–10	*Cx. tarsalis*
***Flaviviridae* arboviruses**			
**Dengue 1-4**	Febrile to haemorrhagic	3–12	*Ae. aegypti, Ae. albopictus*
**West Nile**	Febrile to encephalitis	3–15	*Culex spp.* (*Cx. pipiens, Cx. modestus*)
**Japanese encephalitis**	Encephalitis	30–40	*Cx. tritaeniorhynchus*
**Murrey Valley encephalitis**	Encephalitis	20–70	*Cx. annulirostris*
**St. Louis encephalitis**	Encephalitis	4–20	*Cx. pipiens, Cx. nigripalpus*
**Yellow fever**	Haemorrhagic	5–20	*Ae. aegypti, Ae. africanus, Haemagogus* spp.
***Bunyaviridae* arboviruses**			
**La Crosse encephalitis**	Encephalitis	<1	*Ae. triseriatus*
**Rift Valley fever**	Febrile	<1	*Aedes spp., Cx. pipiens*
***Plasmodium* protozoa**			
**Malaria**	Febrile to renal failure	1–7 (< 5 years)	*Anopheles* spp.

Source: Beaty & Marquardt 1996; Schaffner 2003

Given suitable climate and availability of infection sources, the likelihood of vector-borne infections increases with vector abundance. Transmission may be also possible with low or moderate vector densities if the vector population has a high competence (% of infectious females).

Changes in climate and environment mean that potential vectors and, with them, diseases that they can transmit can spread unnoticed to 'emerge' in new areas. Such spreading may not only be caused by active vector migration but can be due to their passive transport, for example by vehicles, trains and aircraft. Perhaps more importantly, the explosive increase and globalisation of international and intercontinental travel and trade increases the risk of accidental import of vectors and vertebrate reservoir hosts (i.e. vertebrates that carry a pathogen). Control programmes may therefore need to shift from a pest control strategy to a vector control strategy in order to make it possible to customise and improve the efficiency of control methods (e.g. by supplementing or replacing larviciding with adulticiding for better and quicker control).

General bibliography on Culicidae

Beaty BJ, Marquardt WC, editors. 1996. The biology of disease vectors. Niwot, Colorado: University Press of Colorado; 1996.

Becker N, Petrić D, Zgomba M, Boase C, Madon M, Dahl C, et al. Mosquitoes and their control. Heidelberg, Dordrecht, New York: Springer; 2010.

Clements AN. The biology of mosquitoes. Vol. 1 Development, nutrition, and reproduction. London: Chapman & Hall; 1992.

Clements AN. The biology of mosquitoes. Vol. 2 Sensory reception and behaviour. Oxon & New York: CABI Publishing; 1992.

Focks DA. A review of entomological sampling methods and indicators for dengue vectors. Geneva: WHO; 2003.

Gratz NG. Vector- and rodent-borne diseases in Europe and North America. Disctibution, public health burden and control. Cambridge: Cambridge Univ. Press; 2006.

Gratz NG. Emerging and resurging vector-borne diseases. Annu Rev Entomol. 1999;44:51-75.

JME Editors. Journal policy on names of Aedine mosquito genera and subgenera. J Med Entomol. 2005;42(5):511.

Kampen H, Schaffner F. Chapter 13. Mosquitoes. In: Bonnefoy X, Kampen H, Sweeney K, editors. Public Health Significance of Urban Pests. Geneva: WHO Regional Office for Europe; 2008. p.347-86.

Medlock JM, Hansford KM, Schaffner F, Versteirt V, Hendrickx G, Zeller H, et al. A review of the invasive mosquitoes in Europe: ecology, public health risks, and control options. Vector Borne Zoonotic Dis. 2012 Jun;12(6):435-47.

Reinert JF. New classification for the composite genus *Aedes* (Diptera: Culicidae: Aedini), elevation of subgenus *Ochlerotatus* to generic rank, reclassification of the other subgenera, and notes on certain subgenera and species. J Am Mosq Control Assoc. 2000;16:175-88.

Reinert JF, Harbach RE, Kitching IJ. Phylogeny and classification of Aedini (Diptera: Culicidae), based on morphological characters of all life stages. Zool J Linn Soc. 2004;142:289-368.

Reinert JF, Harbach RE, Kitching IJ. Phylogeny and classification of *Finlaya* and allied taxa (Diptera: Culicidae: Aedini) based on morphological data from all life stages. Zool J Linn Soc. 2006;148(1):1–101.

Reinert JF, Harbach RE, Kitching IJ. Phylogeny and classification of *Ochlerotatus* and allied taxa (Diptera: Culicidae: Aedini) based on morphological data from all life stages. Zool J Linn Soc 2008;153:29-114.

Schaffner F, Angel G, Geoffrey B, Hervy J-P, Rhaiem A, Brunhes J. The mosquitoes of Europe. Montpellier: IRD & EID Méditerranée; 2001. CD-ROM.

Schaffner F, Delécolle J-C. Chapter 12: Vectors of arboviruses. In: Lefèvre P-C, Blancou J, Chermette R, Uilenberg G., Editors. Infectious and Parasitic Diseases of Livestock. Paris: Ed. Tec & Doc, Ed. Med. int.; 2010. p137-53.

Schaffner F. The mosquitoes and the community. Local authorities, health and environment briefing pamphlets series, 39. Copenhagen: WHO Regional Office for Europe; 2003.

Scholte E-J, Schaffner F. Chapter 14. Waiting for the tiger: establishment and spread of the *Aedes albopictus* mosquito in Europe. In: Takken W, Knols BGJ, editors. Emerging Pests and Vector-Borne Diseases in Europe. Book series: Ecology and control of vector borne diseases. Wageningen: Wageningen Academic Publishers; 2007. P.241-60.

Service M. Mosquitoes (Culicidae). In: Lane RP, Crosskey RW, editors. Medical insects and arachnids. London: Chapman & Hall; 1993. p120-240.

Service MW. Mosquito ecology: field sampling methods. 2nd ed. London: Elsevier. 1993.

Soumahoro MK, Boelle PY, Gaüzere BA, Atsou K, Pelat C, Lambert B, et al. The Chikungunya epidemic on La Réunion Island in 2005–2006: A cost-of-illness study. PLoS Negl Trop Dis. 2011 Jun;5(6):e1197.

Annex 2: Organisation and management of IMS surveillance in the European context

Distribution of actions and tasks between the possible management levels

International level

No international regulations apply specifically to IMS, except when they are vectors: the International Health Regulations (IHR (2005) and in particular Annex 5 (http://www.who.int/ihr/IHR_2005_en.pdf)) recommend that (i) aircraft and ships should be kept free of vectors by applying insecticides at the beginning of journeys originating in countries where vectors can carry an infectious agent (mainly endemic and epidemic areas of malaria, dengue or chikungunya), and (ii) ports and airports should be kept free of possibly infected vectors (up to 400 metres away).

An important improvement to the existing IHR (2005) would be the introduction of more specific regulations or recommendations about the movements of goods and people which may become involuntary carrier of vectors, together with the identification of national responsibilities for the related implementation and control activities. Moreover, measures to regulate the trade of goods that have been proven to provide passive transportation of IMS should be promoted as EU recommendation.

In addition, information on traffic of goods that represent a risk for the spread of IMS and on movements of people provide valuable data for identifying sites at risk for introduction of IMS and possibly related MBD. This information is often not available in the countries. Centralisation at the European level would make this helpful information available to all stakeholders.

National level

Risk assessment and management plans for IMS and possibly related MBD are best developed at the national level, in collaboration with the regional/provincial level. Relevant issues include the following:

- Analysis of the possible pathways of introduction and PoE for IMS. Factors to be considered are: (i) the trade of goods that have been proven to provide passive transportation of IMS (i.e. used tyres, cuttings of some ornamental plants in containers), (ii) the parking lots and other frequent stops on highways and main roads connected to colonised areas within 600 km, (iii) the ports and airports that have connections with countries where invasive species are widespread, (iv) the main railway stations and truck stops/parking/transiting sites linked to countries where invasive species are widespread.
- Determining the country of origin of goods: some goods that originate from outside the EU are not easily recognised as such; it is common in the used tyre trade to finalise customs and import procedures in one EU country and then move the goods to another EU country, thus effectively concealing the true origin of the tyres).
- Identification, listing and mapping of active surveillance sites identified to be at high risk of introduction of IMS due to the factors described above (bullet point 1).
- Defining which mosquito species may be introduced and specifying the seasonal period to be covered by the surveillance activity, while considering the effect of local climate parameters on the risk of establishment following introduction.
- Defining mosquito collection strategies and surveillance methods (e.g. early detection, spread, abundance, infection by pathogen, etc.), as dictated by the targeted mosquito species or group of species.
- Defining mosquito species identification (both morphology and molecular based) and pathogen identification capacities in the country, including reference centres.

Enlisting the help of the regional/provincial stakeholders while setting up a surveillance programme will make better use of in-country competence and help to design a programme that the regional/provincial resources have the capacity to implement.

Regional/provincial level

Plans for IMS and MBD prevention/control are best developed at the regional/provincial level, in the context of a national strategy and in cooperation with local authorities (local public health units, municipalities) and technical units which are competent in field entomology. The plan must detail surveillance sites, define responsibilities, and list mosquito control activities for the detection of a new IMS, importation of MBD, or an autochthonous MBD outbreak. If a national plan does not exist, local plans should be developed as soon as possible (preferably before IMS introduction – and based on this guideline document) for all areas that are considered at high risk for IMS introduction. Regional/provincial plans must include:

- Identification of resources and responsibilities in the organisation of the surveillance activities in sites identified as at high risk for the introduction of IMS, applying (but also customising) the mosquito collection methods and strategies defined at the national level.
- Identification of critical issues and possible preventive measures that apply to the general management of sites under surveillance and/or the draft version of international regulatory measures. These measures should be proposed to the national level and eventually discussed at the international level.
- Detection of a newly introduced IMS and subsequent rapid actions, e.g. mapping and delimitating the colonised area, extension of surveillance activities beyond the first detection site in order to define the extent of the colonised area, and targeting the implementation of mosquito control measures.
- Collection of biological parameter data for IMS to assess sanitary risk in the local context, e.g. breeding sites typology, characterisation of biting behaviour, screening for pathogens in field-collected mosquitoes. This information is essential to optimise the surveillance activities as well as the IMS and MBD control programmes.
- If IMS are introduced, identification of the possible pathways of introduction and the geographic origin of the IMS population by collating information from different areas and surveillance programmes (from local to global). This information is essential for developing/optimising surveillance plans in other areas.
- Investigating the possibilities of spread and passive dispersal from newly colonised sites (trade of goods and movement of people, international/national connections) and informing the appropriate authorities if there is reliable evidence of a risk of introduction to new sites.

Cooperation with the local authorities (local public health units, municipalities) should be established while setting up IMS and MBD prevention/control plans in order to make better use of local expertise and capacity and to ensure that the programme is customised to the specific local situation.

Local level

Local public health units and municipalities with sites at risk for IMS and MBD introduction should be involved in the surveillance and control activities by providing logistic support:

- Providing resources and cooperation in the implementation and management of surveillance activities.
- Providing resources and cooperation in vector control activities in the event of detection of a newly introduced IMS, an imported MBD case, or an autochthonous MBD outbreak.
- Assistance in the dissemination of information to local residents in order to obtain community participation while avoiding local possible conflicts.
- Assistance with evaluation and feedback on the implementation of surveillance activities and control measures.

In countries without capacity at the local level, the regional/provincial stakeholders will have to be responsible for implementing surveillance and control measures.

Examples of organisation and management of surveillance of IMS in continental Europe

France: A first plan for IMS was implemented from 2000 to 2005 through the coordination of regional/provincial mosquito control agencies, at the request of, and funded by, the MoH. The plan became a national plan in 2006, since chikungunya and dengue proved to be an established risk in Europe and prevention of both diseases was imperative. The national plan is coordinated by the MoH, involving regional/provincial public health units and mosquito control agencies that perform surveillance and carry out mosquito control activities on introduction sites of IMS and around introduced MBD cases and local outbreaks. The French plan describes six risk levels and the actions to be taken for each level, including IMS surveillance and control measures. Responsibility is shared between State (MoH) and local stakeholders, according to the risk level: surveillance and control is funded by the local authorities (départements) for areas where *Ae. albopictus* is established, and by MoH for all other areas and PoE. Immediate control measures have led to the successful elimination of IMS from several isolated introduction sites.

National plan: http://www.circulaires.gouv.fr/pdf/2010/05/cir_31164.pdf

Example of local plan (département du Var):
http://www.ars.paca.sante.fr/fileadmin/PACA/Site_Ars_Paca/Votre_Sante/Veille_sanitaire/Maladies_infectieuses/Chikungunya_et_Dengue/plan_de_lutte_anti_vectorielle/LAV_2011_plan_departemental_definitif.pdf

Italy: While several regions and groups of municipalities have had IMS surveillance and control programmes in place for years, the national level (MoH) supported only the centralisation of data on presence and bionomics of IMS (Istituto Superiore di Sanità, ISS). After an outbreak of chikungunya in north-eastern Italy in 2007, the region of Emilia-Romagna developed a prevention plan for dengue and chikungunya, with risk levels and associated surveillance and control measures (funded by the Region Emilia-Romagna) similar to the French plan. At present,

the government (MoH, ISS) provides guidelines/methods to be shared with the regional/provincial levels, promoting the development of similar risk plans in other regions.

Examples of regional plans:

http://www.saluter.it/documentazione/piani-e-programmi/chik_dengue_westnile_2011.pdf;

http://www.veterinarialimenti.marche.it/template/1/allegati/zanzara_tigre_presentazione_piano_monitoraggio_2010.pdf; http://www.epicentro.iss.it/regioni/veneto/pdf/piano_chiku_Veneto.pdf;

http://www.epicentro.iss.it/problemi/zanzara/pdf/zanztigre_piano08-Umbria.pdf.

Spain: The current national plan is limited to surveillance of some points of entry. It is organised by a research institute, with support of MoH and the ministry of defence (to survey military bases). In addition, local mosquito control agencies (for groups of municipalities) develop surveillance and control programmes (two programmes in Catalonia). The government of Catalonia is promoting the elaboration of a prevention plan for the Asian tiger mosquito, but sanitary survey and prevention measures have yet to be defined.

Information websites:

http://www.elbaixllobregat.net/mosquitigre/index.asp

http://www.serveicontrolmosquits.blogspot.com/

Regional plan:

http://www.higieneambiental.com/sites/default/files/images/pdf/Estrategia_mosquit_tigre_2011.pdf

Switzerland: The southern part of the country is colonised by *Ae. albopictus* due to continuous passive and active introduction from neighbouring Italy. Surveillance and control activities have been organised in Ticino canton since 2001, with the aim of eliminating new foci while they are still limited in size. These measures have been successful for several years, but the species is now established in border communities, where IMS are now surveyed and controlled with the aim of reducing risk of MBD transmission. A national plan for the control of *Ae. albopictus* is defined, but its implementation is the responsibility of the regional level (cantons). It will be soon extended to *Ae. japonicus* which is widely established in the northern part of the country.

Plan available from:
http://www.bafu.admin.ch/dokumentation/medieninformation/00962/index.html?lang=de&msg-id=39942

More detailed information about the aims of surveillance according to each scenario

Scenario 1: No established IMS (but with risk of introduction and establishment)

The foremost aim of the surveillance is to detect possible foci of introduction and establishment of IMS at an early stage in order to support the rapid implementation of control measures designed to eliminate IMS.

Surveillance can be extended to areas which report MBD import or transmission in order to detect IMS if present and, if detected, support control measures and pathogen assessment in field-collected mosquitoes.

Scenario 2: Locally established IMS (with low risk of spreading into new areas)

The foremost aim of the surveillance is to quantify any establishment and detect the possible spread of IMS, in order to support the implementation of control measures designed to eliminate the population.

Additional surveillance may assess the quality/efficiency of applied mosquito control measures, detect IMS populations around imported MBD or locally transmitted cases and, if detected, support pathogen assessment in field-collected mosquitoes.

Scenario 3: Widely established IMS (with high risk of spreading into new areas)

The foremost aim of the surveillance is to assess IMS population abundance and seasonal dynamics in order to support MBD risk assessment and control measures designed to suppress IMS.

In addition, surveillance is maintained at PoE, extended to the vicinity of colonised areas and MBD transmission areas, as in previous scenarios, to support control measures aimed at the elimination of IMS from new foci.

Occasional surveillance aims at supporting quality/efficiency checks of control measures and search for pathogens, particularly during outbreaks.

Annex 3: Detailed procedures for surveying IMS

1 Methods of collection of IMS

Larval sampling

Why collect larvae?

Surveillance by larval survey is of key importance as all mosquito species rely on water bodies for their development, thus allowing a focused and rapid inspection (sampling and identification can be performed on the same day), thus offering an optimal cost-benefit ratio. Currently the risk of IMS introduction, as identified in Chapter 2.3, is mainly restricted to container-breeding species which can exploit artificial containers, which widely available in inhabited areas. Recent introductions are mainly passive and related to human activities, mostly in urban areas.

Where to collect?

Larval investigation in urbanised areas must be focused on man-made water bodies (see Table B below) found on both public and private property, below and above ground level, such as discharged containers of different shapes and volumes, flower vases and flower pot dishes in gardens and cemeteries, used tyres left outdoors, rain water barrels, road drains, pits, as well as road beds and potholes for *Ae. koreicus*. Natural containers like tree holes or rock pools may also be inspected in some situations. Other stagnant water bodies (ditches and ponds with vegetation, forest ditch, fen, flooded meadows or forest) are never colonised by invasive *Aedes* species.

When to collect?

The best season for larval detection depends on the latitude and altitude, as well as the mosquito species. In general, mosquito larvae develop when water temperatures are above 10 °C. For optimal development and growth, water temperatures must range between 25 and 30 °C. In spring, the blossoming of dandelions indicates the start of mosquito activity season, and the first frost of autumn may indicate its end. Even if IMS larvae survive in the water, they have very little chance of emerging as adults. The precise start and end of surveillance needs to be adjusted to the particular IMS, considering specific IMS tolerances (temperature, diapause).

Some tips

When surveying a large stock of used tyres it is suggested to search in the shadow of trees, as shady spots are more frequently colonised. When sampling road drains it is suggested to wait a few minutes after the opening of the cover before sampling in order to allow the re-surfacing of larvae disturbed by the operation.

Methods for collecting

Mosquito juveniles (larvae and pupae) can be collected from potential larval breeding sites by netting, dipping, or sucking, depending on the size of the inspected container. Large containers (surface > 0.5 m²) can be checked with an aquatic net (maximum diameter 20 cm, 500µ mesh), a classic dipper, or a one-litre-plastic tray. Smaller containers can be checked with a smaller net (aquarium water nets are usually fine), a kitchen ladle, or a small white plastic cup (Figure A). Very small containers can be emptied into a plastic tray; if not removable (like tree holes), water can be sucked out with a silicon tube or a mucus vacuum.

Collected water samples can be inspected better for the presence of juveniles when decanted in a white plastic tray (1 litre). Larvae are then easily collected with a plastic pipette and put in a fully labelled vial with water (for rearing the larvae to 4th instar (L4) or to adults) or directly in 70% ethanol for later species determination. Rearing larvae to L4 is recommended for more reliable identification (see Chapter 2.2).

Figure A: Larval sampling and adult trapping

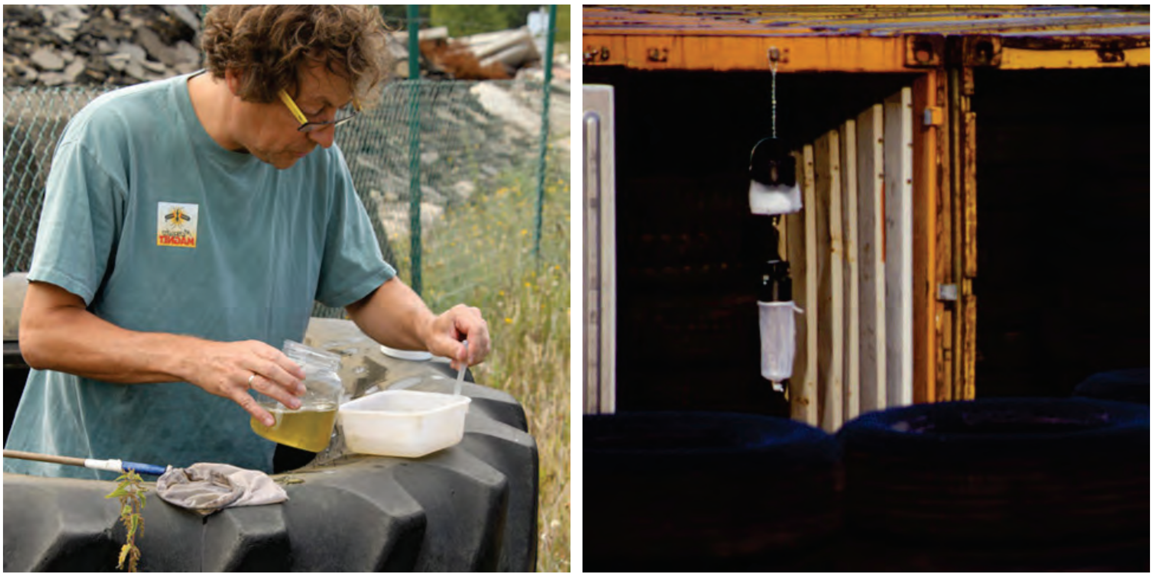

Left: Larval sampling, Belgium 2010. Right: Adult trapping, France 2000

Photos: R. Foussadier (left); F. Schaffner (right)

Adult sampling/trapping

Methods for collecting

Mosquito adult females can be caught by aspirating from resting sites (indoor in shelters, or outdoor in the vegetation) or on hosts (animal or human), and by netting in the vegetation (well-suited for dense mosquito populations).

Human landing collection (HLC) can also be performed for estimating 'landing rates', as mosquitoes must not be allowed to take their blood meal, and must be used only if there is no evident risk of transmission. It is sometimes, however, the most direct and rapid way for estimating a nuisance and can be performed in 15 minutes, for example when visiting a site to check for larvae or inspect adult traps.

Surveillance by adult collection may be best carried out by using effective traps. A number of mosquito traps have been developed which exploit the mosquito's preferences in odour cues, colours, light, host characteristics, or behaviour. As for invasive mosquitoes, the most efficient traps are suction traps baited with chemical lures and/or carbon dioxide (usually from dry ice; light is not attractive at all) (Figure A), and traps that attract females seeking a place to oviposit. Traps targeted to collect females may be useful not only to detect/identify the invasive species but also to obtain samples to be submitted to analysis for pathogen screening.

In summary, the use of traps is highly recommended, but animal baits (if specificity is needed) and HLC are also an option if carried out with the necessary caution.

Note: When not performing HLC, people who sample adult mosquitoes in the field should wear protective clothing (long trousers and sleeves), gloves and netting protection of the neck or the whole head (mosquito head net), as mosquitoes may be aggressive during site inspection.

Where and when to collect

Traps may be positioned in the sites under surveillance (cf. Chapter 1.3 and Subchapter C below) and operated periodically or continuously to cover the whole sampling season; samples are collected during regular inspections. The choice of the most appropriate trap will depend on the most probable mosquito species to be found, the environmental conditions under which the trap will operate, and the availability of skilled technicians and resources (laboratory/domestic animals, dry ice, etc.).

Egg sampling/trapping

Eggs of invasive *Aedes* spp. are usually deposited on the insides of containers, just above the water level. Thus, they can be collected by applying strong white paper tape to those surfaces. This method is particularly useful when checking for introduced eggs in containers of freshly imported goods. All in all, eggs are easier to find than flying adults and offer the added benefit of early detection.

Surveillance of IMS adult presence and activity can efficiently be based on ovitraps that attract adults which lay their eggs on the provided oviposition supports. In this case, only eggs are collected, but if enough traps are used

(see Chapter 2.3 and Annex 5) to cover the area, one may interpolate the size of the adult population. Ovitraps are cheap and easy to handle, but identification of eggs can be difficult and time-consuming (see Chapter 2.3). To complicate matters, the correlation between number of eggs and female density is not always obvious, as females do not lay all their eggs in a single vessel, particularly if there are other options like used tyres.

2 Different mosquito and egg collection methods: strengths and weaknesses

Human landing collection (HLC)

This is probably the oldest and simplest method of collecting host-seeking mosquito females. It catches anthropophilic species most efficiently when performed in shaded environments during the peak of the day. Disadvantages include high cost (manpower, particularly if HLC is performed after hours, e.g. at dusk) and the risk of catching a disease if there is pathogen circulation, even though it is recommended to collect the females before they bite. For standard comparison, a sampling duration of 15 minutes is recommended.

Dry ice/carbon dioxide cylinder-baited suction traps

This type of CO_2-baited suction trap operates with dry ice (producing carbon dioxide through sublimation) or a carbon dioxide tank. CO_2 acts a highly effective mosquito attractant for some species of host-seeking females. These suction traps require battery or AC power to generate electricity in order to run the intake fans. These traps may run with or without a light source. They are not recommended for species such as *Ae. aegypti* , *Ae. albopictus* and *Ae. japonicus*, which are not strongly attracted by CO_2. They have the advantage of not being significantly influenced by background light in urbanised areas, while the main disadvantage is the need to have access to dry ice or carbon dioxide supplies. They also collect non-mosquito haematophagous insects such as Phlebotominae (sand flies), Simuliidae (black flies), and haematophagous Ceratopogonidae (biting midges). These traps usually have an operational time limited by their CO_2 supply (one trapping day/night with dry ice) or battery capacity (12V, 10A, maximum 48 hours).

Mosquito Magnet (MM) traps

Mosquito Magnet traps are CO_2-baited suction traps that produce CO_2 by burning butane. They use excess heat from the combustion process to generate electricity in order to run the intake fans, they can thus be used in remote locations without additional power supply and last up to three weeks. Chemical attractants such as Lurex (L-lactic acid) and octenol (1-octen-3-ol) can be added. These traps are not known to be highly attractive to IMS. Being large, heavy and relatively expensive, they may not be suitable for large-scale studies.

BG-Sentinel trap and Biogents Mosquitaire

These traps have been designed to be attractive to *Ae. aegypti* and *Ae. albopictus*, as they are baited with a specific chemical lure (BG-Lure or Sweetscent). Their effectiveness can be increased by putting a host (e.g. a mouse in a cage) in the trap or by the addition of a carbon dioxide source (Biogents device). With carbon dioxide, the trap is attractive to a wide range of mosquito species. They may operate continuously when a power source is available, but can also run on a 12V battery. They may be used in urbanised as well as rural areas. The traps have also been reported to catch male, gravid and bloodfed *Ae. albopictus*.

Light traps

Light traps operate at night and are most efficient when there are no other lights nearby. They are therefore not well-suited for catching diurnal mosquitoes in urbanised areas and are not particularly attractive to IMS. They also have a large by-catch of other insect species.

Gravid traps

These traps consist of standing water in a black bucket and are designed to collect gravid females as they search for oviposition sites. They are used in arbovirus surveys, as gravid females have taken at least one blood meal and are therefore possibly infected, are more effective indicators of pathogen activity than host seeking females which may have not taken any blood meal. Gravid traps are not considered to be particularly attractive to *Aedes* mosquitoes, but their attractiveness can be improved by using infusions of old oak leaves or grass (bluegrass *Poa* sp.) that have proved to be attractive to *Ae. japonicus*, and, to a lesser extent, to *Ae. albopictus* and *Ae. triseriatus*).

Sticky traps

Like the ovitraps and gravid traps, sticky traps attract gravid egg-laying females which land on internal surfaces and stick to them. It is therefore possible to determine the species and use them for pathogen screening. When

used in high numbers, these traps also reduce the adult population, especially when adults are not numerous and only few alternative breeding vessels are available. Sticky traps are thought to be effective for all container-breeding *Aedes* species, although this has not been tested for all species considered in this guidance document.

Ovitraps

Ovitraps are very simple and considered effective for container-breeding *Aedes* species. They consist of a small black plastic bucket with an overflow hole (0.3 to 1.0 litre), filled with water to two thirds so eggs can be flooded, and an oviposition support (usually germination paper, a wooden stick, or a piece of polystyrene). The size of the bucket has to be adjusted to the trap-checking frequency and local rainfall in order to prevent the trap from drying out. If the trap-checking interval exceeds eight days, it is recommended to add some long-lasting insecticide (biopesticide or insect growth regulator) in the water to stop the trap from becoming a breeding vessel. Ovitraps can be positioned close to or under vegetation or near buildings and should be labelled, e.g. with 'Ongoing scientific study. Please do not remove! Contact…' (Figure B).

Although ovitraps are cheap and easy to use, identification of eggs is difficult and time-consuming, and the correlation between number of eggs and female density is not always obvious, as females do not lay all their eggs in a single vessel, particularly if there are other options like used tyres. This can be improved by modifying the number of traps (see last paragraph of this Annex).

For detecting the presence of an IMS, it is recommended to use three to five ovitraps simultaneously in one place (i.e. a parking lot or a district of a city) in order to increase the sensitivity of the trapping. Traps can also be placed in different micro-environmental conditions (e.g. south or north side of a building, under vegetation, in a less obstructed area, or near various putative hosts).

If several breeding sites are available, ovitrap efficiency is reduced, but their attractiveness can be improved by using oak leaves or grass infusion (as for gravid traps). Ovitraps can be a useful tool for surveying remote locations with low risk of introduction, as they can be checked infrequently (several weeks to several months). In high-risk areas they should be checked more frequently, and can be used in conjunction with larval searches in other containers. Ovitraps are also useful to assess the extent of colonisation if an invasive species is already known to be present. In this case it is no longer necessary to systematically identify the eggs; it is sufficient to identify new positive sites and conduct a random selection of samples.

Figure B: Ovitraps and oviposition support

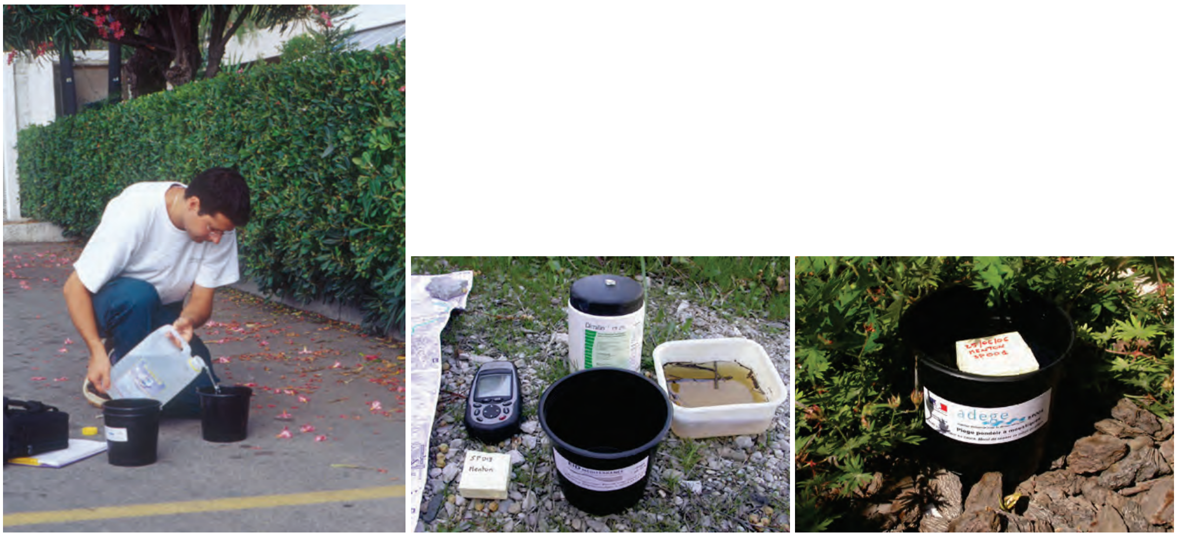

Above: Ovitrap set-up and positioning

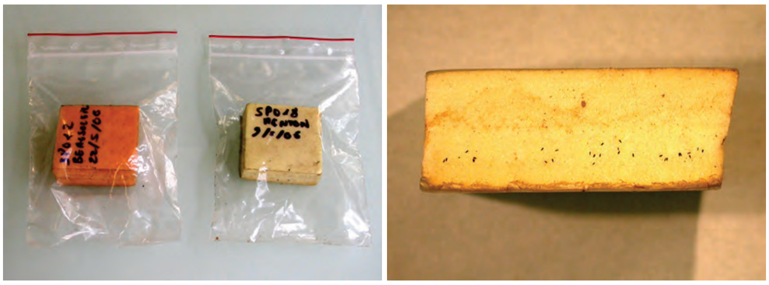

Above: Oviposition support (e.g. polystyrene)

Photos: F. Schaffner (France, 2004)

Table A: Most commonly used traps for adult mosquitoes: specifications, strengths and weaknesses

Trap model	Attractants	Targeted mosquitoes	Main species attracted	Strengths	Weaknesses	Remarks
**BG-Sentinel**	• Lure (mixture of human skin odours: BG-Lure, Sweetscent)<br>• Carbon dioxide (optional)	Host-seeking females	• *Ae. aegypti*<br>• *Ae. albopictus* (with carbon dioxide: almost all mammophilic spp.)	• Very efficient in trapping IMS<br>• Relatively small and light<br>• Manageable in the field<br>Efficient lures	• 12V battery or power source needed<br>• Easy to damage battery<br>• Prone to be stolen	• Probably most commonly used adult trap for IMS<br>• Has been shown to attract male and gravid or bloodfed female *Ae. albopictus*
**Miniature CDC (without light)**	Carbon dioxide (dry ice)	Host-seeking females	Almost all mammophilic spp.	• Small, easy to manage<br>• Light can be disconnected<br>• Some models have a photo switch system	• Not very efficient for IMS<br>• Needs a source of carbon dioxide	When using light many other insects are trapped (e.g. moths), mixing up the sample, thus it should be switched off.
**Mosquito Magnet**	• Carbon dioxide (from propane)<br>• Lure (octenol)	Host-seeking females	Almost all mammophilic spp.	No power supply needed, runs for three weeks.	• Heavy, large, and expensive<br>• Not highly efficient for *Ae. albopictus*	
**Gravid trap**	Infusion	Oviposition-seeking females	Mainly *Culex*	Used for pathogen screening in mosquitoes, as these traps attract females that have taken at least one blood meal (more chances to be infected).	Standard gravid trap does not attract IMS	• Method of preparing infusion can influence attractiveness<br>• Gravid mosquitoes are often used in surveillance for arboviruses
	Oak leaves or grass infusion	Oviposition-seeking females	• *Ae. japonicus*<br>• *Ae. albopictus*<br>• *Ae. triseriatus*		Efficiency improved compared to standard but still limited	
**Sticky trap**	Infusion, water, lure (AtrAedes)	Oviposition-seeking females	• *Ae. aegypti*<br>• *Ae. albopictus*	No power supply needed, relatively easy species determination	Low catch rates	Used in the estimation of population size and calculation of dengue risk.

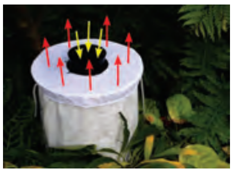
BG-Sentinel trap

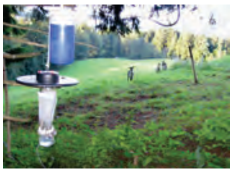
CDC trap with dry-ice container

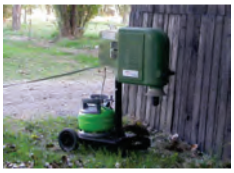
Mosquito Magnet trap

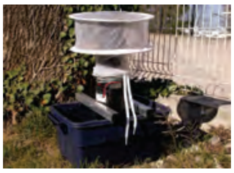
Gravid trap

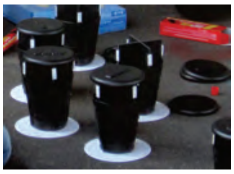
Sticky trap

Photo left: A. Rose, Biogents; all other photos: F. Schaffner (France, 2004)

3 Surveying sites at risk for introduction and inspecting areas at risk for spread of IMS: detailed procedures

In order to optimise resources and increase the effectiveness of surveillance activities, the sites considered to be at risk must be identified and ranked according to priority.

Maps depicting the risk of establishment of mosquito populations may be helpful when defining surveillance strategies, but the final choice on which points of entry (PoE) and which possible spreading areas will be surveyed must be made by local teams who can customise the regional strategy to the local context. Thus, before initiating surveillance, stakeholders at the national level should identify local expertise in entomology, public health, and mosquito control. If no such information is available, contact points listed in this report can be used. National/international experts in entomology, surveillance, pesticide science, or pest control can also provide useful input and advice and can help to provide training. At least one expert in medical entomology should be involved from the beginning of the surveillance planning.

Risk maps were produced for *Ae. albopictus* at the European level (ECDC 2009). Similar country-level maps at a higher resolution (1 km resolution instead of 5 km) would be useful. Factors driving the risk of introduction, the establishment and the spread of the target species, such as land use and sites/areas favourable to introduction (e.g. used tyre importers, main highway rest areas) might then be added to the maps as overlays. The result would be a suitability map which would be helpful when prioritising survey and samples sites.

Identification of sites at risk for introduction of IMS (cf. Table 4)

Used tyre trade

Stacks of imported used tyres are the primary points of entry (PoE) for IMS. Used tyres are often stored outdoors and therefore collect rain water which may persist for some time, providing a suitable breeding place for IMS and a few indigenous mosquitoes (Figure C). When stored for a long time, accumulation of dead leaves in the rain water renders this micro-environment even more suitable for mosquitoes which evolved to breed in tree holes and phytotelms.

Used tyres may provide suitable habitats for eggs and larvae survival during transit, provided the tyres contain a sufficient amount of water. Large numbers of mosquitoes may therefore be transported in a single container (Figure C). Only used tyres that are stored in open areas before transportation are a risk, and not those protected from rain, retread or new (tyres in the last two categories are never stored in the open, as sunlight damages the tyre gum).

The levels of risk posed depend on the type of trade:

- Intercontinental trade: Most of the car tyres used in Europe are produced in Europe. Used tyres with specific/rare sizes and high commercial value (i.e. airplane, subway, military and construction vehicles, trucks, etc.) are more often imported. Unfortunately, such tyres, because of their high cost, are designed for recycling (tyre retreading), and because they are used, damaged or large, are often stored outdoors.
- The names of companies that deal in used tyres may be learned from national customs services or professional trade associations. The main countries that import used tyres to Europe are Japan, the USA, and Korea. The risk of IMS introduction is directly related to the geographical origin of the tyres: all tyres imported from countries where IMS are indigenous or established (e.g. southeast Asia, USA) represent a high risk. Some countries passed regulations dealing with the used tyre trade and tyre storage (e.g. Brazil: Measures affecting imports of retreaded tyres, WT/DS332); these regulations should be applied to high risk imports. In 2006, the French ministry in charge of environment, via the local state representatives, requested major used tyre importers, which were considered potential PoE, to schedule and implement measures to prevent the spread of mosquitoes and to inform the authorities about implemented measures and met deadlines.
- Pan-European trade: Also very active, Pan-European trade mainly concerns tyres that are manufactured and used within Europe. Thus, all types of used tyres imported from EU countries where IMS are established must be considered a risk, i.e. first and foremost tyres from Italy, but recently also France, Spain and the Balkan countries (see VBORNET IMS distribution maps, www.vbornet.eu). However, some EU countries (i.e. Belgium, Netherlands, UK) may also export tyres previously imported through intercontinental trade, and those tyres also pose a risk.
- National trade: This trade poses a risk only in countries where IMS are established (see above); in this case, all used tyres may be considered a risk, including those that were locally produced and used. In the case of abandoned used tyres storage sites, there is only a risk if IMS are established in the vicinity.
- Export trade: In countries where IMS are established and companies trade in used tyres, it is recommended that all exported stacks of used tyres are surveyed and treated to eradicate IMS, similar to the recommendations given for airports (IHR 2005). Alternatively, companies may be asked to supply destination details so that surveillance authorities in the importing countries can be informed. A large proportion of European used car tyres are exported to Africa, which poses the threat of further introduction of IMS to Africa if IMS should become well-established in Europe.

Surveillance procedures

When starting a surveillance programme it is necessary to assign priority categories for site inspections at used tyre storage sites, due to their large number. Global data on the amount of used tyres imported to a country can be obtained from the United Nations Commodity Trade Statistics Database (http://comtrade.un.org/db/) by searching the commodity category ‘Pneumatic tyres used’ (#401220). Once a list of companies has been established (see above), a first step would be to rank the companies according to trade volume and origin of tyres (see Box A), thus estimating the associate risk level. In a second step, the company should be contacted to establish (1) if mosquitoes pose a nuisance at the storage site, (2) if tyres have been stored outdoors, and if so, for how long, and (3) if damaged tyres are kept at the site for extended periods. Storage sites with no outdoor storage (this can also be verified with Google Earth or a similar online satellite imagery system) do not need inspection and can be removed from the list of sites to be surveyed. Other information that may help to complete the ranking of companies is set out in Box A.

Box A: Assigning a priority category for inspection to companies that import used tyres

France

The used tyre importation data obtained from the French custom services (nomenclature code 401220) revealed that 214 French companies were importing used tyres in 1999 (with a minimum of 1.0 ton); the tyres originated in 42 countries. To plan site inspections, companies were ranked based on trade volumes and origin of the tyres:

1. Country classification:

1 = Country where *Ae. albopictus* is indigenous or widely established.

2 = Country where *Ae. albopictus* has been observed and is still present.

3 = Country not in above categories but with a high trade volume in used tyres.

4 = Other countries.

2. Company ranking

**a. Countries of origin of tyres**		**Score a**
C1: Country where *Ae. albopictus* is indigenous or widely established.		**10**
C2: Country where Ae. albopictus has been observed and is still present.		**8**
C3: Country not in above categories but with a high trade volume in used tyres.		**6**
C4: Other countries.		**4**
**b. Volume of used tyre trade per country**		**Score b**
> 500 t		**a** x **2.0**
100 to 500 t		**a** x **1.5**
1 to 100 t		**a** x **1.0**
< 1 t		**a** x **0.5**
Maximum value = 20		
**c. Volume of used tyre trade per country category**		**Score c**
If volume > 100 t	C1	**4**
If volume > 100 t	C2	**3**
If volume > 100 t	C3	**2**
If volume > 100 t	C4	**1**
If volume =< 100 t	C1 to C4	**0**
Maximum value = 10		
**Global score of company:**		
b is calculated for each country, c for each category		**Final score**
		**highest b + total c**
Maximum value = 30; minimum value = 2		

This ranking covers a large range, which is useful when there is large number of companies. From 1999 to 2005, 110 companies were surveyed in France (first by phone and then on location if they stored tyres outdoors), which represents 52.6 % of the companies and 95.6 % for the imported volumes. Five sites were found positive for one or more IMS, at one or several times, and all of them were ranked in the 'top 10' of the original ranking, i.e. had been assigned a top-ten priority category for on-site inspection. The remaining companies that were not surveyed imported less than one ton/year, and it was assumed that they had facilities to store tyres indoors. Also not surveyed were companies that imported tyres from countries not identified as a risk (and also importing less than 70 tons annually).

Source: rapports ADEGE, EID Méditerranée, 2000–2006

Box A (continued)

The Netherlands

In cooperation with representatives from the tyre industry, 34 Dutch used tyre companies were identified in 2010. A questionnaire on imported tyres was sent to all 34 companies. In case of non-compliance, a phone interview was conducted. Based on the obtained information, a qualitative risk assessment was performed for each company to assess the risk of introduction of exotic mosquito species, taking into account the following factors: (1) the type of imported tyres, (2) the origin of the tyres, and (3) the storage method. Risk factors were quantified by assigning a score:

Risk factor	Categories	Score
1. Type of tyre	Car/van	**0**
	Truck	**1**
	Airplane	**1**
	Agricultural vehicle	**1**
	A combination of above tyre types	**1**
2. Origin	From EU country without reports of IMS (negative)	**0**
	From EU country reported as positive for IMS	**2**
	From national company reported as positive for IMS	**2**
	From non-European country	**2**
	A combination of above origins	**2**
3. Storage method	Indoors (dry)	**0**
	Outdoors (exposed to rain)	**2**
	A combination of above storage methods	**2**
4. Previous results	Company not positive for IMS in recent years	**0**
	Company positive for IMS in recent years	**5**
5. Site inspection	Inspector's observations suggest a low risk	**1**

Cumulative score	Risk categories		Inspection frequency	Timing of inspection(s)
>5	Cat 1	(very high risk)	Every two weeks	Apr–Oct
5	Cat 2	(high risk)	Every two weeks	Apr–Oct
3–4	Cat 3	(moderate risk)	Three times per season	Jun–Oct
<3	Cat 4	(low risk)	Once per season	Jul–Aug

The total score was calculated for each company as a cumulative score of all risks factors (one value per risk factor). This ranking provided a short ranking range, sufficient if the number of companies is limited. The ranking not only helped with the scheduling of inspections but also definec the frequency of the visits in relation to the risk. In the Netherlands, all 34 identified companies were surveyed based on their ranking (Cat 1 and 2: n=14, Cat 3: n=3; Cat 4: n=17). In 2010, 180 inspections were carried out at the 34 companies (including immediate surroundings). As a result, three IMS were detected on 27 occasions and at five locations.

Source: Beeuwkes et al. 2011

Company staff often claim a rapid turnover of tyres, and thus no risk of IMS introduction. Site inspection (Figure C) is often a necessary third step, as one can find numerous mosquitoes and/or tyres that are not traded frequently (due to uncommon size or damage) forgotten, or recycled for another use, e.g. forming a wall or fence around the storage site.

During a site inspection, employees can be asked about the origin of the tyres and if they experience a mosquito nuisance problem and where it emanates from. When performing site surveillance, tyres should be inspected for immature mosquitoes in a defined order of priority: (1) tyres originating directly from countries at risk, (2) tyres originating from importing and exporting countries, (3) damaged tyres if stored separately for long periods, (4) tyres stored for several months or years, (5) tyres stored under vegetation and in shaded areas, (6) tyres accumulating dead leaves and other organic matter, (7) any remaining tyres. Tyres which seem to have been forgotten offer a perfect micro-environment for IMS that have been introduced and have reproduced on site; these tyres are perfect for surveying the presence and establishment of an IMS. Recently imported tyres may be surveyed to detect recent introductions of IMS. Many larvae hatch from the eggs following heavy rainfall and each time they are moved. Finally, the area surrounding the storage site should be inspected for other potential IMS larval breeding sites, e.g. rain water barrels in gardens, road drains, abandoned containers, and tree holes in adjacent woodland. Given the introduced stage (eggs) and the large number of potential larval breeding sites in stacks of used tyres, inspections may be advantageously carried out by larval search, alongside human landing catches and adult trapping.

Prevention at storage sites for used tyres

To reduce the risk of establishment of IMS, some precautions should be applied to all used tyre storage facilities: (1) immediately recycle/destroy all damaged tyres; (2) store other tyres properly out of water (Figure C); if this is not possible, (3) store the tyres in small and accessible piles for easy surveillance and larval control; and (4) identify tyres which pose the highest risk (see above) and store them as recommended above. Employees at these sites should be alerted that opening an intermodal freight container could release mosquitoes (Figure C). If they observe live mosquitoes flying out of a container, they should immediately alert the person in charge of surveillance. In addition, a single-shot insecticide spray like the ones used in airplanes should be used inside the container as soon as it is opened.

Figure C: Storage sites for imported used tyres

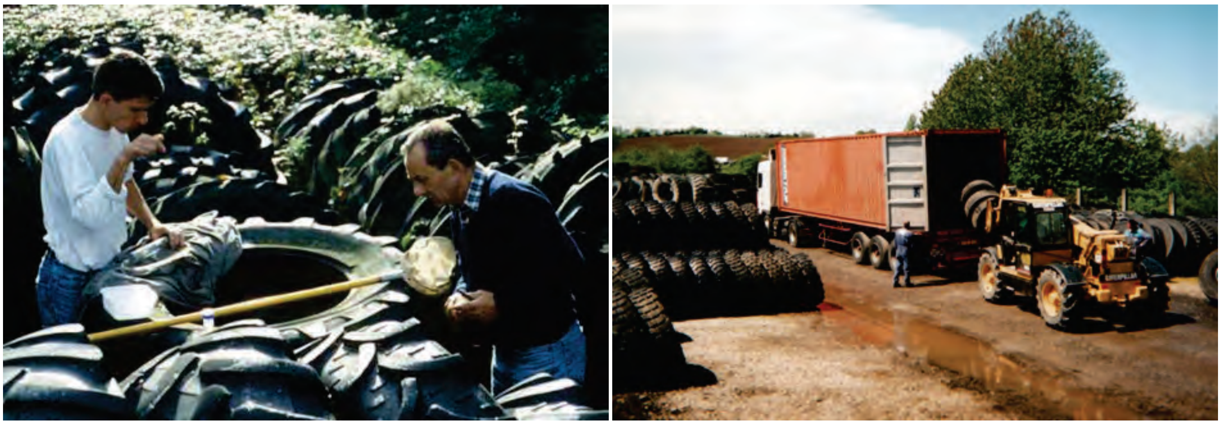

Left: Tyres attractive to IMS; right: Unloading of tyres imported from the USA

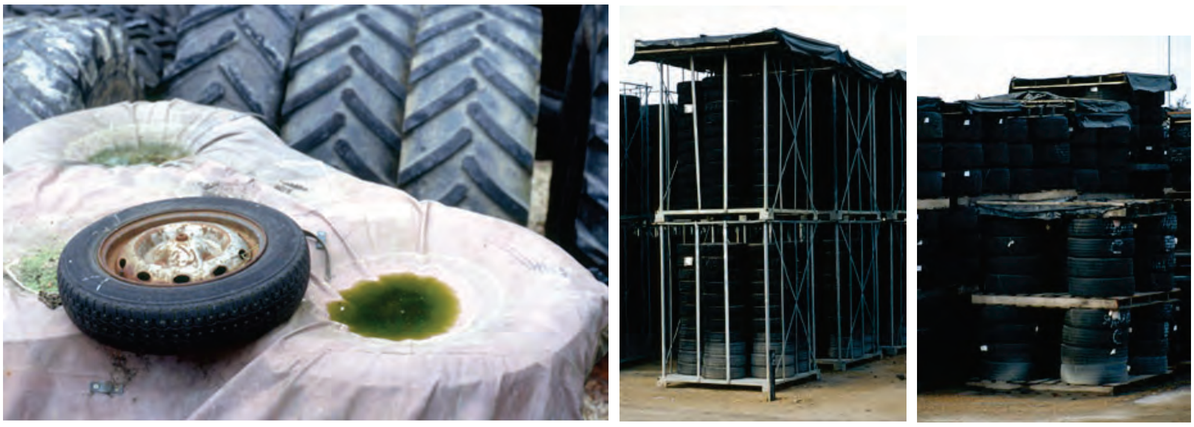

Left: Improper out-of-water tyre storage: pool formation in tarpaulin; right: proper tyre storage, outdoors but out of water

Photos: F. Schaffner (France, 2000)

Lucky Bamboo trade

Ornamental plants (mainly plant cuttings) transported in wet condition may host eggs/larvae/adults of IMS. Once imported, the plants are temporarily kept in greenhouses before delivery, which are highly favourable to IMS indoor breeding, even during cold weather. Plant trade types already proven as possible sources of IMS are 'Lucky Bamboo' (*Dracaena* spp.) (Figure D), imported from southeast Asia (China and Taiwan). Other plants posing a threat are aquarium plants which have been associated with the introduction of exotic aquatic insects. Bulk container shipping presents the greatest threat, as the plants need a substantial water supply to survive for several weeks while onboard the vessel. Small shipments are made by airplane and need no water. Information about plant trade is difficult to obtain from customs services, as they are all subsumed in a category called 'Cuttings and slips, not rooted' (#060210). Global import data for a country can also be obtained from the UN Comtrade database (http://comtrade.un.org/db/). However, there are only a few companies involved in this trade, and most of them are based in the Netherlands where the cuttings are processed and prepared for flower shops. As with used tyres, inspections should focus on larval search, alongside human landing catches and adult trapping.

Other goods trades and heavy equipment transports

Other goods and equipment at risk concern materials that can retain water and are stored outdoors in their country of origin and where IMS are established. Repatriation of construction and military material and vehicles could also be associated with transport of mosquitoes. Indeed, *Ae. albopictus* was suggested as possibly introduced into the USA via military equipment repatriated from Vietnam (Nowell 1996). Also, stone fountains

imported from China to the French Riviera were suspected to be a source of introduction of *Ae. albopictus* (Figure D). Inspections may be advantageously based on larval search, human landing catches and adult trapping.

Figure D: Other goods trades at risk for introduction of IMS

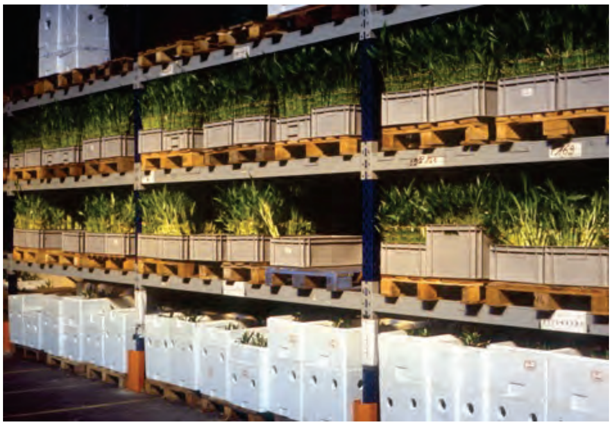

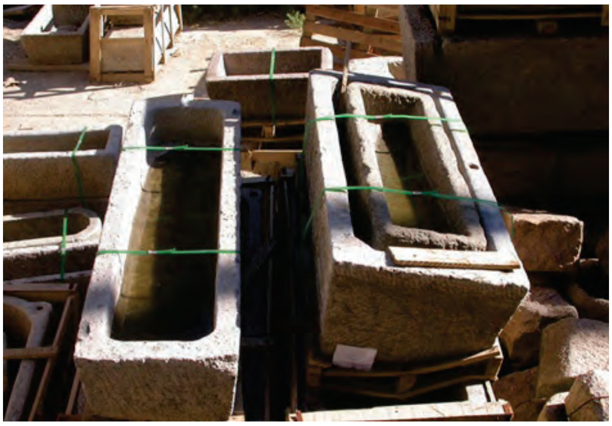

Left: Lucky Bamboo cuttings from Taiwan; right: Stone fountains from the People's Republic of China.

Photos: F. Schaffner (France, 2004, 2006)

Ground traffic

Ground vehicles moving people and goods may inadvertently transport insects, including mosquitoes. Mosquitoes can hide in the cab of a truck and escape during a stop a petrol station or rest area. Due to the high number of vehicles moving every day, the possibility of transporting mosquitoes is significant. Petrol stations and parking lots on highways and main international/national roads should be subject to surveillance. The risk posed by ground transport decreases with the distance from the colonised country/region, as drivers are supposed to stop every two hours. Thus PoE at high risk are located within a two to two and a half hours' drive. A higher risk exists at the first stop/rest facility in a country, as many tourists stop there to buy local products at local prices. The only known introductions of *Ae. albopictus* into Germany were reported from such places (Pluskota et al. 2008; Werner et al. 2012). Other possible PoE near country borders are commercial centres, tourist sites, and accommodation facilities. These sites should be surveyed if high numbers of tourists originate from neighbouring European countries that are infested by IMS. Surveillance activities of PoE related to ground traffic are best carried out with ovitraps providing attractive egg-laying sites for introduced females.

Other points of entry (PoE)

Others PoE may be sea ports, airports and railway stations. Introduction of *Ae. aegypti* into the EU through these PoE was not uncommon in the past, although there were no reports of introductions in recent years. But a high introduction potential remains, and thus the importance of these PoE can be considered as secondary in comparison to other PoE. Vehicles arriving on ferries may carry IMS females, but upon arrival these vehicles usually travel directly to their destinations, without stopping at the port itself. In contrast, vehicles waiting to embark may spend hours in the port area. It is recommended that ports (like airports) are kept 'free of IMS', airports (IHR 2005).

Marinas and their tourist boat traffic are suspected to have enabled the introduction of IMS, for example along the Balkan Adriatic coast. Therefore, marinas in the Mediterranean and the Black Sea basins should be considered for surveillance during the summer season. Commercial ports should also be surveyed, as ships are known to transport IMS (Nie et al. 2004). The risk through intermodal freight containers is negligible because containers are very rarely opened at the ports.

Ports may be surveyed with ovitraps and/or routine adult trapping. It may be possible to evaluate the risk related to these sites by analysing a port's international traffic routes and the volume of goods passing through. Airports may also be surveyed by these methods, both in order to check for possible introductions and to demonstrate that an airport is 'free of IMS', as requested by IHR in the event of an MBD outbreak (IHR 2005). Finally, train stations can be surveyed for IMS, introduced by trains that originate in infested areas, although modern air-conditioned trains are not favourable to mosquito survival. Train terminals that serve so-called 'rolling highways' (RoLa), a combined transport system to transport trucks by rail, could be exposed to similar risks as ports and airports.

Determination of surveillance period (cf. Table 4)

In relation to climatic conditions

The climatic conditions of the region under surveillance must be taken into account when defining the active surveillance period. In more temperate regions, the activity period is shorter (i.e. May–September) than in more Mediterranean-climatic regions (i.e. March–October). In spring, the blossoming of dandelions indicates the start of

mosquito activity season, and the first frost of autumn usually indicates its end. Even if IMS larvae survive in the water during the winter, they have very little chance of emerging as adults. However, start and end of surveillance has to be adapted to the IMS, considering specific IMS tolerances (temperature, diapause), e.g. eggs of *Ae. japonicus* may hatch as soon as temperature is favourable (snow melting), whereas eggs of *Ae. albopictus,* undergoing a true diapause, may not hatch before mid-March, regardless of temperature.

In relation to human activity

Seasonality and import of goods. The import of some goods is non-seasonal (i.e. used tyres), whereas others products show seasonal peaks (i.e. Lucky Bamboo before Chinese New Year). Surveillance may be implemented or reinforced during such peaks.

Peaks in tourism. Introduction by transport is also seasonal (e.g. summer peak in ferry traffic in the Mediterranean region), which may also coincide with favourable climatic conditions. Surveillance of ferry ports should be strengthened during these peak activity periods.

Outbreaks in MBD-endemic areas. Surveillance should also be reinforced at airports when outbreaks of MBD are reported from endemic areas and at airports that show extensive traffic with these areas. Although never reported for arbovirus infections, the introduction of infected adult mosquitoes via airplanes is possible and has become a regular occurrence in conjunction with malaria vectors (Gratz et al. 2000).

Trapping and sampling (cf. Table 4)

Placement of traps

Some behavioural characteristics of exotic mosquitoes can help to determine suitable sites for the placement of mosquito traps:

- Mosquitoes prefer shaded areas for resting that are protected from wind, for example bushes and shrubs.
- Mosquitoes look for small and dark water containers in shaded areas to lay their eggs in.
- Mosquitoes prefer to fly relatively close to the ground, e.g. below three metres.
- Mosquitoes prefer to fly through bushes and shrubs, and avoid open terrain like meadows (Figure F); they prefer to use of ‘shrub-corridors’ (e.g. hedges) to fly from one place to another rather than crossing open terrain.

Suitable sites for placing oviposition and adult traps are thus dense shrubs (Figure E). Traps should be placed out of sight and where children cannot reach them. They should be labelled ‘Ongoing scientific study/surveillance’ and carry the name of the owner and further contact details. It may be necessary to inform municipalities and their gardening staff. If traps are placed on private propriety, permission of the landowner is required. In glasshouses or at storage sites for used tyres (or other company yards), traps should be placed out of the reach of employees (in most cases this means along the site perimeters) and in a shaded area close to shrubs (Figure E).

Figure E: **Proper placement of mosquito traps**

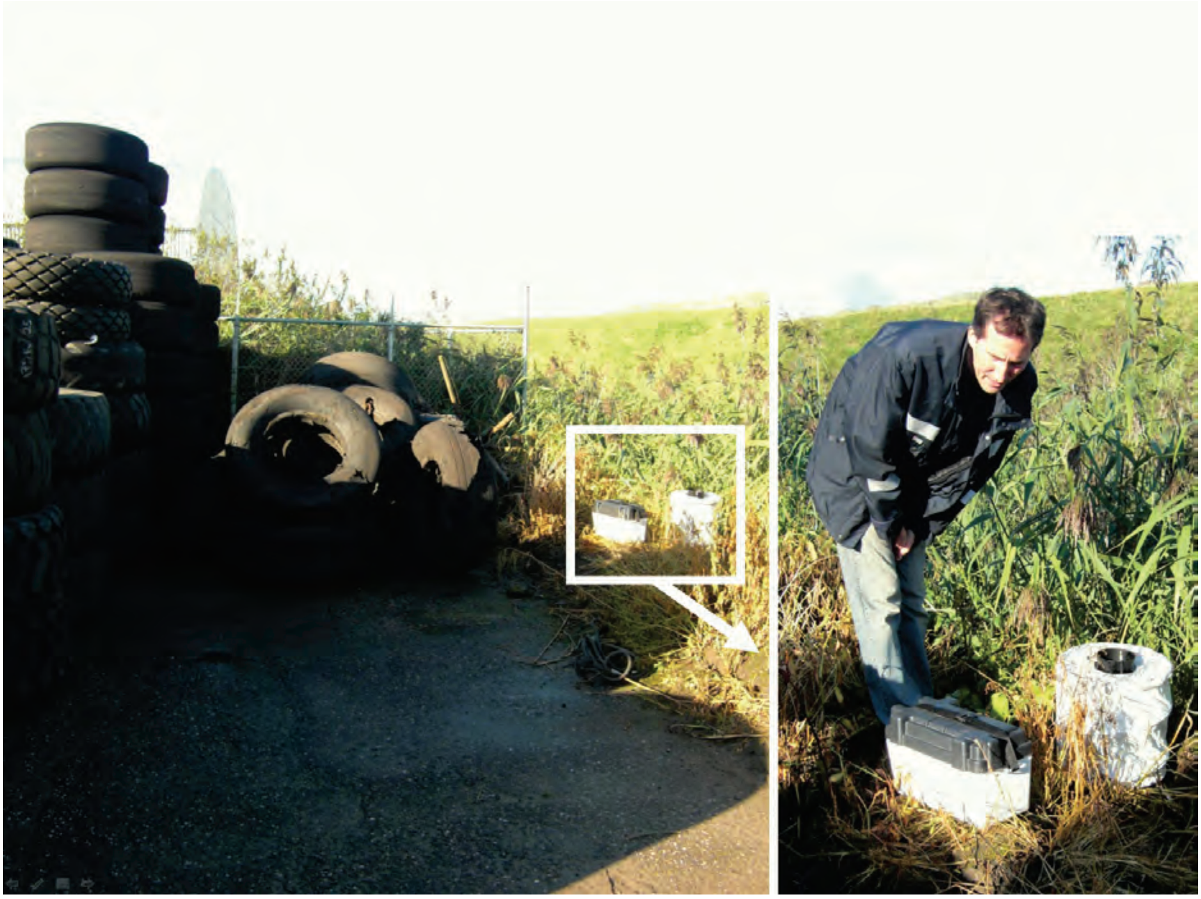

Above: Odour-baited adult trap (BG-Sentinel) at a storage site for used tyres

Photo: M. Le Grand (Dutch Ministry for Public Health, VWS)

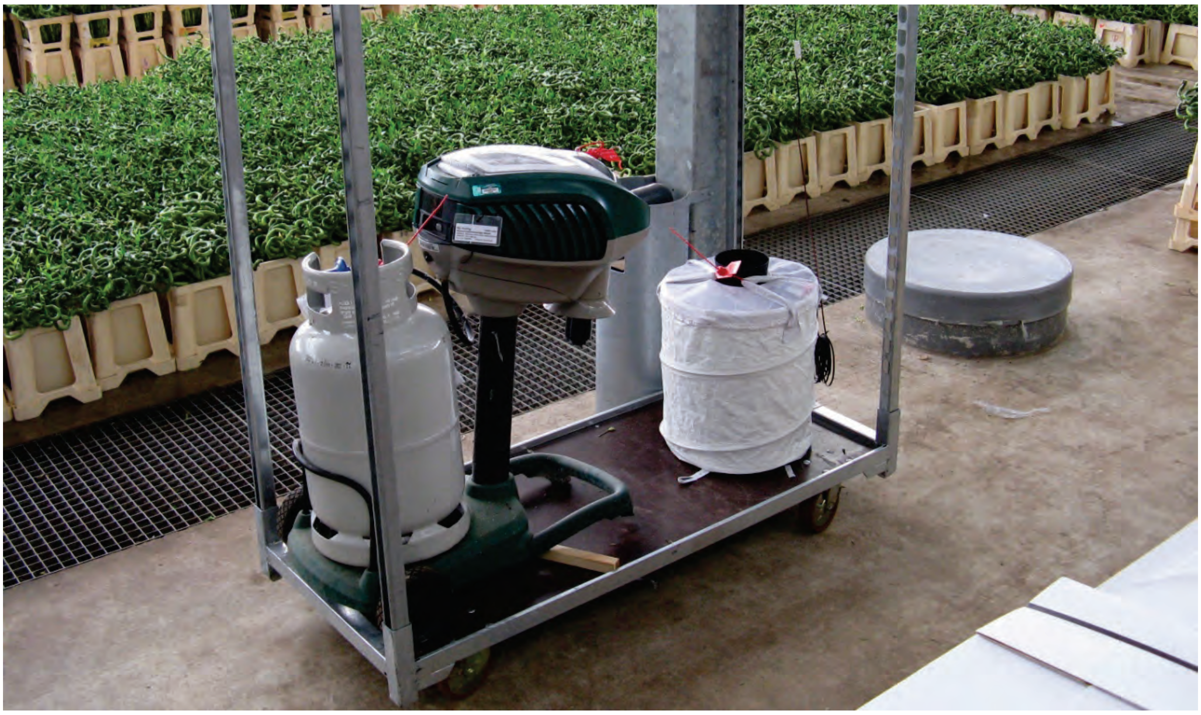

Above: Combined odour- and CO_2-baited adult traps (Mosquito Magnet) at a greenhouse for Lucky Bamboo

Photo: M. Le Grand (Dutch Ministry for Public Health, VWS)

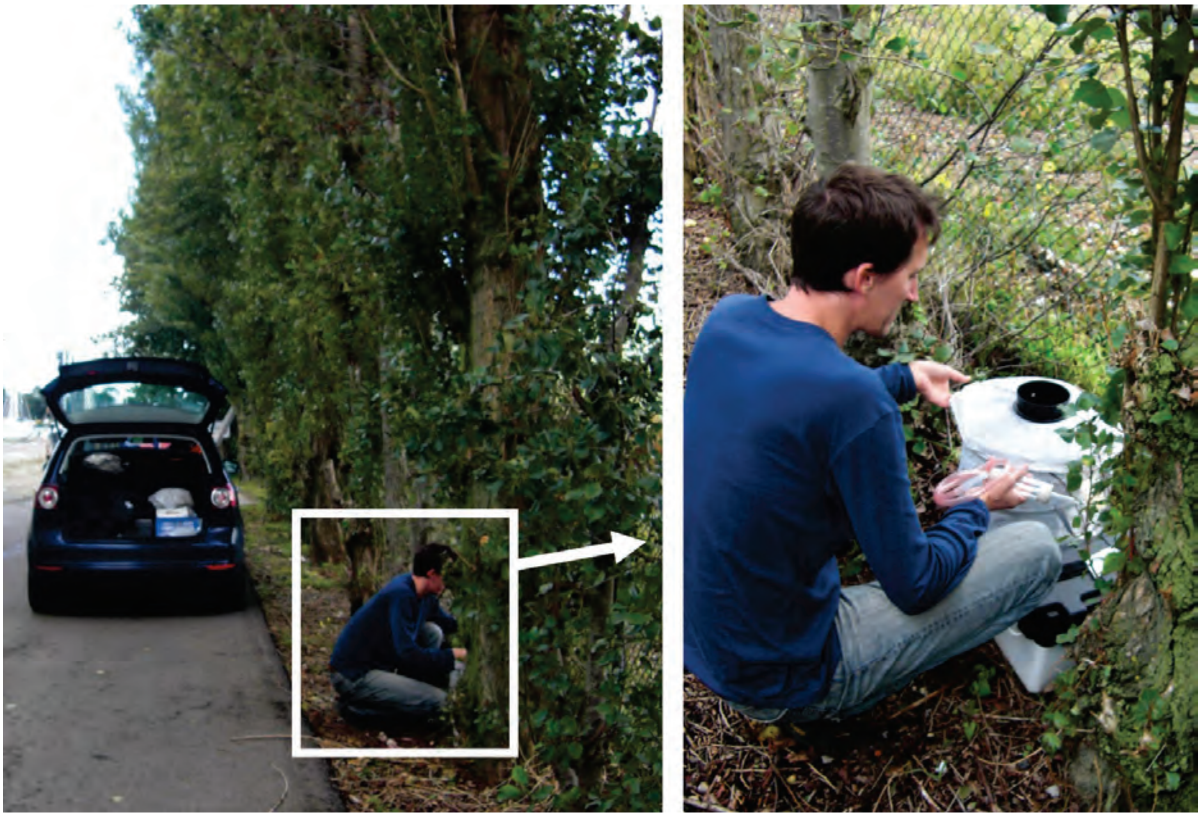

Above: Odour-baited adult trap in shrubs

Photo: M. Le Grand (Dutch Ministry for Public Health, VWS)

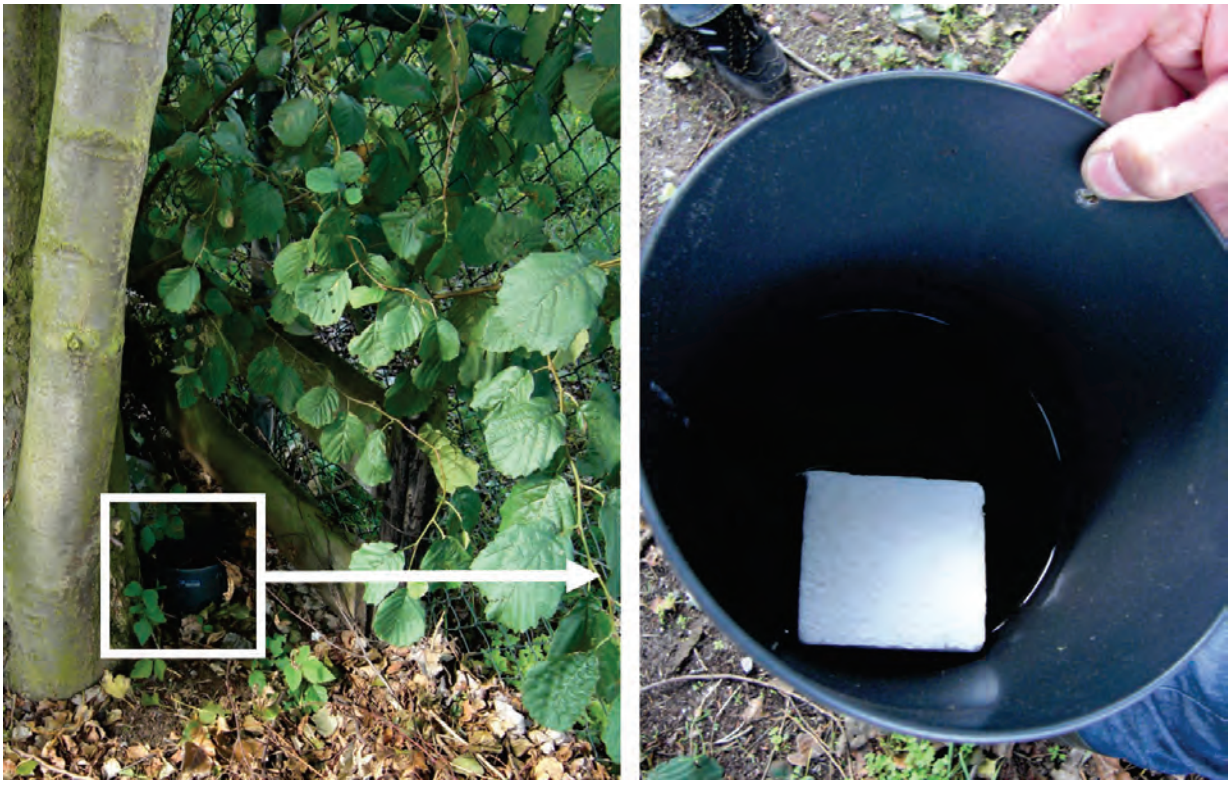

Above: Ovitrap in shrubs

Photo: E.-J. Scholte (Dutch National Centre for Monitoring of Vectors, CMV, NVWA)

Figure F: Unsuitable habitats for IMS

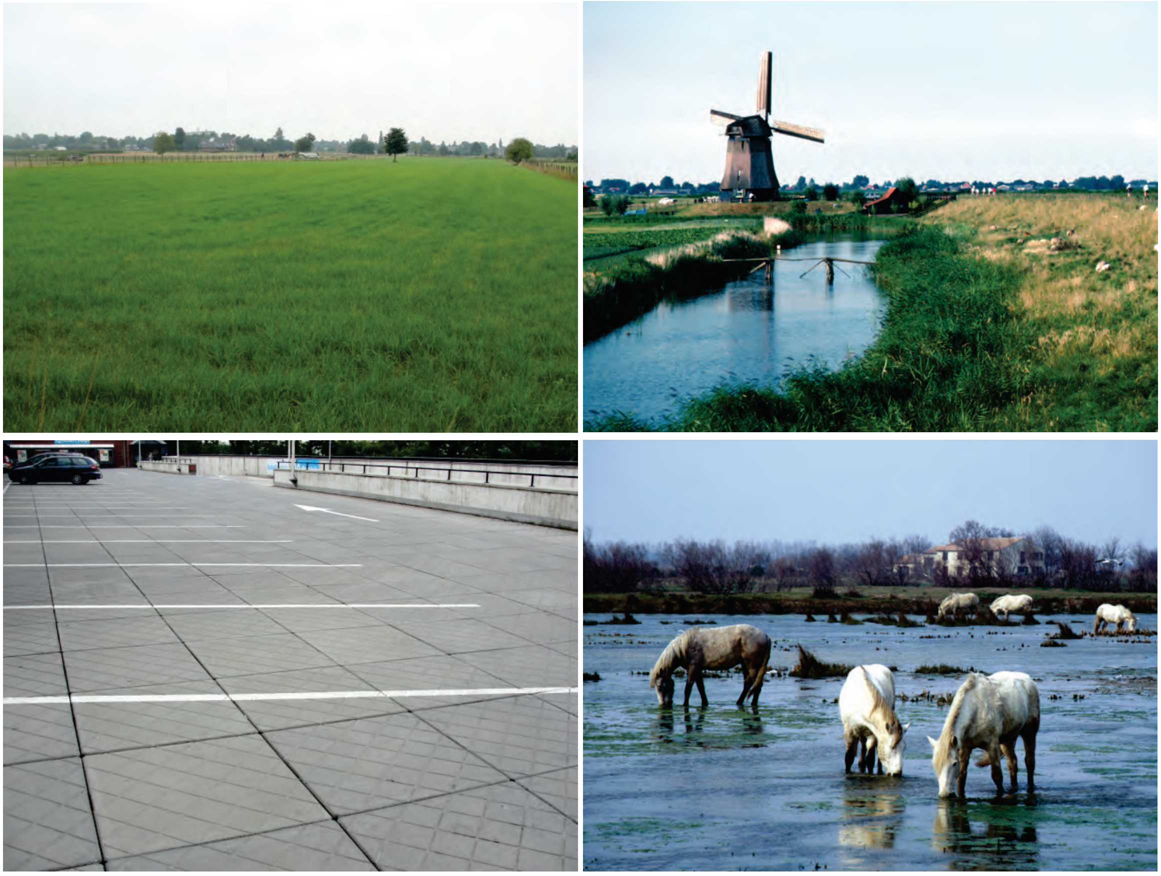

Unsuitable habitats for adult IMS (left); unsuitable habitats for IMS larvae (right)

Photos: F. Schaffner

Potential larval breeding sites to be surveyed

Sampling of immature mosquitoes may focus on small man-made water containers that accumulate dead leaves, algae and/or other organic matter, with some adaptations to the IMS that is surveyed (Figure G, Table B), e.g. *Ae. koreicus* can also breed in road tracks and ditches without vegetation. Tyres appealing to mosquitoes are usually located along the site boundaries. Natural pools of water like tree holes and similar bodies of water may be searched as they are particularly attractive to IMS. Larval habitats unsuitable for IMS include saltwater, pools and ditches with vegetation, large water bodies such as lakes and canals, and rivers (Figure F).

Table B: Larval breeding sites commonly colonised by invasive *Aedes* species

Invasive *Aedes* mosquito species	Diverse recipients	Flower vases and dishes	Used tyres	Rainwater barrels	Unused swimming pools	Road drains	Concrete basins	Unused pits	Pools on tarpaulins (without vegetation)	Tree holes	Rock pools	Road tracks
Ae. aegypti	✓	✓	✓	✓	✓	✓	✓	✓	✓	✓	✓	-
Ae. albopictus	✓	✓	✓	✓	✓	✓	✓	✓	✓	✓	✓	-
Ae. atropalpus	✓	✓	✓	✓	?	?	?	?	✓	✓	✓	-
Ae. japonicus	✓	✓	✓	✓	✓	✓	✓	✓	✓	✓	✓	-
Ae. koreicus	✓	✓	✓	✓	✓	✓	?	?	✓	✓	✓	✓
Ae. triseriatus	✓	✓	✓	✓	?	?	?	?	?	✓	✓	-

Figure G: Containers suitable for breeding of IMS

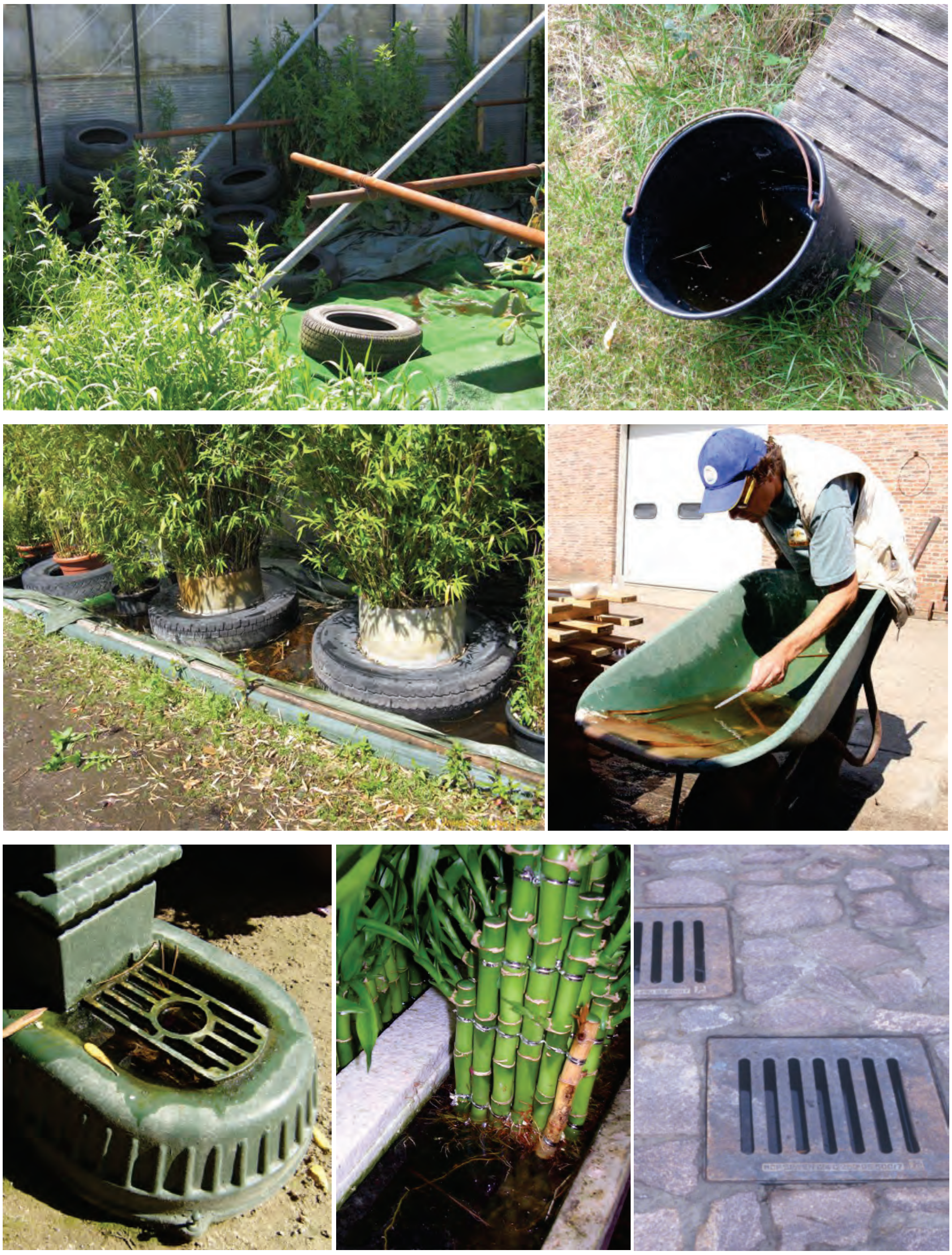

Photos: E.-J. Scholte, Dutch National Centre for Monitoring of Vectors (CMV), NVWA; F. Schaffner (bottom right)

Figure H: Example of placement of traps and definition of the area to be surveyed at an infested storage site for used tyres

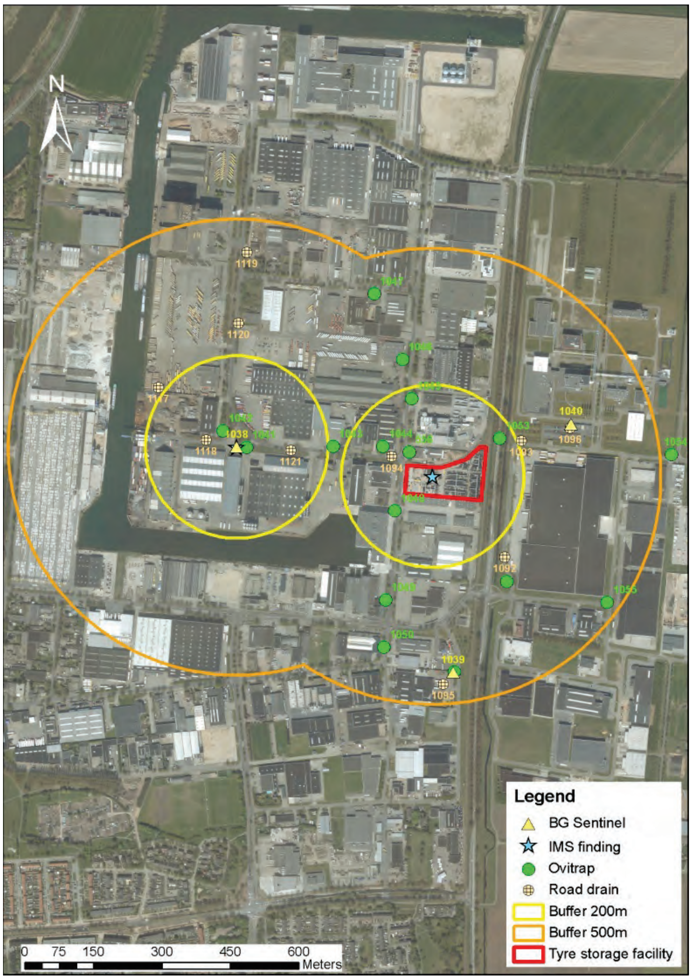

In this particular case, the yellow circle (200-metre buffer zone) corresponds to the area where adulticiding treatment is allowed (following the detection of IMS specimen); the 500-metre buffer zone corresponds to the area where traps are placed and larval control is carried out in all potential larval breeding places.

Source: Dutch National Centre for Monitoring of Vectors (CMV), NVWA

Surveillance of surroundings (cf. Table 4)

Most colonised areas can only be imprecisely delineated. Considering the preference of IMS for urban and suburban areas (including city parks, industrial areas, and residential areas), the surveillance of surrounding areas should focus on sites that offer conditions conducive to mosquitoes. In many European countries, cemeteries offer favourable conditions to mosquitoes, e.g. dense and varied vegetation, road drains, small water containers such as vases and fountains, and hosts (Schaffner et al. 2009). Alternatively, community gardens and parks can be used as sites for sampling. Industrial areas, and in particular company yards with outdoor storage, should also be inspected. Finally, forests can be investigated for water-containing tree holes. By contrast, pastures, cropland, and nature areas such as lakeshores and deltas can be omitted.

In a site offering favourable conditions to mosquitoes, traps should be placed every 50 and then 100 meters in all directions, extending up to 500 meters beyond the site's boundaries (see example in Figure H). Around the focus, a buffer zone of 10 km may be considered for surveying for possible presence and spread of IMS. Traps may be placed at 0.5 to 1 km intervals in all directions. For long-distance spread along roads, it is advisable to place traps at the first stops along the main road axes that originates in the infested focus (parking lots, petrol stations). In addition, the most frequent final destinations should be examined, up to a 2–2½ hours' drive.

The total area to be surveyed should be defined by looking at the local conditions and facilities. Higher altitude areas (hills and mountains) should not be omitted, as, for example, *Ae. japonicus* has been observed to colonise areas at altitudes of up to 1 400 meters (or even above) in central Europe. Finally, surveillance should be strengthened around introduced MBD cases, as well as in city areas with residents that travel frequently to visit relatives in MBD-endemic areas. The surveillance of surrounding areas may benefit from passive surveillance:

informing the general public and/or children at schools, requesting feedback, and sending mosquito specimen to the authorities may help detect new IMS-infested sites.

For checking abundance/density of adult IMS, a minimum number of traps must be used to get a reliable answer. Ovitraps are ideal as they are the most economical choice (i.e. 7 EUR per ovitrap, assuming 26 collection visits; Carrieri et al. 2011). It is possible to calculate the optimum number of ovitraps needed to get reliable results in larger areas. For the major urban areas of Emilia-Romania region in Italy, which suffered a chikungunya outbreak in 2007, the optimal number (for an urban area of 600 ha) varies from 80 to 166 ovitraps (precision level D = 0.2), and from 19 to 96 traps (precision level D = 0.3) (Carrieri et al. 2011; Albieri et al. 2010). In order to assess the efficiency of control measures and estimate mosquito population parameters (e.g. abundance and seasonal dynamics), surveillance measures should cover large areas; it is, however, often sufficient to select two or three representative sites.

Key references

Albieri A, Carrieri M, Angelini P, Baldacchini F, Venturelli C, Mascali Zeo S, et al. Quantitative monitoring of *Aedes albopictus* in Emilia-Romagna, Northern Italy: cluster investigation and geostatistical analysis. Bull Insectol. 2010; 63(2):209-216.

Almeida AP, Gonçalves YM, Novo MT, Sousa CA, Melim M, Gracio AJ. Vector monitoring of *Aedes aegypti* in the Autonomous Region of Madeira, Portugal. Euro Surveill. 2007;12(46):pii=3311. Available from: http://www.eurosurveillance.org/ViewArticle.aspx?ArticleId=3311

Beeuwkes J, den Hartog W, Dik M, Scholte EJ. Surveillance and findings of exotic mosquitoes in used tires in The Netherlands: a methodological approach. Proc Neth Entomol Soc Meet. 2011;22:31-37.

Bellini R, Carrieri M, Burgio G, Bacchi M. Efficacy of different ovitraps and binomial sampling in *Aedes albopictus* surveillance activity. J Am Mosq Control Assoc. 1996;12:632-36.

Bellini R, Veronesi R, Alberani A, Pandolfi N. Improving carbon dioxide trap performances in mosquito monitoring in Italy. 69th AMCA Ann Meet, 2003 March 1-6, Minneapolis, MN. [poster]

Bhalala H, Arias JR. The Zumba mosquito trap and BG-Sentinel trap: novel surveillance tools for host-seeking mosquitoes. J Am Mosq Control Assoc. 2009;25(2):134-9.

Bidlingmayer WL. The measurement of adult mosquito population changes – some considerations. J Am Mosq Control Assoc. 1985;1:328-48.

Bitzhenner M, Guaraglia C, Geier M, Rose A, Talbalaghi A. Evaluation of the BG-Sentinel, a new monitoring trap for mosquitoes in northern Italy. 4th International Congress Society for Vector Ecology. 2005 October 2−7, Reno, NV. [poster]

Bogojević MS, Hengl T, Merdić E. Spatiotemporal monitoring of floodwater mosquito dispersal in Osijek, Croatia. J Am Mosq Control Assoc. 2007;2:99-108.

Çaglar SS, Alten B, Bellini R, Simsek FM, Kaynas S. Comparison of nocturnal activities of mosquitoes (Diptera: Culicidae) sampled by New Jersey light traps and CO_2 traps in Belek, Turkey. J Vector Ecol. 2003; 28(1):12-22.

Canyon DV, Hii JL. Efficacy of carbon dioxide, 1-octen-3-ol, and lactic acid in modified Fay-Prince traps as compared to man-landing catch of *Aedes aegypti*. J Am Mosq Control Assoc. 1997;13:66-70.

Carrieri M, Albieri A, Angelini P, Baldacchini F, Venturelli C, Mascali Zeo S, et al. Surveillance of the chikungunya vector *Aedes albopictus* (Skuse) in Emilia-Romagna (northern Italy): organizational and technical aspects of a large scale monitoring system. J Vector Ecol. 2011;36(1):108-16.

Carrieri M, Angelini P, Venturelli C, Maccagnani B, Bellini R. *Aedes albopictus* (Diptera: Culicidae) population size survey in the 2007 Chikungunya outbreak area in Italy. I. Characterization of breeding sites and evaluation of sampling methodologies. J Med Entomol. 2011;48(6):1214-25.

Carrieri M, Bellini R, Maccaferri S, Gallo L, Maini S, Celli G. Tolerance thresholds for *Aedes albopictus* and *Aedes caspius* in Italian urban areas. J Am Mosq Control Assoc. 2008;24(3):377–86.

Carrieri M., Masetti A., Albieri A., Maccagnani B., Bellini R. Larvicidal activity and influence of *Bacillus thuringiensis* var. *israelensis* on *Aedes albopictus* oviposition in ovitraps during a two-week check interval protocol. J Am Mosq Control Assoc. 2009;25:149-55.

Chan KL. Method and indices used in the surveillance of dengue vectors. Mosquito-Borne Dis Bul. 1985;1:79-88.

European Centre for Disease Prevention and Control (ECDC). Development of *Aedes albopictus* risk maps. ECDC technical report. Stockholm: ECDC; 2009. Available from: http://ecdc.europa.eu/en/publications/Publications/0905_TER_Development_of_Aedes_Albopictus_Risk_Maps.pdf

Farajollahi A, Kesavaraju B, Price DC, Williams GM, Healy SP, Gaugler R, et al. Field Efficacy of BG-sentinel and Industry-Standard Traps for *Aedes albopictus* (Diptera: Culicidae) and West Nile Virus Surveillance. J Med Entomol. 2009;46(4):919-25.

Fay RW, Eliason DA. A preferred oviposition site as a surveillance method for *Aedes aegypti*. Mosq News. 1966;26:531-5.

Focks DA. A review of entomological sampling methods and indicators for dengue vectors. Special Programme for Research and Training in Tropical Diseases, TDR/IDE/Den/03.1. Geneva: World Health Organization; 2003. Available from: http://whqlibdoc.who.int/hq/2003/TDR_IDE_DEN_03.1.pdf

Focks DA, Brenner RJ, Hayes J, Daniels E. Transmission thresholds for dengue in terms of *Aedes aegypti* pupae per person with discussion of their utility in source reduction efforts. Am J Trop Med Hyg. 2000;62(1):11-8.

Gratz NG, Steffen R., Cocksedge W. Why aircraft disinsection? Bull World Health Organ. 2000;78(8):995-1004.

Headlee TJ. The development of mechanical equipment for sampling the mosquito fauna and some results of its use. Proc Ann Meet N J Mosq Exterm Assoc. 1932;19:106-28.

Kröckel U, Rose A, Eiras AE, Geier M. New tools for surveillance of adult yellow fever mosquitoes: comparison of trap catches with human landing rates in an urban environment. J Am Mosq Control Assoc. 2006;22:229-38.

Maciel-de-Freitas R, Eiras ÁE, Lourenço-de-Oliveira R. Field evaluation of effectiveness of the BG-sentinel, a new trap for capturing adult *Aedes aegypti* (Diptera: Culicidae). Mem Inst Oswaldo Cruz. 2006 ;101(3):321-5.

Marques CC, Marques GR, de Brito M, dos Santos Neto LG, Ishibashi VC, Gomes FA. Comparative study of larval and ovitrap efficacy for surveillance of dengue and yellow fever vectors. Rev Saude Publica. 1993;27:237-41.

Meeraus WH, Armistead JS, Arias JR. Field comparison of novel and gold standard traps for collecting *Aedes albopictus* in northern Virginia. J Am Mosq Control Assoc. 2008;24(2):244-8.

Micks DW, Moon WB. *Aedes aegypti* in a Texas coastal county as an index of dengue fever receptivity and control. Am J Trop Med Hyg. 1980;29:1382-8.

Mogi M., Yamamura N. Estimation of the attraction range of a human bait for *Aedes albopictus* (Diptera, Culicidae) adults and its absolute density by a new removal method applicable to populations with immigrants. Res Pop Ecol. 1981;23:328-43.

Morris CD, Clanton KB. Quantification of nuisance mosquito problem in Florida. J Am Mosq Control Assoc. 1988;4:377-9.

Morris CD, Clanton KB. Significant association between mosquito control service requests and mosquito populations. J Am Mosq Control Assoc. 1989;5:36-41.

Morris CD, Clanton KB. Comparison of people who request mosquito control services and their non-requesting neighbours. J Am Mosq Control Assoc. 1992;8:65-8.

Morris CD, Larson VL, Lounibos LP. Measuring mosquito dispersal for control programs. J Am Mosq Control Assoc. 1991;7:608-15.

Nie W-Z, Li J-C, Li D-X, Wang R-J, Gratz N. Mosquitoes found aboard ships arriving at Qinhuangdao Port, P. R. China. Med Entomol Zool. 2004;55:333-5.

Niebylski ML, Craig GB Jr. Dispersal and survival of *Aedes albopictus* at a scrap tire yard in Missouri. J Am Mosq Contr Assoc. 1994;10:339-43.

Nowell WR. Possible introduction of *Aedes albopictus* into Texas from Vietnam. J Am Mosq Control Assoc. 1996;12(4)743-4.

Ordòňez-Gonzales JG, Mercado-Hernandez R, Flores-Suarez AE, Fernández-Salas I. The use of sticky ovitraps to estimate dispersal of *Aedes aegypti* in Northeastern Mexico. J Am Mosq Control Assoc. 2001;17:93-7.

Pluskota B, Storch V, Braunbeck T, Beck M, Becker N. First record of *Stegomyia albopicta* (Skuse) (Diptera: Culicidae) in Germany. Eur Mosq Bull. 2008;26:1-5.

Read NR, Rooker JR, Gathman JP. Public perception of mosquito annoyance measured by a survey and simultaneous mosquito sampling. J Am Mosq Control Assoc. 1994;10:79-87.

Richards SL, Apperson CS, Ghosh S-K, Cheshire HM, Zeichner BC. Spatial analysis of *Aedes albopictus* (Diptera: Culicidae) oviposition in suburban neighborhoods of a Piedmont community in North Carolina. J Med Entomol. 2006;43:976-89.

Romero-Vivas CM, Falconar AK. Investigation of relationships between *Aedes aegypti* egg, larvae, pupae, and adult density indices where their main breeding sites were located indoors. J Am Mosq Control Assoc. 2005;21:15-21.

Schaffner F, Kaufmann C, Hegglin D, Mathis A. The invasive mosquito *Aedes japonicus* in Central Europe. Med Vet Entomol. 2009;23:448-51.

Service MW. Chapter 2 Sampling the larval population: *Aedes aegypti* indices. In: Service MW. Mosquito ecology: field sampling methods. Second edition. London and New York: Elsevier Applied Science; 1993. p157-66.

Service MW. Mosquito (Diptera: Culicidae) dispersal – the long and short of it. J Med Entomol. 1997;34:579-88.

Southwood TRE. Ecological methods with particular reference to the study of insect populations. London: Chapman & Hall; 1978.

Werner D, Kronefeld M, Schaffner F, Kampen H. Two invasive mosquito species, *Aedes albopictus* and *Aedes japonicus japonicus*, trapped in south-west Germany, July to August 2011. Euro Surveill. 2012;17(4):pii=20067. Available from: http://www.eurosurveillance.org/ViewArticle.aspx?ArticleId=20067

Williams CR, Long SA, Russel RC, Ritchie SA. Field efficacy of the BG-sentinel compared with CDC backpack aspirators and CO_2-baited EVS traps for collection of adult *Aedes aegypti* in Cairns, Queensland, Australia. J Am Mosq Control Assoc. 2006;22(2):296-300.

Williams CR, Long SA, Russell RC, Ritchie SA. Optimizing ovitrap use for *Aedes aegypti* in Cairns, Queensland, Australia: effects of some abiotic factors on field efficacy. J Am Mosq Control Assoc. 2006;22:635-40.

Zhang L-Y, Lei C-L. Evaluation of sticky ovitraps for the surveillance of *Aedes (Stegomyia) albopictus* (Skuse) and the screening of oviposition attractants from organic infusions. Ann Tropical Med Parasitol. 2008;102(5):399-407

Annex 4: Identification of invasive mosquito species

1 Mosquito gender determination

Figure A: Mosquito gender identification

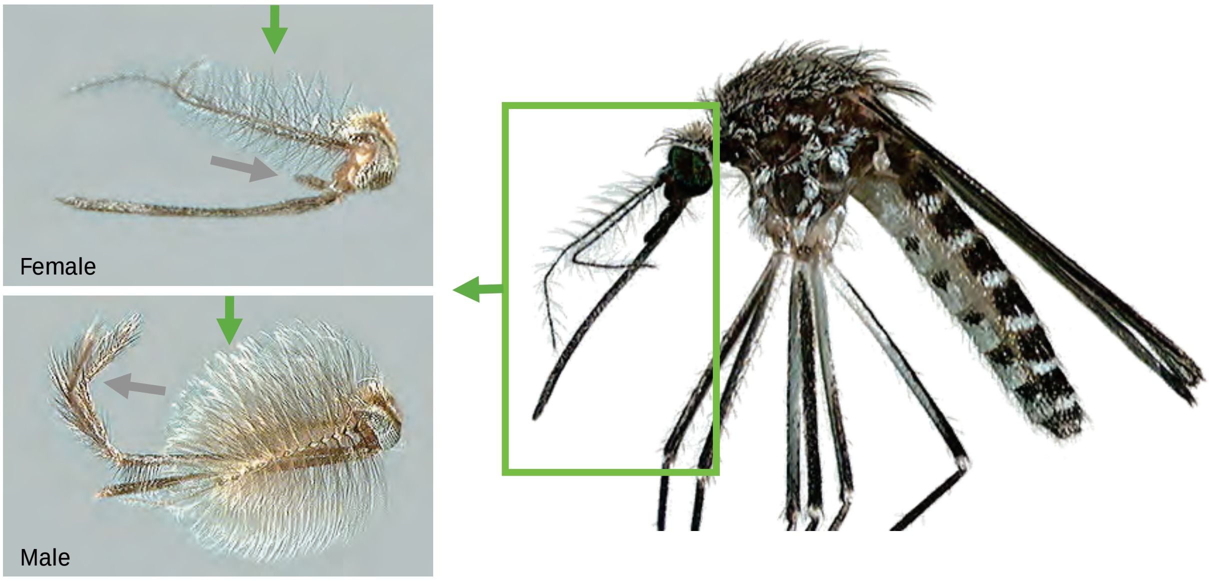

Gender can be identified by a rapid check of the head: Aedes *females have short palps (grey arrow), while males have long palps. Female antennae (green arrow) bear short setae; in males, the setae are long and more numerous. This is valid for all* Culicine *mosquitoes, whereas for* Anopheline *the palps are also long in females.*

2 Methods for rearing mosquito samples for identification

In some cases it is useful to rear the **larvae** (in secured conditions, to avoid any adult escaping) and identify emerging adults, as some IMS-like *Ae. albopictus* and *Ae. cretinus* are easier to differentiate from other species as adults. For rearing, immatures can be transported in plastic vials (optimal volume 0.5–1 l; cf. Figure A in Annex 3), half-filled with water from the breeding site, and kept cool during transport (coolbox). Larval/pupal density must not be excessive (maximum of 100 larvae per 0.25 l). Adults will emerge after a few days if the immatures are stored at around 25 °C, preferably in a 'mosquito breeder' (clear plastic vial with a funnel-shaped lid above the water surface and a full-cover lid, preventing the adult from drowning or escaping). If the larvae are young instars, some food can be provided (finely ground fish food) until they pupate.

Larvae (and adults) can also be reared from **eggs** to allow easier identification. After at least eight days of maturation (at storage conditions described in Chapter 2.2), the eggs must be immersed in tap water (together with the oviposition support). Unfortunately, larvae are often reluctant to hatch from the egg (especially for *Ae. japonicus*), although the hatching rate can be improved if some larval food (aquarium fish food, ground-up rat or dog food) or vitamin C is added to the water at the time of immersion, or by exposing the eggs to a depressurised environment for one hour. This can be repeated several times if needed, with careful desiccation of the eggs in between. Eggs from a single egg batch will not necessarily hatch at the same time, and eggs laid in late autumn may have entered into diapause, in which case a slow process is required to break the diapause.

Figure B: Rearing of IMS in secured laboratory

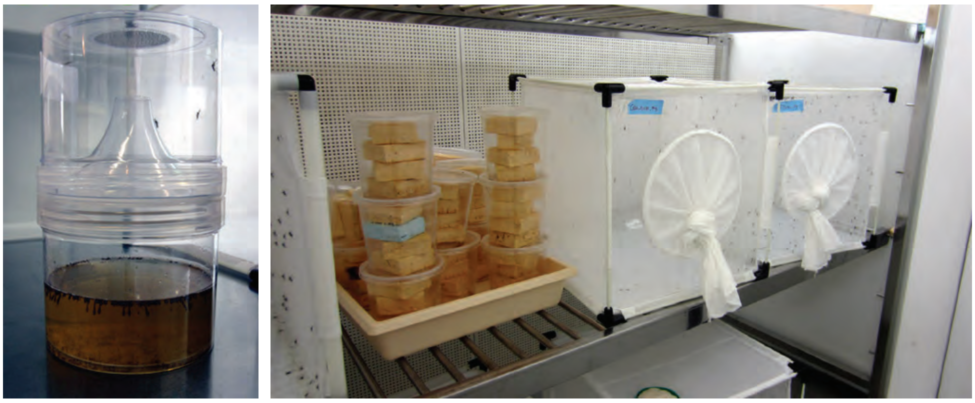

Left: mosquito breeder; right: eggs on polystyrene and adults in cages, in a climate chamber.

Photos: F. Schaffner/IPZ (2012)

3 Available tools for identifying IMS by morphology

Table A: List of available major identification tools with their geographical competence range, stages and invasive species included.

Identification tool	Competence range	Stages	*Ae. aegypti*	*Ae. albopictus*	*Ae. atropalpus*	*Ae. japonicus*	*Ae. koreicus*	*Ae. triseriatus*
A key for the rapid identification of European container-breeding Aedine mosquitoes (larvae and adult females) [see Section 4 below]	• Dichotomic key<br>• Whole Europe<br>• 16 Aedine spp.	L,F	✓	✓	✓	✓	✓	✓
Becker N, Petric D, Zgomba M, Boase C, Madon M, Dahl C, et al. Mosquitoes and their control. 2nd ed. Heidelberg: Springer; 2010.	• Illustrated dichotomic keys<br>• Whole Europe<br>• 94 spp./sub-spp.	L, F, M	✓	✓	-	-	-	-
Darsie RJ, Ward RA. Identification and geographic distribution of mosquitoes of North America, north of Mexico. Mosq Syst. 1981;1:1–313.	• Illustrated dichotomic keys<br>• North America (without Mexico)<br>• 174 spp.	L, F	✓	✓	✓	✓	-	✓
Schaffner F. Mosquitoes in used tyres in Europe: species list and larval key. Eur Mosq Bull. 2003;16:7-12.	• Dichotomic key<br>• Whole Europe<br>• 17 spp.	L	-	✓	✓	✓	-	-
Schaffner F, Angel G, Geoffroy B, Hervy JP, Rhaiem A, Brunhes J. The mosquitoes of Europe. An identification and training programme [CD-ROM]. Montpellier: IRD Éditions & EID Méditerrannée; 2001.	• Illustrated computer-aided identification system<br>• Whole geographical Europe[1]<br>• 100 spp./sub-spp.	L, F, M	✓	✓	✓	✓	-	-
Tanaka K, Mizusawa K, Saugstad ES. A revision of the adult and larval mosquitoes of Japan (including the Ryukyu Archipelago and the Ogasawara islands) and Korea (Diptera: Culicidae). Contrib Am Entomol Inst (Ann Arbor) 1979, 16:1-987.	• Dichotomic keys<br>• Southeast Asia<br>• 113 spp.	L, F, M	✓	✓	-	✓	✓	-
Matsuo K, Yoshida Y, Kunou I. The scanning electron microscopy of mosquitoes (Diptera: Culicidae). I. The egg surfaces of five species of *Aedes* and *Armigeres subalbatus*. J Kyoto pref Univ Med. 1972;81(7):358-63.	• Dichotomic key<br>• Japan<br>• 6 spp.	E	✓	✓	-	✓	-	-
Gutsevich AV, Monchadskii AS, Shtakel'berg, AA. Mosquitoes. Family Culicidae. Fauna of the USSR. Diptera. Volume 3. No. 4. Jerusalem: Isreal Program for Scientific Translations; 1974. [Original publication, in Russian: Leningrad, 1970].	• Dichotomic key<br>• Eastern Europe and north palaearctic region<br>• 110 spp./sub-spp.	L, F, M	✓	✓	-	✓	✓	-

[1] Except some east-European countries
E: eggs; F: female; L: larvae; M: male.

Only tools published in English and valid for large geographical areas were considered.

4 A key for the rapid identification of European container-breeding Aedine mosquitoes (larvae and adult females)

There is no identification tool available which includes all six IMS reported in Europe (cf. Table A). The available identification keys – which also include some IMS – cover a large spectrum of other species; consequently, the identification process can be rather time-consuming, unless it is multi-access (as in a computer-aided system). It is therefore suggested that a specific tool should be developed to rapidly and easily distinguish IMS from other container-breeding mosquitoes; this tool should also be specifically adapted to the surveillance of IMS.

The 37 species and sub-species reported in Europe as breeding in natural or artificial containers are listed in Table B. As a first step, a written dichotomous key is provided here for (1) sorting mosquito genera; and (2) identifying all European container-breeding Aedine species, for both larvae and adult females.

Frequency of reports of container-breeding mosquito species according to their preferences for larval breeding sites, in their respective distribution area	Tyre	Road drains	Other man-made containers	Phytotelms	Soft water rock pool	Salt water rock pool	Natural (non-container)
Ae. aegypti	+++	++	+++	++	++	-	-
Ae. albopictus	+++	++	+++	++	++	-	-
Ae. atropalpus	+++	-	+++	+	+++	-	-
Ae. berlandi	-	-	-	+++	-	-	-
Ae. cretinus	+	-	+	+++	-	-	-
Ae. eatoni	-	-	+	+++	-	-	-
Ae. echinus	-	-	-	+++	-	-	
Ae. geniculatus	++	-	+	+++	-	-	-
Ae. gilcolladoi	-	-	-	+++	-	-	-
Ae. j. japonicus	+++	+++	+++	++	+++	-	-
Ae. koreicus	+++	+	+++	+	++	-	+[2]
Ae. mariae[2]	-	-	-	-	-	+++	-
Ae. p. pulcritarsis	-	-	-	+++	-	-	-
Ae. triseriatus	++	-	+	+++	+	-	-
Ae. vittatus	-	-	-	-	++	-	-
Ae. zammitii[1]	-	-	-	-	-	+++	-
An. claviger	++	-	+	-	+	-	+++
An. maculipennis s.l.	+	-	+	-	-	-	+++
An. petragnani	+	-	+	-	+	-	+++
An. plumbeus	++	+	+	+++	-	-	-
Cs. annulata	++	-	++	-	-	-	+++
Cs. atlantica	-	-	-	-	+++	-	-
Cs. bergrothi	-	-	-	-	+	-	++
Cs. glaphyroptera	+	-	+	-	+	-	++
Cs. longiareolata	++	++	+++	-	++	-	++
Cs. subochrea	-	-	++	-	-	-	+++
Cx. h. hortensis	++	+	+++	-	+++	-	+++
Cx. h. maderensis	-	-	+	-	+++	-	+
Cx. impudicus	-	-	-	-	+	-	+++
Cx. laticinctus	-	-	++	-	++	-	+++
Cx. mimeticus	-	-	-	-	+	-	+++
Cx. perexiguus	-	-	+	-	-	-	+++
Cx. pipiens	+++	+++	+++	+	++	-	+++
Cx. territans	-	-	+	-	-	-	+++
Cx. theileri	-	-	++	-	+	-	+++
Cx. torrentium	+	+	+++	-	-	-	+++
Or. pulcripalpis	-	-	-	+++	-	-	-

[1] Also salt water containers [2] Road tracks, ditches, garden ponds

+++ frequently reported; ++ occasionally reported; + rarely reported; - not reported at all.

Key to larvae (4th instar) of container-breeding Aedine mosquitoes of Europe

1 Genera

1 Siphon absent .... *Anopheles*

Siphon well developed 2

2 Pecten absent .... *Orthopodomyia pulcripalpis*

Pecten present 3

3 Siphonal setae (1-S) consisting of three or more pairs .... *Culex*

Siphonal setae (1-S) consisting of a single pair 4

4 Siphonal setae (1-S) inserted near base of siphon .... *Culiseta*

Siphonal setae (1-S) inserted nearer to middle of siphon .... *Aedes* (incl. *Ochlerotatus*)

2 Aedes (incl. Ochlerotatus)

1 Siphonal index < 4.0; pecten teeth long and spine-like 2

Siphonal index > 4.0; pecten teeth short and scale-like 14

2 Cephalic seta 5-C multiple (4–7) branched and inserted far forwards on the front at level of 4- and 6-C and anterior to 7-C 3

Cephalic seta 5-C simple or double branched and inserted far posterior to 4- and 5-C and at level of or posterior to 7-C 5

3 Pecten spines all evenly spaced 4

Pecten with one or more distal spines more widely spaced .... *Ae. japonicus*

4 Siphonal index > 2.5; saddle bearing developed spicules at its distal margin .... *Ae. koreicus*

Siphonal index < 2.5; saddle bearing long spines at it distal margin [Madeira and Canary archipelagos] ... *Ae. eatoni*

5 Pecten with one or two distal spines more widely spaced 6

Pecten spines all evenly spaced 7

6 Comb of abdominal segment VIII developed in a triangular patch of more than 20 fringed scales .... *Ae. atropalpus*

Comb of abdominal segment VIII developed in a single row of 6–9 large and pointed scales .... *Ae. vittatus*

7 Antennal seta 1-A simple 8

Antennal seta 1-A with 6–9 branches 15

8 Abdominal setae 1 of segment I not longer than half of the length of the segment, with at most four branches and slightly stellate 9

Abdominal setae 1 of segment I as long or longer as the segment, with at least four branches and clearly stellate 12

9 Thoracic setae 10-12-M and 10-12-T inserted near a small tooth; comb scales with a strong central tooth and several minute basal denticles or without denticles 10

Thoracic setae 10-12-M and 10-12-T inserted near a stout spine; comb scales with well developed basal denticles each side of central tooth .... *Ae. aegypti*

10 Comb of segment VIII in a single regular row of scales with a strong central tooth and several minute basal denticles; saddle lateral seta 1-X simple or double branched 11

Comb of segment VIII in a single or partly double irregular row of scales with a strong central tooth and without denticles; saddle lateral seta 1-X multiple (5–7) branched .... *Ae. triseriatus*

11 Outer cephalic seta 7-C with 2–3 branches; additional seta on the siphon .... *Ae. albopictus*

Outer cephalic seta 7-C simple; in addition to the siphonal tuft 1-S, a simple seta is inserted laterally within the apical third of the siphon .... *Ae. cretinus*

12 Branches of stellate seta 1 on abdominal segment I about the same length as the segment; pecten 1/4–2/5 as long as the siphon 13

Branches of stellate seta 1 on abdominal segment I longer than the length of the segment; pecten at least half as long as the siphon .... *Ae. echinus*

13 Comb scale number usually less than 16; cephalic setae 6-C with 1–2 branches .... *Ae. geniculatus*

Comb scale number usually more than 17; cephalic setae 6-C with 3 or more branches........ *Ae. gilcolladoi*

14 Siphon extremely long and slender, siphonal index > 5.5; comb of segment VIII developed in a triangular patch of 13–24 scales .... *Ae. berlandi*

Siphon shorter, siphonal index 4.0–5.0; comb of segment VIII developed in a more or less irregular row of 6–10 scales .... *Ae. pulcritarsis*

15 Antenna with numerous spicules; pecten teeth with less than 4 lateral denticles at the base; comb of segment VIII with usually less than 16 scales .... *Ae. zammitii*

Antenna with less numerous spicules; pecten teeth with 4 or more lateral denticles at the base; comb of segment VIII with usually more than 16 scales .... *Ae. mariae*

Key to adult females of container-breeding Aedine mosquitoes of Europe

1 Genera

1 Maxillary palps as long as proboscis; scutellum evenly rounded and uniformly setose .... *Anopheles*

Maxillary palps distinctly shorter than proboscis; scutellum trilobed, setae arranged in 3 sets........ 2

2 Prespiracular setae present .... *Culiseta*

Prespiracular setae absent 3

3 Tarsomere I of fore legs longer than tarsomeres II to V together; tarsomere IV of fore legs reduced, not longer than broad .... *Orthopodomyia pulcripalpis*

Tarsomere I of fore legs usually shorter than tarsomeres II to V together; tarsomere IV of fore legs not reduced, distinctly longer than broad 4

4 Postspiracular setae present; abdomen tapering apically, cerci long easily visible ... *Aedes* (incl. *Ochlerotatus*)

Postspiracular setae absent; abdomen rounded apically, cerci short, hardly visible .... *Culex*

2 Aedes (incl. Ochlerotatus)

1 Tarsomeres I–III of hind leg with pale rings 2

Tarsomeres I–III of hind leg without pale rings........ 8

2 Pale rings present only at base of tarsomeres........ 3

Each pale ring embraces two tarsomeres, the apex of one and the base of the following tarsomere 11

3 Scutum with white longitudinal stripes; palps with an apical white scale patch........ 4

Scutum with longitudinal yellowish stripes or bands; palps entirely dark or with a few white scales 7

4 Scutum with one or more longitudinal white stripes; tibia of hind leg without a median white ring........ 5

Scutum with two or three pairs of small white spots, distributed along the dorsocentral area; tibia of hind leg with a median white rings........ *Ae. vittatus*

5 Scutum with two narrow medio-dorsal white stripes and 2 broad lateral white stripes, lyre shaped *Ae. aegypti*

Scutum with one acrostrichal white stripe; if lateral stripes are present, they are narrow and do not continue over transverse suture, never lyre-shaped........ 6

6 Acrostrichal white stripe broad; two posterior medio-dorsal white stripes narrow, short, not reaching the middle of scutum; metameron bare .... *Ae. albopictus*

Acrostrichal white stripe narrow; two posterior medio-dorsal white stripes narrow, long, reaching the middle of scutum; metameron with a patch of white scales .... *Ae. cretinus*

7 Hind leg tarsomere IV entirely dark or with a few pale scales at the base (no ring); subspiracular area usually without scale patch .... *Ae. japonicus*

Hind leg tarsomere IV with a short basal pale ring; subspiracular area usually with a patch of pale scales...... .... *Ae. koreicus*

8 Scutum with one medio-dorsal acrostrichal narrow stripe, and with two thin or broad lateral stripes........... 9

Scutum with a small anterior acrostrichal patch of white scales, not prolonged in a medio-dorsal stripe, and with two broad white lateral stripes .... *Ae. triseriatus*

9 Proboscis entirely dark scaled; lateral stripes of scutum broad...10

Proboscis with pale scales at the apex; lateral stripes of scutum narrow [Madeira and Canary archipelagos] .. *Ae. eatoni*

10 Metameron bare .... *Ae. geniculatus, Ae. gilcolladoi*

Metameron with a patch of pale scales.. *Ae. echinus*

11 Tarsomeres V of all legs entirely pale scaled 12

Tarsomeres V of fore and mid legs dark scaled, of hind leg entirely pale scaled 13

12 Hind femur entirely dark; hind tibia only white at the apex; metameron with a patch of pale scales......... *Ae. berlandi*

Hind femur and hind tibia scattered with pale scales; metameron bare .... *Ae. pulcritarsis*

13 Wing veins uniformly dark scaled or with at most a few isolated pale scales; palps entirely dark scaled ... *Ae. atropalpus*

Wing veins covered by mixed dark and pale scales; palps with dark and pale scales................................14

14 Palps with mixed dark and pale scales; metameron with a patch of pale scales........................... *Ae. mariae*

Palps with dark scales at the basis and pale scales at the apex; metameron bare....................... *Ae. zammitii*

5 Identification of eggs of invasive and indigenous European *Aedes* container-breeding species

Table C: References for egg description of invasive and indigenous container-breeding European *Aedes* species

Mosquito species	Egg description
Ae. aegypti	Matsuo et al. 1972; Linley 1989a
Ae. albopictus	Matsuo et al. 1972; Linley 1989a
Ae. atropalpus	Linley & Craig 1994
Ae. berlandi	Encinas-Grandes 1982
Ae. cretinus	-
Ae. eatoni	-
Ae. echinus	-
Ae. geniculatus	Encinas-Grandes 1982
Ae. gillcolladoi	-
Ae. japonicus	Matsuo et al. 1972; Haddow et al. 2009
Ae. koreicus	-
Ae. mariae	-
Ae. pulcritarsis	-
Ae. triseriatus	Zaim et al. 1977, Linley 1989b
Ae. vittatus	Hinton & Service 1969; Encinas-Grandes 1982
Ae. zammitii	-

6 Identification of mosquitoes by molecular tools

When morphological identification is not possible (e.g. when specimens are damaged), mosquitoes can be identified with genetic methods or protein profiling at any biological stage (from egg to adult), using only part of the insect (e.g. a single leg, a thorax).

Molecular, as morphological identification, can be provided as a service by properly equipped laboratories. The process may vary depending on the country, but mainly depends on the number of specimens to be analysed and the level of performance requested (i.e. whole process or only part of it). Indicative estimations can be given from cheapest to the most expensive process and per specimen, as follow: EUR 24 for MALDI-TOF MS, EUR 28 for morphological identification, 32 EUR for PCR, 56 EUR for PCR/sequencing.

Genetic identification

Mosquitoes can be identified with genetic methods at any biological stage (from egg to adults), using only part of the insect (e.g. a single leg, a thorax).

Genetic information of several loci is available for mosquitoes, particularly at the mtDNA CO1 barcode locus (Table D). Polymerase chain reaction (PCR) combined with sequencing of the amplicon can identify a specimen, providing that corresponding sequence data are deposited in the GenBank (http://www.ncbi.nlm.nih.gov/genbank/) data base. For mitochondrial genes, a sequence identity higher than 98% is needed to confirm identification.

For routine identification of mosquitoes, specific conventional real-time PCR assays have been developed in single or multiplexed formats. Unfortunately, no such assays are as yet available for the invasive and indigenous *Aedes* container-breeding species of concern in Europe, but such developments are in progress.

For DNA-based identification, specimens can be used fresh, kept dry (in tubes with silica gel), in 70% or absolute ethanol, or frozen at -20 °C.

Table D: Molecular identification tools applied on invasive and indigenous *Aedes* container-breeding species

Mosquito spp.	Described gene sequences*						PCR assays	MALDI-TOF MS profile
	rRNA ITS1	rRNA ITS2	rDNA 18s	mtDNA CO1	mtDNA CO2	mtDNA ND4		
Ae. aegypti	✓	✓	✓	✓	✓	✓	✓	
Ae. albopictus	✓	✓	✓	✓	✓		✓	
Ae. atropalpus				✓	✓			
Ae. berlandi								
Ae. cretinus		✓						
Ae. eatoni								
Ae. echinus								
Ae. geniculatus				✓				
Ae. gillcolladoi								
Ae. japonicus				✓	✓			✓
Ae. koreicus					✓	✓		
Ae. mariae				✓				
Ae. pulcritarsis								
Ae. triseriatus	✓	✓		✓				
Ae. vittatus			✓				✓	
Ae. zammitii								

** Only the most frequently used sequences are listed; rRNA/DNA: ribosomal RNA/DNA; mtDNA: mitochondrial DNA*

Protein profiling

Identification of mosquitoes by isoenzyme analyses has been described (Rioux et al 1998; Awono et al 2006), but this approach is time-consuming and expensive and has only been used for invasive species in one study; this study revealed diagnostic enzymes that exhibited species-specific patterns that correctly identify *Ae. cretinus* and *Ae. albopictus* (Taafe Gount et al. 2004). For such identifications, specimens can only be used fresh or stored frozen at -80 °C.

As an alternative, protein profiling by using matrix-assisted laser desorption/ionisation time-of-flight mass spectrometry (MALDI-TOF MS) was recently described for the characterisation of *Ae. japonicus*, and this approach was extended to include other mosquito species (Kaufmann et al. 2011) (Table D). MALDI-TOF MS, which is a

rapid, simple, reliable and cost-effective method, was demonstrated to be suitable for the routine species identification of *Culicoides* biting midges (Kaufmann et al. 2012). Reference spectra need to be determined separately for the different developmental stages, and removal of the abdomen is needed to avoid the interference effects of gut contents. The specimens can be analysed fresh or after storage in 70% ethanol for several months.

Key references

Awono-Ambene HP, Simard F, Antonio-Nkondjio C, Cohuet A, Kengne P, Fontenille D. Multilocus enzyme electrophoresis supports speciation within the *Anopheles nili* group of malaria vectors in Cameroon. J Trop Med Hyg. 2006;75(4):656–58.

Bargues MD, Morchón R, Latorre JM, Cancrini G, Mas-Coma S, Simón F. Ribosomal DNA second internal transcribed spacer sequence studies of Culicid vectors from an endemic area of *Dirofilaria immitis* in Spain. Parasitol Res. 2006;99:205-13.

Becker N, Petrić D, Zgomba M, Boase C, Dahl C, Lane J, et al. Mosquitoes and their control. Heidelberg, Dordrecht, New York: Kluwer Academic/Plenum Publishers; 2003.

Becker N, Petrić D, Zgomba M, Boase C, Madon M, Dahl C, et al. Mosquitoes and their control. Heidelberg, Dordrecht, New York: Springer; 2010.

Cameron EC, Wilkerson RC, Mogi M, Miyagi I, Toma T, Kim HC, et al. Molecular phylogenetics of *Aedes japonicus*, a disease vector that recently invaded Western Europe, North America, and the Hawaiian islands. J Med Entomol. 2010 Jul;47(4):527-35.

Cook S, Diallo M, Sall AA, Cooper A, Holmes EC. Mitochondrial markers for molecular identification of *Aedes* mosquitoes (Diptera: Culicidae) involved in transmission of arboviral disease in West Africa. J Med Entomol. 2005;42(1):19-28.

Cywinska A, Hunter FF, Hebert PD. Identifying Canadian mosquito species through DNA barcodes. Med Vet Entomol. 2006 Dec;20(4):413-24.

Darsie RE, Samanidou-Voyadjoglou A. Keys for the identification of the mosquitoes of Greece. J Am Mosq Control Assoc. 1997;13(3):247-54.

Darsie RF, Ward RA. Identification and geographical distribution of the mosquitoes of North America, north of Mexico. University of Florida Press, Gainesville: 2005

Das B, Swain S, Patra A, Das M, Tripathy HK, Mohapatra N, Kar SK, Hazra RK. Development and evaluation of a single-step multiplex PCR to differentiate the aquatic stages of morphologically similar *Aedes* (subgenus: *Stegomyia*) species. Trop Med International Health. 2012;17(2):235-43.

Encinas Grandes A. Taxonomía y biología de los mosquitos del área salmantina (Diptera, Culicidae). Salamanca, Spain Univ. de Salamanca: 1982.

Fonseca DM, Widdel AK, Hutchinson M, Spichiger SE, Kramer LD. Fine-scale spatial and temporal population genetics of *Aedes japonicus*, a new US mosquito, reveal multiple introductions. Molecular Ecology. 2010 Apr;19(8):1559-72.

Gaunt CM, Mutebi JP, Munstermann LE. Biochemical taxonomy and enzyme electrophoretic profiles during development, for three morphologically similar *Aedes* species (Diptera: Culicidae) of the subgenus *Stegomyia*. J Med Entomol. 2004;41(1):23-32.

Gutsevich AV, Monchadskii, Shtakel'berg AA. Fauna of the U.S.S.R. – Diptera: Mosquitoes. Jerusalem: 1974

Haddow AD, Moulton JK, Gerhardt RR, McCuiston LJ, Jones CJ. Description of the egg of *Ochlerotatus japonicus japonicus* (Diptera: Culicidae) using variable pressure scanning electron microscopy. J Med Entomol. 2009;46(1):9-14.

Higa Y, Toma T, Tsuda Y, Miyagi I. A multiplex PCR-based molecular identification of five morphologically related, medically important subgenus *Stegomyia* mosquitoes from the genus *Aedes* (Diptera: Culicidae) found in the Ryukyu Archipelago, Japan. Jpn J Infect Dis. 2010 Sep;63(5):312-6.

Hill LA, Davis JB, Hapgood G, Whelan PI, Smith GA, Ritchie SA, et al. Rapid identification of *Aedes albopictus*, *Aedes scutellaris*, and *Aedes aegypti* life stages using real-time polymerase chain reaction assays. Am J Trop Med Hyg. 2008;79(6):866-75.

Hinton HE, Service MW. The surface structure of aedine eggs as seen with the scanning electron microscope. Ann trop Med Parasitol. 1969; 63(4):409-11.

Ho CM, Liu YM, Wei YH, Hu ST. Gene for cytochrome c oxidase subunit II in the mitochondrial DNA of *Culex quinquefasciatus* and *Aedes aegypti* (Diptera: Culicidae). J Med Entomol. 1995 Mar;32(2):174-80.

Kamgang B, Brengues C, Fontenille D, Njiokou F, Simard F, et al. Genetic structure of the Tiger mosquito, *Aedes albopictus*, in Cameroon (Central Africa). PLoS ONE. 2011;6(5):e20257.

Kaufmann C, Schaffner F, Ziegler D, Pflüger V, Mathis A. Identification of field-caught Culicoides biting midges using matrix-assisted laser desorption/ionization time of flight mass spectrometry. Parasitology, 2012 Feb;139(2):248-58.

Kaufmann C, Ziegler D, Schaffner F, Carpenter S, Pflüger V, Mathis A. Evaluation of matrix-assisted laser desorption/ionization time of flight mass spectrometry for characterization of Culicoides nubeculosus biting midges. Medical and Veterinary Entomology, 2011, 25(1): 32-38

Latrofa MS, Dantas-Torres F, Annoscia G, Genchi M, Traversa D, Otranto D. A duplex real-time polymerase chain reaction assay for the detection of and differentiation between *Dirofilaria immitis* and *Dirofilaria repens* in dogs and mosquitoes. Vet Parasitol. 2012 Apr 30;185(2-4):181-5.

Linley JR, Craig JR. Morphology of long- and short-day eggs of *Aedes atropalpus* and *A. epactius* (Diptera: Culicidae). J Med Entomol. 1994;31(6):855-67.

Linley JR. Comparative fine structure of the eggs of *Aedes albopictus*, *Ae. aegypti*, and *Ae. bahamensis* (Diptera: Culicidae). J Med Entomol. 1989a;26(6):510-21.

Linley JR. Scanning electron microscopy of the egg of *Aedes (Protomacleaya) triseriatus* (Diptera: Culicidae). J Med Entomol. 1989b;26(5):474-8.

Matsuo K, Yoshida Y, Kunou I. The scanning electron microscopy of mosquitoes (Diptera: Culicidae). I. The egg surfaces of five species of *Aedes* and *Armigeres subalbatus*. J Kyoto pref Univ Med. 1972;81(7):358-63.

Medlock J, Hansford KM, Schaffner F, Versteirt V, Hendrickx G, Zeller H, et al. A review of the invasive mosquitoes in Europe: Ecology, public health risks, and control options. Vector Borne Zoonotic Dis. 2012 Mar 26;(12):xxx-xx

Morlais I, Severson DW. Complete mitochondrial DNA sequence and amino acid analysis of the cytochrome C oxidase subunit I (COI) from *Aedes aegypti*. DNA Seq. 2002 Apr;13(2):123-7.

Mousson L, Dauga C, Garrigues T, Schaffner F, Vazeille M, Failloux A-B. Phylogeography of *Aedes (Stegomyia) aegypti* (L.) and *Aedes (Stegomyia) albopictus* (Skuse) (Diptera: Culicidae) based on mitochondrial DNA variations. Genet Res. 2005;86:1-11.

Patsoula E, Samanidou-Voyadjoglou A, Spanakos G, Kremastinou J, Nasioulas G, Vakalis NC. Molecular and morphological characterization of *Aedes albopictus* in northwestern Greece and differentiation from *Aedes cretinus* and *Aedes aegypti*. J Med Entomol. 2006;43(1):40-54.

Reno HE, Vodkin MH, Novak RJ. Differentiation of *Aedes triseriatus* (Say) from *Aedes hendersoni* Cockerell (Diptera: Culicidae) by restriction fragment length polymorphisms of amplified ribosomal DNA. Am J Trop Med Hyg. 2000 Feb;62(2):193-9.

Rey D, Despres L, Schaffner F, Meyran JC. Mapping of resistance to vegetable polyphenols among *Aedes* taxa (Diptera, Culicidae) on a molecular phylogeny. Mol Phylog Evol. 2001;19(2):317-25.

Ribeiro H, Cunha RH. Identification keys of the mosquitoes (Diptera: Culicidae) of Continental Portugal, A cores and Madeira. Eur Mosq Bull. 1999;3:1-11.

Rioux JA, Guilvard E, Pasteur N. Description d'*Aedes (Ochlerotatus) coluzzii* n. sp. (Diptera, Culicidae), espèce jumelle A du complexe detritus. Parassitologia. 1998;40:353-60.

Schaffner F, Angel G, Geoffroy B, Hervy J-P, Rhaiem A, Brunhes J. The Mosquitoes of Europe/Les moustiques d'Europe. An identification and training programm [CD ROM software]/Logiciel d'identification et d'enseignement. Montpellier: IRD Editions & EID Méditerranée; 2001.

Schaffner F. Mosquitoes in used tyres in Europe: species list and larval key. Eur Mosq Bull. 2003;16:7-12.

Tanaka K, Mizusawa K, Saugstad ES. A revision of the adult and larval mosquitoes of Japan (including the Ryukyu Archipelago and the Ogasawara Islands) and Korea (Diptera: Culicidae). Contributions of the American Entomological Institute (Ann Arbor). 1979;16(1):1-987.

Wang J, Huang C. Molecular divergence of the mitochondrial cytochrome oxidase II gene in three mosquitoes. J Am Mosq Control Assoc. 2002;18(4):301-6.

Widdel AK, McCuiston LJ, Crans WJ, Kramer LD, Fonseca DM. Finding needles in the haystack: single copy microsatellite loci for *Aedes japonicus* (Diptera: Culicidae). Am J Trop Med Hyg. 2005 Oct;73(4):744-8.

Zaim M, Lewandowski HB, Newson HD, Hooper GR. Differentiation of *Aedes triseriatus* and *Ae. hendersoni* (Diptera: Culicidae) based on the surface structure of the egg shell. J Med Entomol. 1977;14(4):489-90.

Annex 5: Principles and detailed methodologies for the determination of mosquito population parameters

Table A: Specific methods, tool, indices and formulas for determination of mosquito population key parameters and procedures

Parameters and procedures	Methods	Tools	Indices	Formulas
**Population abundance**	Breeding site surveys: inspection of juvenile habitats	Dipper, water net	Container index, vase index	Percent of positive containers/vases
			House index	Percent of houses with at least one positive breeding site
			Breteau index	Number of positive breeding sites per 100 premises
	Ovitrap surveys	Ovitraps	Ovitrap index	Average proportion of ovitraps with mosquito eggs
			Trap positivity index	Proportion of positive traps
			Egg density index	Ratio total no. egg/total no. traps
	Pupal demographic surveys	Dipper, water net	Pupae per premise index	Number of pupae per premise
			Pupae per hectare index	Number of pupae per hectare
	Adult trapping with adequate trap for IMS	Adult trap	Adult mosquito per trap/period	No. of females per trap/period
		Resting box/trap, aspirator, etc.	Human blood index	Mosquito no. fed on human/total no.
	Anthropophilic adult catching	HLC, mouth aspirator	Human landing rate	No. of females/man/15 minutes
			Nuisance threshold	
**Female longevity, gonotrophic cycle and dispersal**	Mark-release-recapture	Rearing facilities, marking equipment, adult traps or aspirators	Mean distance travelled	Mean distance in km
			Maximum distance travelled	Maximum distance in km
			Flight range	The maximum distance reached by a certain part of the population (e.g. 90% of the marked adults)
	Laboratory experiments	Rearing facilities, flight mills	Flight performance	Mean/maximum distance in km
	Laboratory experiments	Rearing facilities under specific climatic conditions	Adult longevity; gonotrophic cycle	Weibull model;<br>Time period between two consecutive blood meals
	Field experiments	Host-seeking adult traps; mark-release-recapture	Parity rate; daily survival rate	No. parous females/total no.; daily probability of survival
**Female biting and resting behaviour**	Daily biting activity	Baited trap or HLC	Circadian activity	Biting activity per 15 minutes (HLC) or per hour (trap) over 24 hours
	Blood fed females trapping/collection	Blood meal analysis, traps, aspirators	Host feeding indices, anthropophily	Mosquito no. fed on host or human /total no.
	Endophagy/exophagy comparison	Baited trap or HLC	Endophagy rate	Percent of females biting indoor
	Endophily/exophily comparison	Resting catches/traps	Endophily rate	Percent of females resting indoor after the blood meal
	Indoor experiments for host preferences and repellency	Olfactometers or cages, hosts	Host feeding patterns	Percent of females feeding on different hosts or treated/untreated skin
	Outdoor experiments for host preferences	Host-baited traps, odour-baited traps	Host feeding patterns	Percent of females per different host
**Population vector competence**	Vector susceptibility/ competence studies	Rearing and infection facilities in BL3	Infection rate	Percent of females with infected head
			Dissemination rate	Percent of females with infected saliva
**MBD risk assessment**	Gathering field and laboratory data on abundance, longevity, biting behaviour and dispersal	Mathematical/ statistical analysis, modelling, mapping	Gonotrophic cycle	The time females spend a) from finding a host to laying the eggs in nature or b) from a blood meal to laying eggs in a laboratory.

Parameters and procedures	Methods	Tools	Indices	Formulas
			Vector capacity (mosquito component of the basic reproduction number R_0)	The average number of inoculations from a single case of disease in a day, from vector population to man, assuming that all mosquitoes that bite an infected person get infected.
			Basic reproduction number R_0 [The disease can invade/maintains if R_0>1, whereas it cannot/decreases if R_0<1.]	The average number of secondary cases of disease arising from each primary infection in a certain population of susceptible humans/hosts.

Information about IMS mosquito population parameter values from specific national/regional contexts can be found in literature or obtained through exchanges with research teams and experts.

1 Principles

Mosquito population parameters and vector capacity

The life cycle of female mosquitoes requires that physiological needs like sugar, mates, blood, resting places, and oviposition sites are met and satisfied. The results of all activities related to these physiological needs determine the values of a set of parameters which determine a species' vector capacity under local conditions, i.e. population estimates like longevity, dispersal, density, and pathogen transmission. Thus, the comparison of life-history strategies of invasive container-breeding mosquitoes may yield insights into the factors which permit certain species to expand their geographical range. In places where the species is a potential vector of a disease pathogen, these parameters are also important in determining the epidemiological status of the population.

Population estimates for mosquitoes usually describe developmental and life-history parameters such as longevity (including duration of growth of different mosquito life stages – egg, larva, pupa, adult – and their survival rates); the number of gonotrophic cycles per lifetime and their length; fertility; fecundity; mortality, the intrinsic rate of increase; net reproductive (replacement) rate; birth rate; death rate; generation time; host preference; capacity for dispersal; and size of population. Differences in body size of adult mosquitoes can, for example, be important to estimate the size of a population from an epidemiological standpoint. Large *Ae. albopictus* females have higher human host attack rates and obtain multiple blood meals (from multiple hosts) more frequently than small females (Xue et al. 2010), thus spreading the disease more efficiently. Body size may also affect mosquito survival and longevity under natural conditions.

The ability to determine the age structure and the survival rate of female mosquitoes is of paramount ecological importance because longevity affects net reproduction rates and dispersal distance (Service 1999). Knowledge of feeding behaviour (Does a mosquito prefer to take the blood meal from human or from animal host? Does it imbibe on different/multiple hosts before being completely fed? When does it search for a blood meal?) and other aspects of pest insect species life history is of crucial importance to estimating its vector capacity (potential for transmitting the disease) and for assessing the risk of MBD transmission (Is there a mosquito population that can transmit a certain disease present in the country? How numerous is this population? How efficiently can it transmit the disease within human population? What are the pathways for disease/vector introduction from abroad?). Knowledge of population parameters also supports the development of effective control programmes, the evaluation of its impact (Celedonio Hurtado et al. 1988), and the establishment of efficient mass rearing facilities for the sterile insect technique (SIT; successfully used for eradication of some agricultural and veterinary pests from isolated areas). With regard to invasive mosquitoes, population parameters could also help to detect IMS early (and before they could spread from the site of introduction) (Bellini et al. 2002). Other areas include the evaluation of the impact of sterile insect release (Bellini et al. 2007, Bellini et al. 2010), the interpretation of trap data, and the modelling of potential outbreaks.

Mosquito population parameters are monitored for a variety of purposes and by using different approaches. The most important issue in planning the sampling of information is to clearly define the objectives of a study at the beginning of the planning process because this will largely determine the methods to be used. It is also important to consider the type of statistic analysis that will be applied. This will ensure that data are collected, managed and stored (see Chapter 2.6) in the most appropriate manner.

Evidence is accumulating that changes in these parameters may occur in fluctuating mosquito populations, and the population parameters of different cohorts of a species may be quite different (Watson 1967; Honório et al. 2003; Nur Aida et al. 2008; Delatte et al. 2009; Bellini et al. 2010; Marini et al. 2010). In some cases, fluctuation of the vector population parameters might be linked to the adaptation of a population to abiotic factors (see Chapter 7). These parameters need to be determined under controlled conditions to better understand the dynamics of vector populations (e.g. vector capacity), especially in countries with a wide range of temperatures (showing different

climates and boasting a broad altitude range). Therefore, parameters such as fertility, longevity, dispersal and vector capacity should be continually monitored both in the laboratory (e.g. simulating the influence of different temperatures) and in the field for local populations.

Longevity

In order to estimate the longevity of a mosquito population, one needs to collect absolute data or convert relative values. Consequently, this type of research can be done in the laboratory, by rearing the target species (Watson 1967; Tsuda et al. 1991; Fernandez & Forattini 2003; Kosova 2003; Löwenberg Neto & Navarro-Silva 2004; Delatte et al. 2009; Xue et al. 2010), or in the field (Chadee et al. 2009), typically deriving the data from mark-release-recapture (MRR) trials (Conway et al. 1974; Trpis & Hauserman 1986; Lee 1994; Niebylski & Craig 1994; Honório et al. 2003; Russell et al. 2005; Lacroix et al. 2009; Bellini et al. 2010; Marini et al. 2010; Gu et al. 2011). Some authors combine the advantages of controlled and natural environments and set up their experiment in semi-natural/uncontrolled conditions (Bellini et al. 2007; Nur Aida et al. 2008).

Within a given population of mosquitoes, for example, both vector capacity and the extent to which the potential fecundity is realised is influenced by the longevity of the females. The influence of temperature and other environmental factors that are studied in the laboratory can be incorporated into the regression equation. Observations by Richards and Waloff (1954, in: Southwood 2000) show that it may be justified to apply data from the laboratory to populations in the field. Such equations cannot, however, be applied outside the populations from which they were derived.

Blood feeding/host preference

Investigations of mosquito blood feeding and resting behaviour are of crucial importance for areas where epidemics occur and usually comprise the investigation of host-seeking and feeding behaviours on several vertebrate species, the measuring of endophagous/exophagous biting behaviour, endophilic/exophilic resting behaviour, and the mosquito's daily biting activity (recorded over 24 hours). Delatte et al. (2010) showed that *Ae. albopictus* prefer to feed (89% exophagic) and rest (87% exophilic) outdoors in contrast to *Ae. aegypti,* which is well-adapted to the highly urban environments of tropical cities and frequently bite and rest indoors (Lambrechts et al. 2010).

Mosquitoes can be opportunist, feeding on a wide range of cold- and warm-blooded hosts, and thus can be involved in various vertebrate-virus transmission cycles, acting as a bridge vector for zoonotic viruses. Anthropophily (preference for humans) combined with multiple blood feeds during completion of one meal, increases the risks of spreading an arbovirus to the human population.

Blood-feeding behaviour can influence vector potential, depending on the vertebrate host groups with which the mosquito makes contact. If reservoir and amplification hosts (where the pathogen is surviving) are the primary focus of vector blood-feeding, the likelihood of pathogen acquisition by the vector increases. A mosquito species that can feed on wide range of hosts is a potentially dangerous bridge vector of zoonotic pathogens to humans (e.g. West Nile), but in contrast is likely to be less efficient as an epidemic vector of pathogens restricted to humans (e.g. dengue, chikungunya) (Lambrechts et al. 2010).

In addition, the blood-feeding behaviour of a vector may influence the spatial distribution of a disease. The spatial distribution of a population of blood-sucking insects amongst its available vertebrate hosts has important epidemiological consequences for the transmission of vector-borne diseases. In fact, after daily survival rate, vector capacity is the one parameter most sensitive to changes in host preference (Dye & Hasibeder 1986, in: Alonso et al. 2003). Therefore, knowledge of the biological parameters that lead to host choice can be highly relevant for the planning of vector and disease control (McCall & Kelly 2002, in: Alonso et al. 2003).

Population size, mosquito density

When sufficient data are obtained, both relative estimates and population indices can sometimes be related to absolute population (if measured at the same time) by regression analysis. The indices traditionally used to evaluate *Stegomyia* population (e.g. *Ae. aegypti*, *Ae. albopictus*) densities and the efficacy of control campaigns, such as the house index (HI: percentage of houses with at least one active breeding site), the container index (CI: percentage of containers with larvae), the Breteau index (BI: number of active breeding sites per 100 premises), and the ovitrap index (OI: the average proportion of ovitraps with mosquitoes) are widely used as standard empirical parameters in developing countries (WHO 1972; Ho et al. 2005). The same indices could also be potentially applied to other invasive species with similar oviposition habits, such as *Ae. atropalpus*, *Ae. japonicus*, *Ae. koreicus,* and *Ae. triseriatus*. However, results obtained using these indices are of limited value in European countries because of the differences in socio-economic and structural conditions that characterise human dwellings and the differences in the availability of breeding sites in public areas. Other indices that are more appropriate for European urban areas, devised from pupal demographic surveys (PDS) are the PPI (number of pupae/premise) and PHI (number of pupae/hectare), which define the mosquito density per unit area, applicable to both public and private domains (Carrieri et al. 2011). The pupal demographic survey (PDS) seems to be more appropriate for

epidemiological studies focusing on the estimation of the vector density transmission threshold (Focks & Chadee 1997; Focks 2003; Focks et al. 2007). The PDS exploits the strong correlation between the number of pupae and the number of adults in a defined area, based on the low incidence of natural mortality usually affecting the pupal stage.

Studies on the correlation between traditional indices and adult population densities show contradictory results: while some evidenced a good correlation between BI and both the larval and the adult densities, others found no correlation between traditional indices and the PHI or PPP (Tun-Lin et al. 1996). In a recent study conducted in Italy, a statistically significant correlation between PHI and the mean number of eggs/ovitrap was found (Carrieri et al. 2011a). Similarly, authors correlated the number of females/hectare, estimated on the basis of the number of sampled pupae, with the number of eggs. Finally, they suggested that the number of eggs estimated by means of ovitrap monitoring can be used to determine the mean number of biting females per unit area. Codeco et al. (2009) used a trap positivity index (TP: the proportion of positive traps) and an egg density index (MED: the ratio between the total number of eggs collected and the total number of traps) to compare differences between seasons per neighbourhood and to produce infestation maps.

It should be mentioned that sample size is of crucial importance for obtaining reliable data (Carrieri et al 2011a). Alexander et al. (2006) developed a method to be used in the design of surveys of mosquito pupae, for identifying the key container types from which the majority of adult dengue vectors emerge. A step-wise rule, based on the entropy of the cumulative data, was devised for determining the sample size needed, in terms of the number of houses positive for pupae, at which a pupal survey might reasonably be stopped.

Dispersal

Flight, flight ranges and dispersal are the parameters indicating the distance that mosquitoes are able to travel (actively, by itself; or passively, by human transportation) from their breeding places to search for sugar meal, mates, blood meal, resting places and oviposition sites. Dispersal to seek a host is epidemiologically important as it is the means by which female mosquitoes acquire and disseminate pathogens. Dispersal for oviposition is also relevant to disease transmission as it increases dispersal of potentially infected progeny (Watson 1967). Knowledge about the movements of adult mosquito vectors in endemic or epidemic areas in Europe is needed to understand disease transmission dynamics and to determine the areas for which to implement control measures to reduce/interrupt pathogen transmission (Marini et al. 2010).

The flight of mosquitoes is influenced by factors such as blood sources, availability of oviposition sites, weather (e.g. wind, humidity, temperature, rainfall), terrain, vegetation, housing characteristics (in urban environments) (Petrić 1989; Petrić et al. 1995; Honorio et al. 2003) and species-specific traits. Most mosquitoes will not take off, and if airborne, will attempt to land if wind speed is higher that their flight speed (in average 1 m/s).

During the early period of dispersal of *Ae. albopictus* in the USA, its presence appeared to be related to the proximity to interstate highways (Moore & Mitchell 1997). The postulated relationship between dispersal and major transportation routes would be expected for all invasive mosquito species transported largely by human activities, such as the commercial movement of used tyres for retreading (recapping) or recycling, ornamental plant trade, and individual, public and commercial transportation from infested areas. Once populations of the mosquito become established, local transport and active migration should disperse the mosquito throughout the surrounding area (Moore & Mitchell 1997).

Estimations of active mosquito dispersal are most frequently carried out by means of mark-release-recapture (MRR) studies, the effectiveness of which is strongly affected by the quantity of marked specimens released and the ability to carry out recapture over a large study area (Service 1999). Moreover, the availability of an effective recapture method may represent a serious limitation in MRR studies.

Results obtained in Brazil (Honorio et al. 2003) suggest that from an initial focus, one might expect house infestation in six days within a radius of almost 1 km^2 due to *Ae albopictus* and *Ae. aegypti* female dispersal (at least 800 metres in six days), independent of human activities such as transport and trade. Honorio et al. have shown that female dispersal by flight has the potential to be very important for the spread of dengue vectors, either between adjacent cities or in urban centres. Mean distance travelled (MDT) by *Ae. aegypti* in Rio de Janeiro varied from 57 to 122 metres, with a maximum of 263 metres (David et al. 2009). Another study in the same city revealed a maximum of 78.8 metres for smaller and 40.9 metres for larger females (Maciel-de-Freitas et al. 2007). In Queensland, Australia, *Ae. aegypti* females were recaptured, with the furthest being caught 200 metres from the release point, and the MDT was 78 metres (Russel et al. 2005). In Rome, a daily MDT average of 119 metres and a maximum range from 199 to 290 metres was recorded for *Ae albopictus* in urban areas, where dispersal was constrained by physical barriers (Marini et al. 2010).

The above evidence clearly illustrates that results obtained from MRR experiments cannot be generalised because results depend greatly on the ecological characteristics of the study sites. Moreover, the ecological factors affecting dispersal vary depending on the objectives of the mosquito dispersion (i.e. host seeking, resting or oviposition site seeking) which, in turn, implies different recapture approaches. Inconsistent results obtained in Australia, Brazil and Italy emphasise the importance of evaluation of dispersal capacity at local levels because each mosquito

population shows a different range of dispersal as a consequence of the species' intrinsic flight capability and its ecological setting. Preferably, surveys should be conducted for all host seeking, resting and ovipositing females, and also for males if SIT is going to be implemented.

2 Detailed methodologies

Types of invasive mosquito population measurements and statistic considerations

Generally, studies may be divided into extensive and intensive (Morris 1960, in: Southwood 2000). Extensive studies are carried out over larger areas than intensive studies. They are frequently used to provide information on distribution and abundance for surveillance or management programmes (e.g. monitoring the status of invasive species populations (Southwood 2000)). Current advances in remote sensing capability and geographical information system (GIS) software have provided great support to many studies in recent years. With regard to invasive species, extensive studies provide assessments of occurrence or distribution (VBORNET) and may guide the application of control measures and the assessment of their efficacy. In extensive surveys, an area will often be sampled once or several times per study period. The timing of the sampling and the way it is tailored to the life cycle of the invasive mosquito species is obviously of critical importance.

Intensive studies involve the frequent observation of the mosquito population. Usually information is acquired on the population size of successive developmental stages so that longevity, nuisance and/or vector capacity can be estimated. Intensive studies may focus on rather limited objectives, such as the determination of the level of containers (that support larval development) infestation, the dispersal, or the host preference of invasive species.

Population estimates can be further subdivided into three major types:

- **Absolute and related estimates** (comparisons in numbers)
  Population estimates can be expressed as absolute or relative, and in the form of population indices. For most animals (except the large ones that are easily observed and have small, countable populations, e.g. elephants, whales, and some birds, for which the overall population size may be given as a total number of individuals), numbers of absolute estimates are expressed as a density per unit area or volume (absolute population) or density per unit of the habitat, e.g. per water container or per host (population intensity) (Southwood 2000). Such estimates are given by census and distance sampling, mark-release-recapture, sampling of a known fraction of the habitat (e.g. transect and suction trapping, sampling from vertebrate hosts, dry sieving, and flotation of floodwater mosquito eggs), and removal sampling (using of different traps, e.g. baited traps, and converting the estimates to absolute terms, using specific correction factors) (Southwood 2000). As the level of the invasive species population is being related to breeding site and host availability, measuring the population intensity is often the type of data that are first obtained and more meaningful than an estimate of absolute density.
- **Relative estimates** (comparisons in space or time)
  In relative estimates, the numbers sampled cannot be expressed as density or intensity per area or habitat unit, and can only be used to compare data in space or time. Relative estimates are especially useful in assessing the species' relative density, dispersal, distribution, and host preference. Other applications lie in monitoring of environmental changes, the surveillance of MBD, and the evaluation of the efficacy of control measures. The methods employed are usually oriented to various forms of trapping, in which the number of caught individuals depends on a number of factors besides population density (Service 1999; Southwood 2000). Consequently, such methods should only be implemented by highly skilled technicians; data analysis should be carried out by specialists.
- **Population indices**
  If the mosquitoes are not counted, but their incidence in breading sites is recorded, the resulting estimate is a population index, which represents the third major type of population estimate.
  The traditional indices used to evaluate *Stegomyia* population (CI, HI, BI, OI) have some disadvantages when implemented in epidemiological studies (Focks & Chadee 1997). The CI only considers the percent of positive containers and not their absolute number (either per unit area, per premise, or per person). The HI is more accurate than the CI because it refers to the number of houses, but it is again limited because it does not account for the number of positive containers. The BI is the only index that combines data on positive containers with the density per premise (WHO 1972). The main limitation of the three indices is the lack of information referring to the real productivity of the containers, the way these indices describe the relation to the adult population size, and their applicability to the larger European cities (Carrieri et al. 2011).
  The transmission thresholds for dengue (one of the MBD threatening Europe) based on the standing yield of *Ae. aegypti* pupae per person (PPP, see Focks et al. 2007) were developed for use in the assessment of risk of transmission and to provide targets for the actual degree of suppression by type of breeding container required to prevent or eliminate transmission in source-reduction programmes. When coupled with field observations from PDS, it was possible for the first time for control specialists to know how important the

various types of containers in the environment are in terms of contributing to the transmission threshold (Focks & Chadee 1997; Focks et al. 2007; Carrieri et al. 2011).

There is no clear-cut division between relative and absolute methods of sampling. Absolute methods are rarely 100% efficient and relative methods can occasionally be corrected to give absolute density estimates. Relative methods are important in applied areas, such as IMS and MBD surveillance programmes, where most of the information available may be derived from egg, larvae and pupae sampling, and adult trapping. In mosquito surveillance, density is often difficult to calculate (except when an exhaustive surveillance programme is implemented (Albieri et al. 2010)) from sampling statistics because of changes in trapping intensity and/or sampling equipment following changes in financial support.

Despite the almost exclusive use of relative estimates in mosquito surveillance programmes, absolute values are of crucial importance for estimating longevity or studying survival rate and mortality factors, therefore laboratory studies and relative population estimates that should be corrected to absolute figures are highly recommended.

The statistical errors of obtained values are usually referred to as the fiducial limits (the estimate (x) being expressed as $x \pm y$, where y = fiducial limits). The fiducial limits are normally calculated for a probability level of 0.05, which means that there are only five chances in 100 that the range given by the fiducial limits does not include the true value. If more samples are taken, the limits will be narrower, but the estimate may not move closer to the actual value. The entomologist is often worried that some of the assumptions about sampling efficiency (e.g. whether the survey for breeding sites detected all active water containers in the district) may be incorrect. Most of them, quite correctly, believe that acquired estimates should be compared with another method that has different assumptions. If the estimates are of the same scale, then the researcher can have much greater confidence in the reliability of the result of this study. It is therefore sound practice to estimate the population parameters by more than one method. In the long run, more knowledge of the ecology of the animal may be gained by studying other areas, making other estimates, or taking further samples instead of struggling for a very high level of accuracy in each operation (Southwood 2000). Laughlin (1976, in: Southwood 2000) has suggested that the ecologist may be satisfied with a higher probability level (e.g. 0.2) and thus narrower fiducial limits for estimates based on more than one method because such estimates have a qualitative, biological assurance, which is further supported by the consistency of the data.

Mark-release-recapture (MRR) method

The MRR method is frequently used to estimate longevity and growth, population size and dispersal. If a sample from a population is marked, returned to the original population, and then, after complete mixing, re-sampled, the number of marked individuals in the second sample will have the same ratio to the total numbers in the second sample as the total of marked individuals originally released have to the total population. A basic prerequisite for the use of these methods is a technique for marking the animals so that they can be released unharmed and unaffected into the wild and recognised again on recapture (Southwood 2000). Fluorescent dyes are the most widely used marking technique in mosquito MRR experiments (Trpis and Hauserman 1986; Bellini et al 2010; Marini et al. 2010; Gu et al. 2011) but recently rubidium (Rb)-marked blood (Honório et al. 2003) or a mosquito strain whose natural infection of *Wolbachia* had been removed (Bellini et al. 2010) have been employed as well.

Longevity

The most frequently factors when estimating the longevity of adult mosquitoes in the laboratory are food availability (water, blood meal, sugar solution (Lee 1994; Xue et al. 2010)) and temperature (Löwenberg Neto & Navarro-Silva 2004; Delatte et al. 2009). It is worthwhile mentioning that larval density affects size and longevity directly (Miller & Thomas 1958, in: Southwood 2000). The impact of predicted rising temperatures on larval development and longevity is less obvious; rising temperatures can speed up larval development but also lead to a reduction in the body size of juveniles and hence reduce adult longevity.

Delatte et al. (2009) used the Weibull model as a classic nonlinear parametric model to describe the relation between death rate and time. This model is generally applied with a mortality rate increasing roughly exponentially, with increasing age at senescence. Löwenberg Neto & Navarro-Silva (2004) analysed data obtained in the laboratory by means of ANOVA for longevity, and chi-square test for survivorship linearity.

Blood feeding and resting behaviour

Host preference and blood feeding behaviour can be assayed outdoors or in the laboratory (Alonso et al. 2003) using olfactometer or cages of various construction and various hosts (Shirai et al. 2000). Using humans as a host is very important in the study of mosquito attractants, repellents, and host preference. However, mosquito bites cause potential medical problems because of hypersensitivity and perhaps secondary bacterial infection, even when using laboratory mosquitoes. Moreover, once a female mosquito has fed on human blood, it cannot be used in subsequent probing tests. Shirai et al. (2000) offered a solution to these problems by introducing a proboscis (mosquito mouth part) amputation technique.

Host-preference experiments conducted outdoors are based on host-baited traps of various design (Service 1999; Jaenson 1985; Becker et al. 1995), odour-baited traps (Costantini et al. 1993), or on blood meal analysis (Apperson et al. 2004; Molaei & Andreadis 2006; Richards et al. 2006). Hosts of blood-fed mosquitoes can be identified with an indirect enzyme-linked immunosorbent assay by using antisera made in rabbits for sera of animals that would commonly occur in certain habitats. Blood meals taken from birds can be identified to species by a polymerase chain reaction-heteroduplex assay (PCR-HDA) (Lee et al. 2002); blood meals from humans (including multiple blood meals taken from more than one human) can be identified by STR/PCR-DNA profiling technique, which involves amplification of three short tandem repeats loci (Chow-Shaffer et al. 2000; De Benedictis et al. 2003; Richards et al. 2006). Richards et al. (2006) also presented valuable methods for data processing, host feeding patterns, and host feeding indices calculation.

Assessing the nuisance thresholds for dominant mosquito species is of a great value for the evaluation of conventional control measures (Petrić et al. 1999; Becker et al. 2010) but estimation of a disease transmission threshold needs intensive sampling and expert data processing of, for example, the number of pupae per person (PPT; Focks et al. 2007). This usually involves deciding which seasonal estimates to use, what temperature to use, and what value for overall seroprevalence of virus antibody to use.

Gonotrophic cycle (the time females spend from finding a host to laying the eggs in nature, or from blood meal to egg laying in the laboratory) is another population parameter connected both with host-finding and blood-feeding but also with resting, digestion of blood, and oocyte maturation and oviposition. Its duration determines how many hosts a female will be feeding during its lifetime, which greatly influences the chances of finding an infectious host and transmitting a pathogen. Gu et al. (2006) divided the length of the gonotrophic cycle under natural conditions into three parts: (a) the time spent for host-seeking, i.e. starting with a blood meal in laboratory (Clements 1999); (b) resting, i.e. digestion and egg maturation time, and (c) oviposition time (seeking the site). The frequency of mosquitoes biting humans is estimated by Gu et al. (2006) as the ratio of the human blood index (HBI) to the length of the gonotrophic cycle.

Dispersal

While searching for a blood meal, some species of mosquitoes may fly close to the ground whilst others do not. This particularity of the species should be taken into account when choosing a trap type for adult sampling during MRR studies. Gu et al. (2006) assumed that the 'poor searcher' mosquito has a searching flight path of around 250 metres per day (all invasive species currently present in Europe belong to this group), whereas the 'good searcher' should be capable of searching up to 500 metres per day ('good' and 'poor' searcher represent two 'virtual' mosquitoes, representing different daily ranging abilities). This assumption can be incorporated in the estimation of the basic reproduction number (R_0 expressed the expected number of newly infected humans that will occur if one infected human is introduced into a totally susceptible human population), the most important measure of transmission dynamics.

In the case of endophilic species, marked mosquitoes can be efficiently recaptured by active aspiration in houses during their indoor resting phase (Harrington et al. 2005), but this approach is much less efficient for collecting exophilic mosquitoes resting outdoors (Facchinelli et al. 2008). Mouse-baited traps were used to assess the longevity and dispersal of male and female *Ae. albopictus* by MRR (Lacroix et al. 2009). Honorio et al. (2003) fed females with rubidium-marked blood and afterwards detected Rb in ovitrap-collected eggs by atomic emission spectrophotometry. Bellini et al. (2010) investigated the dispersal of *Ae. albopictus* males in urban localities in northern Italy by MRR techniques, recapturing the males on human hosts and while swarming. Marini et al. (2010) obtained a 4.3% recapture rate by using sticky traps in MRR experiments to study the dispersal of *Ae. albopictus* females in Rome, Italy. Traditionally, CDC backpack aspirators are used for recapturing resting females (Maciel de Freitas et al. 2008; David et al. 2009). A recently developed adult trap, MosquiTRAP, and others sticky traps are capturing oviposition-seeking females (Muir & Kay 1998; Russel et al. 2005; Maciel de Freitas et al. 2008; Rocha David et al. 2009; Marini et al. 2010), while the BG-Sentinel trap mainly samples host-seeking females (Maciel de Freitas et al. 2006; David et al. 2009).

Earlier dispersal studies involved the use of fluorescent pigments that had already been applied to a number of mosquito species in different habitats and with different dispersal behaviour (Trpis & Hauserman 1986; Russel et al. 2005; Bellini et al 2010; Marini et al. 2010; Gu et al. 2011). Other methods include rubidium-marked blood (Honório et al. 2003) and employing a mosquito strain whose natural infection of *Wolbachia* had been removed (Bellini et al. 2010). The mean distance travelled for *Wolbachia*-free males was significantly higher than for males marked with fluorescent powder. In the same, the paper authors characterised the dispersal pattern by mean distance travelled (MDT), maximum distance travelled (MAX), and flight range (FR), and presented useful procedures for data processing.

Key references

Albieri A, Carrieri M, Angelini P, Baldacchini F, Venturelli C, Mascali Zeo S, et al. Quantitative monitoring of *Aedes albopictus* in Emilia-Romagna, Northern Italy: cluster investigation and geostatistical analysis. Bull Insectology. 2010;63(2):209-16.

Alexander N, Lenhart AE, Romero-Vivas CM, Barbazan P, Morrison AC, Barrera R, et al. Sample sizes for identifying the key types of container occupied by dengue-vector pupae: the use of entropy in analyses of compositional data. Ann Trop Med Parasitol. 2006 Apr;100 Suppl 1:S5-S16.

Alonso WJ, Wyatt TD, Kelly DW. Are vectors able to learn about their hosts? A case study with *Aedes aegypti* mosquitoes. Mem Inst Oswaldo Cruz. 2003 Jul;98(5):665-72.

Apperson CS, Hassan HK, Harrison BA, Savage HM, Aspen SE, Farajollahi A, et al. Host feeding patterns of established and potential mosquito vectors of West Nile virus in the eastern United States. Vector Borne Zoonotic Dis. 2004 Spring;4(1):71-82.

Becker N, Petrić D, Zgomba M, Boase C, Madon M, Dahl C, et al. Mosquitoes and their control. Heidelberg, Dordrecht, New York: Springer; 2010.

Becker N, Zgomba M, Petrić D, Ludwig M. Comparison of carbon dioxide, octenol and a host-odour as mosquito attractants in the Upper Rhine Valley, Germany. Med Vet Entomol. 1995 Oct;9(4):377-80.

Bellini R, Albieri A, Balestrino F, Carrieri M, Porretta D, Urbanelli S, et al. Dispersal and survival of *Aedes albopictus* (Diptera: Culicidae) males in Italian urban areas and significance for sterile insect technique application. J Med Entomol. 2010 Nov;47(6):1082-91.

Bellini R, Calvitti M, Medici A, Carrieri M, Celli G, Maini S, editors. Use of the sterile insect technique against *Aedes albopictus* in Italy: First results of a pilot trial, pp. 505-515. Dordrecht, The Netherlands: Springer 2007.

Bellini R, Medici A, Carrieri M, Calvitti M, Cirio U, Maini S. Applicazione della tecnica del maschio sterile nella lotta contro *Aedes albopictus* in Italia: ottimizzazione delle fasi di allevamento massale e sessaggio. XIX Congresso Nazionale Italiano Entomologia; 2002 10-15 giugno; Catania; 2002. p. 993-8.

Carrieri M, Albieri A, Angelini P, Baldacchini F, Venturelli C, Zeo SM, et al. Surveillance of the chikungunya vector *Aedes albopictus* (Skuse) in Emilia-Romagna (northern Italy): organizational and technical aspects of a large scale monitoring system. J Vector Ecol. 2011 Jun;36(1):108-16.

Carrieri M, Angelini P, Venturelli C, Maccagnani B, Bellini R. *Aedes albopictus* (Diptera: Culicidae) population size survey in the 2007 Chikungunya outbreak area in Italy. I. Characterization of breeding sites and evaluation of sampling methodologies. J Med Entomol. 2011 Nov;48(6):1214-25.

Celedonio-Hurtado H, Liedo P, Aluja M, Guillen J, Berrigan D, Carey J. Demography of *Anastrepha ludens*, *A. obliqua* and *A. serpentina* (Diptera: Teprithidae) in Mexico. Florida Entomologist 1988;71(2):111-20.

Chadee DD, Huntley S, Focks DA, Chen AA. *Aedes aegypti* in Jamaica, West Indies: container productivity profiles to inform control strategies. Trop Med International Health. 2009;14(2):1-8.

Chow-Shaffer E, Sina B, Hawley WA, De Benedictis J, Scott TW. Laboratory and field evaluation of polymerase chain reaction-based forensic DNA profiling for use in identification of human blood meal sources of *Aedes aegypti* (Diptera: Culicidae). J Med Entomol. 2000 Jul;37(4):492-502.

Clements AN. The Biology of Mosquitoes, Sensory Reception and Behaviour. London: Chapman & Hall 1999.

Codeco CT, Honorio NA, Rios-Velasquez CM, Santos Mda C, Mattos IV, Luz SB, et al. Seasonal dynamics of *Aedes aegypti* (Diptera: Culicidae) in the northernmost state of Brazil: a likely port-of-entry for dengue virus 4. Mem Inst Oswaldo Cruz. 2009 Jul;104(4):614-20.

Conway GR, Trpis M, McClelland GAH. Population parameters of the mosquito *Aedes aegypti* (L.) estimated by mark-release-recapture in a suburban habitat in Tanzania. J Anim Ecol. 1974;43:289-304.

Costantini C, Gibson G, Brady J, Merzagora L, Coluzzi M. A new odour-baited trap to collect host-seeking mosquitoes. Parassitologia. 1993 Dec;35(1-3):5-9.

David MR, Lourenco-de-Oliveira R, Freitas RM. Container productivity, daily survival rates and dispersal of *Aedes aegypti* mosquitoes in a high income dengue epidemic neighbourhood of Rio de Janeiro: presumed influence of differential urban structure on mosquito biology. Mem Inst Oswaldo Cruz. 2009 Sep;104(6):927-32.

De Benedictis J, Chow-Shaffer E, Costero A, Clark GG, Edman JD, Scott TW. Identification of the people from whom engorged *Aedes aegypti* took blood meals in Florida, Puerto Rico, using polymerase chain reaction-based DNA profiling. Am J Trop Med Hyg. 2003 Apr;68(4):437-46.

Delatte H, Gimonneau G, Triboire A, Fontenille D. Influence of temperature on immature development, survival, longevity, fecundity, and gonotrophic cycles of *Aedes albopictus*, vector of chikungunya and dengue in the Indian Ocean. J Med Entomol. 2009 Jan;46(1):33-41.

Delatte H, Desvars A, Bouetard A, Bord S, Gimonneau G, Vourc'h G, et al. Blood-feeding behavior of *Aedes albopictus*, a vector of Chikungunya on La Reunion. Vector Borne Zoonotic Dis. 2010 Apr;10(3):249-58.

Dye C, Hasibeder G. Population dynamics of mosquito-borne disease: effects of flies which bite some people more frequently than others. Trans R Soc Trop Med Hyg. 1986;80(1):69-77.

Facchinelli L, Koenraadt CJ, Fanello C, Kijchalao U, Valerio L, Jones JW, et al. Evaluation of a sticky trap for collecting *Aedes (Stegomyia)* adults in a dengue-endemic area in Thailand. Am J Trop Med Hyg. 2008 Jun;78(6):904-9.

Fernandez Z, Forattini OP. [Survival of *Aedes albopictus*: physiological age and reproductive history]. Rev Saude Publica. 2003 Jun;37(3):285-91.

Focks DA. A review of entomological sampling methods and indicators for dengue vectors. World Health Organization on behalf of the Special Programme for Research and Training in Tropical Diseases. 2003.

Focks DA, Bangs MJ, Church C, Juffrie M, Nalim S. Transmission thresholds and pupal/demographic surveys in Yogyakarta, Indonesia for developing a dengue control strategy based on targeting epidemiologically significant types of water-holding containers. Dengue Bulletin. 2007;31:83-102.

Focks DA, Chadee DD. Pupal survey: an epidemiologically significant surveillance method for *Aedes aegypti*: an example using data from Trinidad. Am J Trop Med Hyg. 1997 Feb;56(2):159-67.

Gu W, Muller G, Schlein Y, Novak RJ, Beier JC. Natural plant sugar sources of *Anopheles* mosquitoes strongly impact malaria transmission potential. PLoS One. 2011;6(1):e15996.

Gu W, Regens JL, Beier JC, Novak RJ. Source reduction of mosquito larval habitats has unexpected consequences on malaria transmission. Proc Natl Acad Sci U S A. 2006 Nov 14;103(46):17560-3.

Harrington LC, Scott TW, Lerdthusnee K, Coleman RC, Costero A, Clark GG, et al. Dispersal of the dengue vector *Aedes aegypti* within and between rural communities. Am J Trop Med Hyg. 2005 Feb;72(2):209-20.

Heffernan JR, J. SR, M. WL. Perpective on the basic reproductive ratio. J R Soc Interface. 2005;2:281-93.

Hethcote HW. The mathematics of infectious diseases. SIAM Rev. 2000;42(4):599-653.

Ho CM, Lin MW, Teng HC, Lai MH, Lin TS, Hsu EL, et al. Surveillance for dengue fever vectors using ovitraps at Kaohsiung and Tainan in Taiwan. Formosan Entomol. 2005;25:159-74.

Honório NA, Silva Wda C, Leite PJ, Goncalves JM, Lounibos LP, Lourenco-de-Oliveira R. Dispersal of *Aedes aegypti* and *Aedes albopictus* (Diptera: Culicidae) in an urban endemic dengue area in the State of Rio de Janeiro, Brazil. Mem Inst Oswaldo Cruz. 2003 Mar;98(2):191-8.

Jaenson TG. Attraction to mammals of male mosquitoes with special reference to *Aedes diantaeus* in Sweden. J Am Mosq Control Assoc. 1985 Jun;1(2):195-8.

Jones JH. Notes on R0. Department of Anthropological Sciences, Stanford University. 2007:1-19.

Kosova J. Longevity Studies of Sindbis Virus Infected *Aedes albopictus*. The Osprey Journal of Ideas and Inquiry. 2003;All Volumes (2001-2008):21-31.

Lacroix R, Delatte H, Hue T, Reiter P. Dispersal and survival of male and female *Aedes albopictus* (Diptera: Culicidae) on Reunion Island. J Med Entomol. 2009 Sep;46(5):1117-24.

Lambrechts L, Scott TW, Gubler DJ. Consequences of the expanding global distribution of *Aedes albopictus* for dengue virus transmission. PLoS Negl Trop Dis. 2010;4(5):e646.

Lee SH. Bloodsucking, oviposition and longevity of *Aedes albopictus* (Skuse) (Diptera: Culicidae). Chinese J. Entomol. 1994;14:217-31.

Lee JH, Hassan H, Hill G, Cupp EW, Higazi TB, Mitchell CJ, et al. Identification of mosquito avian-derived blood meals by polymerase chain reaction-heteroduplex analysis. Am J Trop Med Hyg. 2002 May;66(5):599-604.

Löwenberg Neto P, Navarro-Silva MA. Development, Longevity, Gonotrophic Cycle and Oviposition of *Aedes albopictus* Skuse (Diptera: Culicidae) under Cyclic Temperatures. Neotropical Entomology. 2004;33(1):29-33.

Maciel-De-Freitas R, Codeco CT, Lourenco-De-Oliveira R. Body size-associated survival and dispersal rates of *Aedes aegypti* in Rio de Janeiro. Med Vet Entomol. 2007 Sep;21(3):284-92.

Maciel-de-Freitas R, Eiras AE, Lourenco-de-Oliveira R. Field evaluation of effectiveness of the BG-Sentinel, a new trap for capturing adult *Aedes aegypti* (Diptera: Culicidae). Mem Inst Oswaldo Cruz. 2006 May;101(3):321-5.

Maciel-de-Freitas R, Peres RC, Alves F, Brandolini MB. Mosquito traps designed to capture *Aedes aegypti* (Diptera: Culicidae) females: preliminary comparison of Adultrap, MosquiTRAP and backpack aspirator efficiency in a dengue-endemic area of Brazil. Mem Inst Oswaldo Cruz. 2008 Sep;103(6):602-5.

Marini F, Caputo B, Pombi M, Tarsitani G, della Torre A. Study of *Aedes albopictus* dispersal in Rome, Italy, using sticky traps in mark-release-recapture experiments. Med Vet Entomol. 2010 Dec;24(4):361-8.

McCall PJ, Kelly DW. Learning and memory in disease vectors. Trends in Parasitol. 2002;18:429-33.

Molaei G, Andreadis TG. Identification of avian- and mammalian-derived bloodmeals in *Aedes vexans* and *Culiseta melanura* (Diptera: Culicidae) and its implication for West Nile virus transmission in Connecticut, U.S.A. J Med Entomol. 2006 Sep;43(5):1088-93.

Moore CG, Mitchell CJ. *Aedes albopictus* in the United States: ten-year presence and public health implications. Emerg Infect Dis. 1997 Jul-Sep;3(3):329-34.

Muir LE, Kay BH. *Aedes aegypti* survival and dispersal estimated by mark-release-recapture in northern Australia. Am J Trop Med Hyg. 1998 Mar;58(3):277-82.

Niebylski ML, Craig GB, Jr. Dispersal and survival of *Aedes albopictus* at a scrap tire yard in Missouri. J Am Mosq Control Assoc. 1994 Sep;10(3):339-43.

Nur Aida H, Abu Hassan A, Nurita AT, Che Salmah MR, Norasmah B. Population analysis of *Aedes albopictus* (Skuse) (Diptera:Culicidae) under uncontrolled laboratory conditions. Tropical Biomedicine 2008;25(2):117–25.

Petrić D. Seasonal and daily activity of Culicidae in Vojvodina province (Yugoslavia). PhD Thesis. Novi Sad: University of Novi Sad; 1989.

Petrić D, Zgomba M, Bellini R, Veronesi R, Kaiser A, Becker N. Validation of CO2 trap data in three European regions. In: Boase C, editor. 3rd International Conference of Insect Pests in the Urban Environment; 1999; Prague, Czech Republic; 1999. p. 437-45.

Petrić D, Zgomba M, Ludwig M, Becker N. Dependence of CO2-baited suction trap captures on temperature variations. J Am Mosq Control Assoc. 1995 Mar;11(1):6-10.

Richards SL, Ponnusamy L, Unnasch TR, Hassan HK, Apperson CS. Host-feeding patterns of *Aedes albopictus* (Diptera: Culicidae) in relation to availability of human and domestic animals in suburban landscapes of central North Carolina. J Med Entomol. 2006 May;43(3):543-51.

Rocha David M, Lourenço-de-Oliveira R., Maciel de Freitas R. (2009) Container productivity, daily survival rates and dispersal of *Aedes aegypti* mosquitoes in a high income dengue epidemic neighbourhood of Rio de Janeiro: presumed influence of differential urban structure on mosquito biology. Mem Inst Oswaldo Cruz, Rio de Janeiro, 2009; 104(6):927-32.

Russell RC, Webb CE, Williams CR, Ritchie SA. Mark-release-recapture study to measure dispersal of the mosquito *Aedes aegypti* in Cairns, Queensland, Australia. Med Vet Entomol. 2005 Dec;19(4):451-7.

Service MW. Mosquito ecology: field sampling methods. London: Elsevier 1993.

Shirai Y, Kamimura K, Seki T, Morohashi M. Proboscis amputation facilitates the study of mosquito (Diptera: Culicidae) attractants, repellents, and host preference. J Med Entomol. 2000 Jul;37(4):637-9.

Smith DL, McKenzie FE. Statics and dynamics of malaria infection in *Anopheles* mosquitoes. Malar J; 2004;3:13.

Smith DL, McKenzie FE, Snow RW, Hay SI. Revisiting the basic reproductive number for malaria and its implications for malaria control. PloS Biology. 2007;5(3):531-42.

Southwood TRE, Henderson BE. Ecological methods: Blackwell Publishing 2000.

Trpis M, Hausermann W. Dispersal and other population parameters of *Aedes aegypti* in an African village and their possible significance in epidemiology of vector-borne diseases. Am J Trop Med Hyg. 1986 Nov;35(6):1263-79.

Tsuda Y, Wada Y, Takagi M. Parous rate as a function of basic population parameters of mosquitoes. Trop Med 1991;33(3):47-54.

Tun-Lin W, Kay BH, Barnes A, Forsyth S. Critical examination of *Aedes aegypti* indices: correlations with abundance. Am J Trop Med Hyg. 1996 May;54(5):543-7.

Watson MS. Aedes (Stegomyia) albopictus (Skuse): a literature review. Miscellaneous publication 22. Frederick, Maryland 21701: Department of the Army, Fort Derrick 1967:41 pp.

WHO. A system of world-wide surveillance for vectors. Weekly Epidemiological Record. World Health Organization 1972:73–84.

Xue RD, Barnard DR, Muller GC. Effects of body size and nutritional regimen on survival in adult *Aedes albopictus* (Diptera: Culicidae). J Med Entomol. 2010 Sep;47(5):778-82.

Annex 6: Complementary information on methods for pathogen screening in field collected mosquitoes

Table A: Methods for handling, preparation and storage of mosquito samples according to the methods for assessing the infection status of mosquitoes or identifying the pathogens.

Pathogen identification method	Strengths	Weaknesses	Mosquito handling and preparation	Sample storage
Pan-flavivirus PCR	Highly sensitive, optimal cost/work rate	Not specific	Transport alive or in dry ice; rapid identification on chill table	Storing at low temperature or RNA stabilisation buffer
Pan-alphaviruses	Highly sensitive, optimal cost/work rate	Not specific	Transport alive or in dry ice; rapid identification on chill table	Storing at low temperature or RNA stabilisation buffer
Pan-orthobunyaviruses	Highly sensitive, optimal cost/work rate	Not specific	Transport alive or in dry ice; rapid identification on chill table	Storing at low temperature or RNA stabilisation buffer
Discriminating PCR analysis	Highly sensitive, highly specific	No virus availability for culturing	Transport alive or in dry ice; rapid identification on chill table	Storing at low temperature or RNA stabilisation buffer
Virus genome detection: RT-PCR, Taq Man, VecTest, RAMP	Easy and fast to use	Sensitivity and specificity not optimal	Transport alive or in dry ice; rapid identification on chill table	Storing at low temperature
Virus isolation using mammalian or insect cells: Vero, BHK-21, RK-13, C6/36	Virus availability for full genome description	Time-consuming	Transport alive or in dry ice; rapid identification on chill table	Storing at low temperature
Dirofilaria PCR	Highly sensitive		Transport alive; kept refrigerated until rapid identification	Storing frozen (<0° C)

In the context of IMS, the main pathogens to be targeted for surveillance are the dengue (1-2-3-4) and chikungunya viruses. As dengue belongs to the flavivirus group, which also includes a number of other arboviruses, it is recommended to use a pan-flavivirus PCR testing system in order to test for flavivirus, and then perform a species-specific PCR if the generic test produces a positive result. Chikungunya is an alphavirus for which specific PCR primers are available.

Mosquito trapping collection systems are described in Annex 4.

Collected mosquitoes should be brought to the lab within 24 hours, alive or frozen (i.e. using dry ice) and then sorted, identified and pooled by species (using a refrigerated table if frozen). Pooled samples should be labelled with time, day and collection site. Samples should be stored at least at -80 °C for long-term storage, or -20 °C for shorter periods, avoiding freezing and thawing. If freezing is not possible, RNA stabilisation buffer ('RNAlater' tissue collection storage solution), which allows storage at room temperature, is a proven alternative. Generic PCR-positive samples may be checked for virus isolation, which requires splitting the samples before further processing. Storage in RNA-stabilisation buffer will hinder successful virus isolation.

Pool size is usually 10 to 200 individuals, but this depends on the collection method, the population density, the expected infection rate, and the sensitivity of the screening method. When the infection rate is expected to be very low, larger pools might be considered to reduce costs.

Data presentation for infected mosquitoes

Data on arboviruses in field-collected mosquitoes are useful when tracking virus activity; they can also provide an index of vector capacity for specific arboviruses. The standard measure of mosquito-based arbovirus surveillance (as used by the ArboNET system operated by the US Centers for Disease Control and Prevention (CDC);

http://www.cdc.gov/ncidod/dvbid/westnile/index.htm) is the number of positive mosquito pools found in collections of a particular mosquito species in a defined geographical area, and in a defined time period.

While the absolute number of positive pools provides valuable information, it does not provide an index of virus prevalence for the vector population. The proportion of the mosquito population carrying the virus should ideally be expressed as the infection rate (IR). At a regional/provincial level, weekly tracking of mosquito IR can provide an important predictive indicator of transmission activity associated with elevated disease risk to humans. At the European level, the comparison of indices between outbreak areas may help to understand the arbovirus transmission patterns.

Estimates of the IR are usually presented as the number of infected mosquitoes per 1 000 tested. The simplest estimate for a defined period and surveillance area, the minimum infection rate (MIR), is calculated as: ([number of positive pools /total specimens tested] x 1000). The MIR assumes that a positive pool contains only one infected mosquito, which may be invalid when infection rates are high. When infection rates are high or sample sizes are small, a more accurate estimate of IR may be obtained by using a maximum likelihood estimate of the infection rate.

A user-friendly software application was developed at CDC for calculating IR (i.e. infection prevalence) estimates from mosquito pool data, using methods that do not require the assumption used in the MIR calculation. This programme also includes the calculation of confidence intervals which reflect, in part, the sample sizes used in the calculations. The confidence intervals provided (or any other uncertainty measure) are needed to interpret the precision of the IR estimate. This application is written as an Excel add-in and can be downloaded at http://www.cdc.gov/ncidod/dvbid/westnile/software.htm. It computes point and confidence interval estimates of IRs, using data from pooled samples, where pool sizes may differ. Bias-corrected likelihood methods are used to estimate infection rate, and a skew-corrected score confidence interval is computed by default. The documentation provided describes traditional methods for calculating the MIR for comparison.

Source: www.cdc.gov

Key references

Angelini P, Tamba M, Finarelli AC, Bellini R, Albieri A. West Nile virus circulation in Emilia-Romagna, Italy: the integrated surveillance system 2009. Euro Surveill. 2010;16(15):11-5.

Biggerstaff BJ. PooledInfRate, Version 3.0: a Microsoft® Excel® Add-In to compute prevalence estimates from pooled samples. Fort Collins, CO, USA: CDC 2006.

CDC Guidelines for Surveillance, Prevention, & Control. 3th revision. 6: Fort Collins CO 2003: available at: http://www.cdc.gov/ncidod/dvbid/westnile/resources/wnv-guidelines-aug-2003.pdf.

Chisenhall DM, Vitek CJ, Richards SL, Mores CN. A method to increase efficiency in testing pooled field-collected mosquitoes. J Am Mosq Control Assoc. 2008;24:311-4.

Dottori M. Arboviral survey of mosquitoes in two northern Italian regions in 2007 and 2008. Vector Borne Zoonotic Dis. 2010 Nov;10(9):875-84.

Gu W, Lampman R, Novak RJ. Assessment of arbovirus vector infection rates using variable size pooling. Med Vet Entomol. 2004; 18:200-4.

Gu W, Lampman R, Novak RJ. Problems in Estimating Mosquito infection Rates using minimum infection rate. Entomological Society of America 2003;40(5):595-6.

Gu W, Novak RJ. Short report: detection probability of arbovirus infection in mosquito populations. Am. J. Trop. Med. Hyg. 2004;71(5):636-8.

Gu W, Unnasch TR, Katholi CR, Lampman R, Novak RJ. Fundamental issues in mosquito surveillance for arboviral transmission. Trans. R. Soc. Trop. Med. Hyg. 2008;102:817-22.

Mavale M, Sudeep A, Gokhale M, Hundekar S, Parashar D, Ghodke Y, et al. Persistence of viral RNA in chikungunya virus-infected *Aedes aegypti* (Diptera: Culicidae) mosquitoes after prolonged storage at 28 °C. Am J Trop Med Hyg. 2012;86(1):178-80.

Scaramozzino N, Crance JM, Jouan A, DeBriel D A, Stoll F, et al. Comparison of flavivirus universal primer pairs and development of a rapid, highly sensitive heminested reverse transcription-PCR assay for detection of flaviviruses targeted to a conserved region of the NS5 gene sequences. J Clin Microbiol. 2001;39:1922-7.

Sutherland GL, Nasci RS. Detection of West Nile virus in large pools of mosquitoes. J Am Mosq Control Assoc. 2007;23:389-95.

Turrell MJ, Spring AR, Miller MK, Cannon CE. Effect of holding conditions on the detection of West Nile viral RNA by reverse transcriptase-polymerase chain reaction from mosquito (Diptera: Culicidae) pools. J. Med. Entomol. 2002;39:1-3.

Whatts KJ, Courteny CH, Reddy GR. Development of a PCR- and probe-based test for the sensitive and specific detection of the dog heartworm, *Dirofilaria immitis*, in its mosquito intermediate host. Mol. Cell. Probes 1999;13:425-30.

Xie H, Bain O, Williams SA. Molecular phylogenetic studies on filarial parasites based on 5S ribosomal spacer sequences. Parasite 1994;1:141-51.

Annex 7: Complementary information on measurement of environmental parameters

Environmental factors play an important role in determining the colonisation process of IMS, as well as in shaping the population size and the mosquito-borne disease transmission risk. These parameters are all environment-borne, i.e. parameter values are first and foremost dependent on the environmental and climatic conditions, and mosquitoes have to adapt to (e.g. temperature, blood and nectar availability, breeding site availability, etc.), whereas mosquito population parameters (see Chapter 2.3 and Annex 5) are primarily determined by the IMS population itself, i.e. mosquito-borne or internal/intrinsic (longevity, survival rates, host finding, host preference, dispersal, etc.).

Larval breeding sites productivity and vector control efficiency

The density and quality of larval breeding sites are the primary function of the landscape (natural) and human population (cultural) characteristics in a particular environment (urban, semirural, rural). The success of IMS colonisation for a territory depends to a large degree on the availability and density of breeding sites, and if IMS are introduced, the absence/presence of breeding sites will prevent/help establishment.

Larval breeding sites may be identified and classified based on their characteristics and their productivity for a defined IMS. This can be performed by inspection of breeding sites and collection of mosquitoes (with a dipper or an aquatic net: see Chapter 2.1 and Annex 3), applying a larval or pupal index (the mean number of larvae/pupae per container type; see Focks 2003). Despite the use of simple equipment, this task needs to involve highly trained and skilled professionals with profound knowledge of both targeted environment and IMS behaviour (adult oviposition habits, larval and pupal defensive behaviour, etc.). This requires a high level of manpower but this effort is indispensable for the proper application of control measures.

Mosquito control methods aim at rendering the environment unsuitable for mosquito breeding by applying versatile control measures (biological, chemical, physical). Methods for the evaluation of IMS control quality and efficacy assess the reduction of larvae/pupae per treated breeding site or the reduction of adult mosquitoes (both to measure efficacy of larval and adult control). Reduction of juveniles can be assessed based on the same method as described above, except in cases when insect growth regulators (IGR) are used: larvae should then be brought to the laboratory to have the adult emergency rate recorded. Presence and reduction of adult mosquitoes can be estimated by comparing the number of mosquitoes that are sampled with an adult trap (number of females/trap/night) or with human baiting (number of females/man/15mn) before and after the treatment. For a reliable assessment of the reduction level, untreated plots with mosquito abundance similar to the treated area should be selected and the same method of sampling/trapping applied. Mosquito abundance is best monitored three days before and three days after the treatment because of likely variations in the number of adult mosquitoes. Oviposition traps can be used to assess treatment efficacy in case of *Ae. albopictus*. In addition to the assessment of the efficacy of applied measures, a quality check of the control method and procedure may be performed, preferably by an independent external team, in order to review the quality of the performance of the control measures (choice of treatment sites and methods, quality of the performance itself, resistance management, prevention of environmental and health impact, etc.).

Mosquitoes and climate and environmental change

National communication reports from most European countries referring to the United Nations Framework Convention on Climate Change (UNFCCC) emphasise a need for the development of climate change mitigation and adaptation strategies. In the area of infectious disease, a key adaptation strategy will be the improved surveillance and monitoring of vector-borne diseases, supplemented by research on whether and how VBD are influenced by meteorological patterns and climate change. Additional interdisciplinary research on interactions with other risk factors would also be helpful.

Drivers for the emergence of infectious diseases include human demographics (e.g. the growth of megacities), international movement of people (travellers and refugees), the smuggling of wildlife, the trade of animals, inter-continental transport of disease vectors (e.g. trade in used tyres and certain ornamental plants) and various other aspects of globalisation. The drivers of meteorological and climate change are also of growing international and European-focused interest (Semenza & Menne 2009; ECDC 2009). Projected increases in air temperature are predicted to have an impact on poikilotherm species (whose body temperature depends on the ambient temperature), but also on insects that pose a threat to human health. The responses of IMS to these changes (in addition to physiological changes such as the potential for increased vector capacity) could lead to an expansion of colonised areas and the invasion of new sites. Warmer cities could favour mosquito breeding and, along with

higher air temperatures, shorten extrinsic incubation periods, e.g. for the urban IMS vector *Ae. aegypti* (Wilson & Lush 2010). Data on *Culex* spp*.* mosquitoes, vectors of West Nile virus (WNV), and meteorological factors indicate that higher mosquito populations in a given month may be associated with higher air temperatures and precipitation in the preceding month. Similarly, a study that examined the emergence of WNV in British Colombia, Canada, after a spread westward across the continent (Roth et al. 2010) suggests that higher average air temperatures, low snow cover and consequently reduced stream flows may have caused the observed increase in *Cx. tarsalis* populations, which facilitated viral amplification and spillover into human and equine populations. The overall pattern of the current studies on MBD suggests expanded ranges for disease incidence. Meteorological and climate change factors were identified as drivers for some of these patterns, but it is clear that many other factors are involved and may be more important. It is likely that similar scenarios could result in a geographic redistribution of other transmissible diseases and their insect vectors, which will be shaped by the ability of the insects to adapt to environmental changes caused by various factors.

All IMS threatening Europe are container breeders closely connected to urban and peri-urban habitats, where both human and animal hosts are plentiful. Peri-urbanisation occurs when urban regions begin to permeate into neighbouring rural regions. In 2011, the world's urban population exceeded the number of those living in rural areas, and by 2030, some two thirds of the world's people will be living in cities (UN projections 2008), potentially improving reproduction of IMS. New research results from the FP6 PLUREL project show that urban development is by far the most rapidly expanding land use change in Europe, rapidly continuing at 0.5 to 0.7 % per year, which is more than ten times higher than any other land use change.

The adoption of urban lifestyles in rural regions, and likewise rural activities such as farming in urban areas, has driven the growth of peri-urban agriculture, merging the agricultural markets of both settings. Urban agriculture is increasingly being recognised by public health professionals, urban planners, community organisations, and policy-makers as a valuable tool for economic development, preservation or production of green space, and improvement of food security (Brown & Jameton 2000).The benefits are many in the context of climate adaptation, economic alleviation and self-sustenance, but urban agriculture presents also challenges for human and animal health, including the increase of IMS breeding pools and the hazard of zoonotic diseases.

Increased trade and travel promote the transport of IMS eggs in goods (used tyres, Lucky Bamboo plants) and IMS adults in vehicles (cars, trucks, boats, airplanes), as well as pathogens in infected travellers. Human movement is a critical behavioural factor underlying observed patterns of MBD spread because movement determines exposure to vectors, i.e. bites from infected IMS and transmission of pathogens. Stoddard et al. (2009) estimated a dengue reproduction rate (R_0) of 1.3 when exposure is assumed to occur only in the home, as opposed to 3.75 when exposure occurs at multiple locations, e.g. during visits to markets and friends. Interestingly, their model predicted little correspondence between vector abundance and estimated R_0 when movement is taken into account. The authors illustrate the importance of human movement for understanding and predicting the dynamics of MBD and encourage the investigation of human movement and disease, primarily by reviewing methods for studying human movement and proposing key parameters for designing such a study.

Abandonment of farms encourages a tree-hole breeding mosquito (*Anopheles plumbeus*, potential vector of malaria) to invade new habitats and breed in abandoned manure tanks, thus becoming a container-breeding mosquito, similar to IMS. Increased irrigation to combat drought may provide new breeding sites for IMS. On the other hand, the reduced availability of aquatic habitats can significantly affect population dynamics and the vector capacity of mosquitoes.

Monitoring of meteorological parameters

A mosquito's flight is influenced by many factors, including the availability of blood sources and oviposition sites, weather (e.g. wind, humidity, temperature, rainfall), terrain, vegetation and housing characteristics (Honorio et al. 2003).

Bellini et al. (2010) demonstrated that low relative humidity, high temperatures, and intense solar radiation negatively influenced the mean flying distance and reduced the dispersion homogeneity. In hot and dry summer conditions, male mosquitoes showed reduced dispersal and sought shade.

The dispersal ability of a given species depends on the weather conditions during the searching period and the characteristics of the study locality. In urban areas, important factors include the vegetation type, its abundance and distribution; and the shape and position of buildings, squares, and main roads (Beier et al. 1982; Muir & Kay 1998; Reisen et al. 2003; Russell et al. 2005).

Temperature (seasonal averages, altitudinal variation) and precipitation (quantity, seasonal distribution pattern, water management habits of the human population) are crucial factors influencing the risk of establishment of IMS in an area and should be included in any risk modelling process.

Larval density directly affects mosquito size and longevity (Miller & Thomas 1958, in: Southwood 2000), as does an increase in temperature which may speed up development (but also reduces the juvenile body size of the mosquito,

which affects adult longevity). Evidence is accumulating that fluctuations in the weather affect the size of the mosquito population; it is therefore important to include meteorological parameters in any analysis of fertility, longevity, dispersal and vector capacity of local populations.

Meteorological parameters should be continually monitored for local populations, especially in countries with a wide range of temperatures (with different regional climates and a broad altitudinal range). In such countries, monitoring and recording meteorological parameters are of crucial importance to understand spread and other aspects of IMS vector populations.

Historical records of temperature and other meteorological data are available for many locations. These databases should be extensively used for the analysis of the IMS populations. However, portable meteorological stations are useful for more precise measurements at locations that are far from the main monitoring points of national meteorological institutions. Medium resolution satellite imagery may also provide valuable meteorological data.

Monitoring changes in urban habitats

Gu et al. (2006) found that reduction of aquatic habitats through environmental management mitigates malaria transmission by reducing breeding sites and the emergence of host-seeking mosquitoes, and by increasing the amount of time required for vectors to locate oviposition sites. This applies especially when aquatic habitats are scarce and the mosquito's flight ability is limited (which is true for all IMS threatening Europe). Focks and Chadee (1997) and Focks et al. (2007) were able to determine transmission thresholds for dengue based on the standing yield of *Ae. aegypti* pupae per person (PPP), which made it possible to set reduction targets – by type of breeding container – in order to prevent or eliminate transmission in source-reduction programmes. When coupled with field observations from pupal demographic surveys, it is possible for control specialists to assess the significance of the various types of containers in the environment in terms of contribution to the transmission threshold (Focks et al. 2007). However, the results of source reduction on mosquito oviposition have largely been neglected in the evaluations of environment management programmes. The characterisation and mapping of breeding sites in urban environments from the beginning of the colonisation is very useful for both entomological and epidemiological surveys and should not be neglected (see Chapter 2.6). Mapping can be done at a very high spatial resolution (up to 1 metre spatial resolution) using satellite data. Several satellites carry sensors that have dedicated electro-magnetic channels which could be used to characterise urban habitats – a potentially relevant factor when implementing control measures and efficiency assessments. Gu & Novak (2009) used an agent-based model to track the status and movement of individual mosquitoes; they show that the elimination of habitats within 100 m, 200 m and 300 m of surrounding houses resulted in reductions of 13%, 91% and 94% in malaria incidence; compared with -3%, -19% and -44% for the corresponding conventional interventions. These findings indicate that source reduction might not, as previously thought, require coverage of extensive areas and that the distance to human homes can be used for habitat targeting. The authors also emphasise the importance of acquiring and archiving of data about breeding sites/container types used by different IMS.

Mitigating the impact of heat waves on urban inhabitants encompasses the establishment of more green spaces that may provide both breeding sites and nectar sources for foraging IMS. Russel et al. (2005) show that dispersal of IMS in an urban environment is not random, and that it may be possible to maximise vector control by taking into account ecological factors that affect the direction of the flight of female mosquitoes.

Key references

Beier JC, Berry WJ, Craig GB, Jr. Horizontal distribution of adult *Aedes triseriatus* (Diptera: Culicidae) in relation to habitat structure, oviposition, and other mosquito species. J Med Entomol. 1982 May 28;19(3):239-47.

Bellini R, Albieri A, Balestrino F, Carrieri M, Porretta D, Urbanelli S, et al. Dispersal and survival of *Aedes albopictus* (Diptera: Culicidae) males in Italian urban areas and significance for sterile insect technique application. J Med Entomol. 2010 Nov;47(6):1082-91.

Brown KH, Jameton AL. Public Health Implication of Urban Agriculture. Journal of Public Health Policy. 2000;21(1):20-39.

ECDC. Development of *Aedes albopictus* risk maps: Stockholm, ECDC Technical report 2009. Available from: http://www.ecdc.europa.eu/en/publications/Publications/0905_TER_Development_of_Aedes_Albopictus_Risk_Maps.pdf

Focks DA. A review of entomological sampling methods and indicators for dengue vectors. World Health Organization on behalf of the Special Programme for Research and Training in Tropical Diseases. 2003.

Focks DA, Bangs MJ, Church C, Juffrie M, Nalim S. Transmission thresholds and pupal/demographic surveys in Yogyakarta, Indonesia for developing a dengue control strategy based on targeting epidemiologically significant types of water-holding containers. Dengue Bulletin. 2007;31:83-102.

Focks DA, Chadee DD. Pupal survey: an epidemiologically significant surveillance method for *Aedes aegypti*: an example using data from Trinidad. Am J Trop Med Hyg. 1997 Feb;56(2):159-67.

Gu W, Novak RJ. Agent-based modelling of mosquito foraging behaviour for malaria control. Trans R Soc Trop Med Hyg. 2009 Nov;103(11):1105-12.

Gu W, Regens JL, Beier JC, Novak RJ. Source reduction of mosquito larval habitats has unexpected consequences on malaria transmission. Proc Natl Acad Sci U S A. 2006 Nov 14;103(46):17560-3.

Honório NA, Silva Wda C, Leite PJ, Goncalves JM, Lounibos LP, Lourenco-de-Oliveira R. Dispersal of *Aedes aegypti* and *Aedes albopictus* (Diptera: Culicidae) in an urban endemic dengue area in the State of Rio de Janeiro, Brazil. Mem Inst Oswaldo Cruz. 2003 Mar;98(2):191-8.

Muir LE, Kay BH. *Aedes aegypti* survival and dispersal estimated by mark-release-recapture in northern Australia. Am J Trop Med Hyg. 1998 Mar;58(3):277-82.

Reisen WK, Lothrop HD, Lothrop B. Factors influencing the outcome of mark-release-recapture studies with *Culex tarsalis* (Diptera: Culicidae). J Med Entomol. 2003 Nov;40(6):820-9.

Roth D, Henry B, Mak S, Fraser M, Taylor M, Li M, et al. West Nile virus range expansion into British Columbia. Emerg Infect Dis. 2010 Aug;16(8):1251-8.

Russell RC, Webb CE, Williams CR, Ritchie SA. Mark-release-recapture study to measure dispersal of the mosquito *Aedes aegypti* in Cairns, Queensland, Australia. Med Vet Entomol. 2005 Dec;19(4):451-7.

Semenza JC, Menne B. Climate change and infectious diseases in Europe. Lancet Infect Dis. 2009 Jun;9(6):365-75.

Southwood TRE, Henderson BE. Ecological methods: Blackwell Publishing 2000.

Stoddard ST, Morrison AC, Vazquez-Prokopec GM, Paz Soldan V, Kochel TJ, et al. The role of human movement in the transmission of vector-borne pathogens. PLoS Negl Trop Dis. 2009;3(7):e481.

Wilson N, Lush D, Baker MG. Meteorological and climate change themes at the 2010 International Conference on Emerging Infectious Diseases. Euro Surveill. 2010;15(30).

Annex 8: Complementary information on data management and analysis

Figure A: ERD (entity relation diagram) of a database specifically designed for vector surveillance

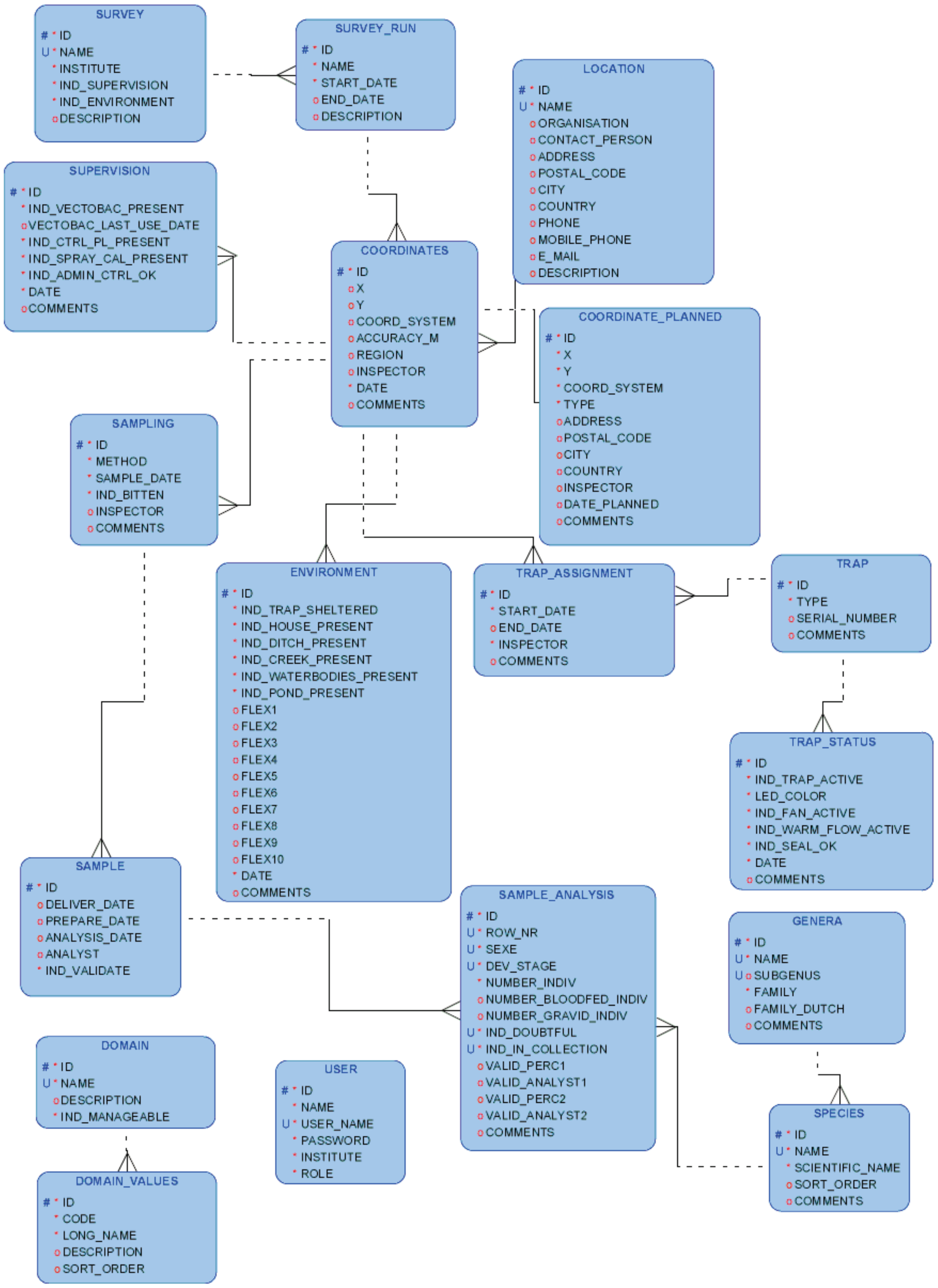

'VecBase' is a vector-surveillance database used in the Netherlands. The tables that are not connected to the main database contain complementary information, i.e. on database users, or drop-down lists for domains. Coordinates planned are those suggested by computerised random selection, while other coordinates are the ones used in the field. IDs are specific to each table.

Source: Dutch National Centre for Monitoring of Vectors (CMV), NVWA

Figure B: Example of web interface

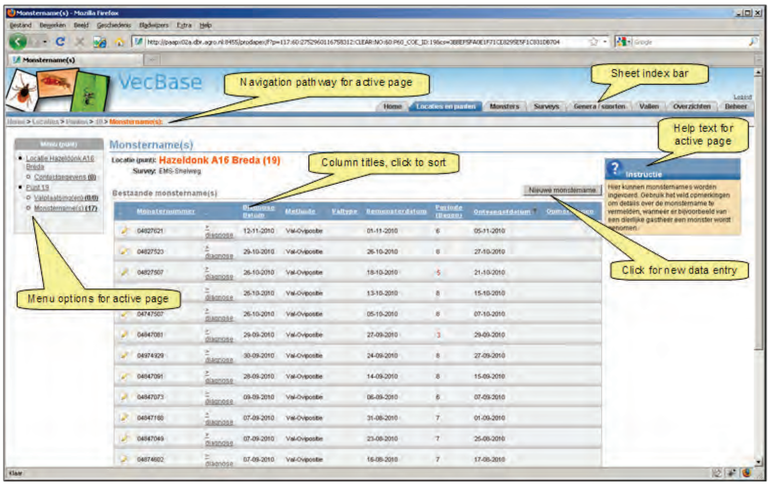

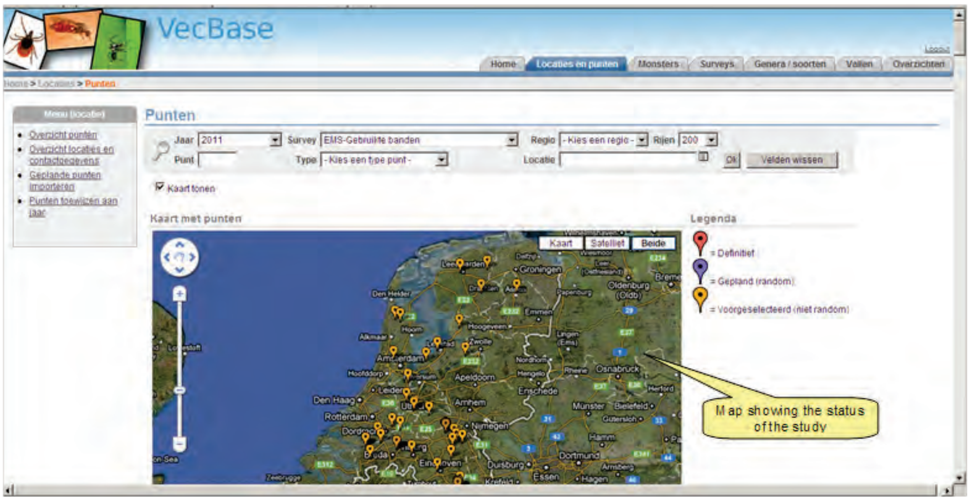

Above: Screenshots of a database user interface specifically designed for vector surveillance ('VecBase', used in the Netherlands)

Source: Dutch National Centre for Monitoring of Vectors (CMV), NVWA

Figure C: Example of process flow within a vector surveillance programme ('VecBase', Netherlands)

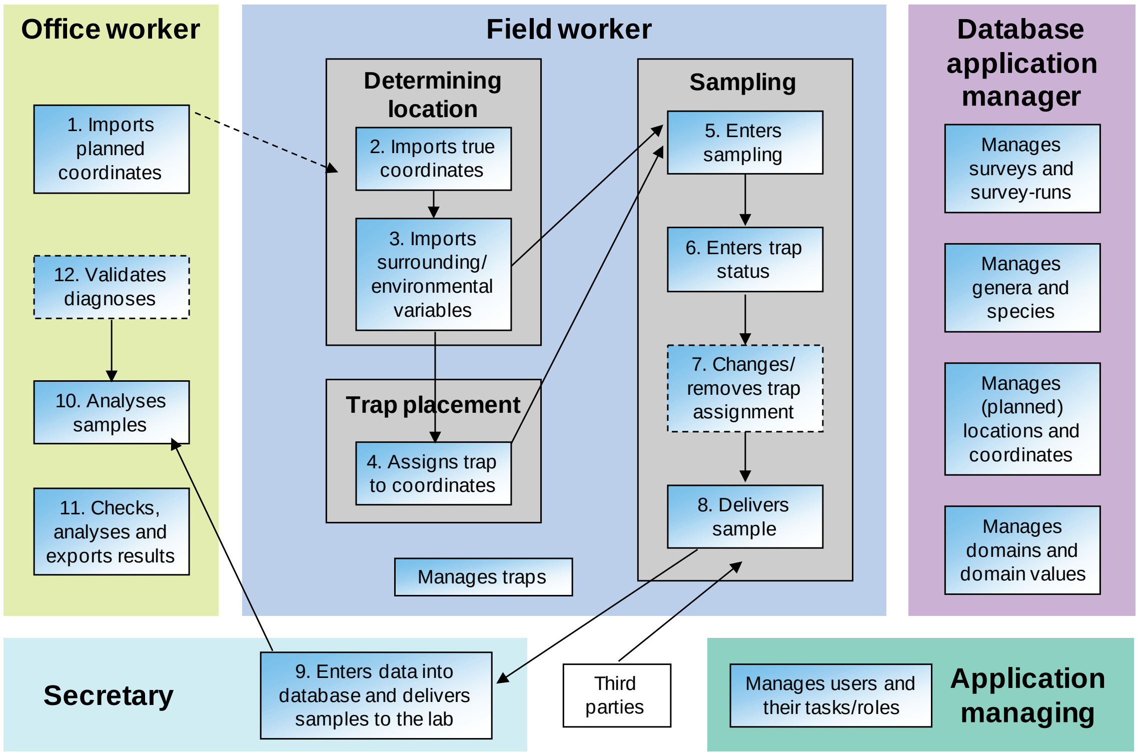

Continuity of data sets

In order to make decisions on elimination/control programmes of IMS, policymaking/legislation regarding IMS, communication to stakeholders, assessment of control effectiveness, and to investigate trends in introduction/establishment/spread of IMS in a defined area, it is crucial to have access to long-term surveillance data for the area of interest. Surveillance activity levels depend on financial, political, institutional, and other factors (change or lack of change in presence/absence/density of IMS, pathogen-transmission, etc). Major objectives, however, should be to prevent loss of data sets (which can happen when surveillance is interrupted and/or changes in partnerships occur) and avoid data gaps, and thus obtain a continuous data flow on all surveillance activities.

Data harmonisation through standardisation

Data management is part of mosquito surveillance, and a well-developed data management system is a prerequisite for efficient data sharing and analysis. Its development requires investments in terms of time, energy, and money, but the data management system will definitely prove its usefulness, especially where large data sets are involved. Data analysis/mapping on a European scale may require data sources that originate from various countries and areas. To avoid countless databases and surveillance organisations that may be difficult or even impossible to compare, it is important to harmonise not only surveillance design, but also data collection management. Only harmonised standards of data collection and data management make it possible to meaningfully compare temporal and spatial data.

Annex 9: Complementary information on methods of dissemination of surveillance results

Table A: **Most suitable dissemination method according to the type of surveillance data and information relevant for dissemination**

Type of data and information to be disseminated		Most suitable dissemination method								
		Report	Newsletter	E-mail	Website	Press release	Local media and TV	Door-to-door leaflet	Scientific publication	Telephone
**Surveillance data results**	Description/text	✓	✓	✓	✓	✓			✓	
	Tables/figures/maps	✓	✓		✓				✓	
	New finding			✓	✓	✓	✓			✓
**Background information**	Why survey for IMS?	✓	✓		✓	✓	✓	✓	✓	
	IMS biology	✓	✓		✓				✓	
	IMS and public health	✓	✓	✓	✓				✓	
	Why control IMS?				✓	✓	✓	✓	✓	
**What can the reader do?**			✓		✓	✓	✓	✓		✓

Table B: **Format of data, efficiency and main audiences according to the type of dissemination method**

Dissemination method	Format of data						Main audiences					
	Text	Figures	Tables	Maps	Speed to reach audience	Comprehensiveness	Policy makers	Researchers	Technicians[1]	Local authorities	Public in affected areas	General public
**Report**	✓	✓	✓	✓	Low	High	✓	✓	✓	✓		
**Newsletter**	✓	✓	✓	✓	Low	Medium	✓		✓	✓	✓	
**Dedicated website**	✓	✓	✓	✓	High	High	✓		✓	✓	✓	✓
**E-mail**	✓				High	Low	✓		✓			
**Press release (national)**	✓				High	Low						✓
**Local media and TV**	✓				High	Low				✓	✓	
**Door-to-door leaflet**	✓	✓		✓	Medium	Medium			✓	✓	✓	
**Scientific publication**	✓	✓	✓	✓	Low	High	✓	✓	✓			
**Telephone**	✓				Very high	Low	✓		✓			

[1] *In charge of control measures or member of the surveillance team*

Methods of dissemination: strengths and weaknesses

Reports

Considered ‘grey literature’, reports are nonetheless very useful for stakeholders, policymakers, mosquito control and public health units, but are largely inaccessible to journalists, scientists and the general public. Reports can hold vast amounts of data, thus providing precise and detailed information.

However, writing a report requires considerable resources and production times are long, as the reported surveillance data usually cover relatively long periods. Moreover, reports are static, cannot be easily corrected, and multiple reports may be needed to cover one single area.

Newsletter

A newsletter is a regularly distributed publication, generally about one main topic that is of interest to its subscribers. Newsletters can be distributed as hard copies, or electronically via email (e-newsletters) and websites. Newsletters include news and upcoming events, as well as contact information for general inquiries. Newsletters on IMS may be distributed by, for example, IMS-affected municipalities, control agencies, surveillance groups and/or public health institutes. One of the advantages of a newsletter is that IMS trends can be identified by screening subsequent issues. Also, since newsletters are distributed to a selected group of people that have at least some interest in IMS-related issues, it is probable that recipients have a certain predisposition to the main messages. Newsletters are usually not specific to IMS-related issues, which may obscure the message. Also, newsletters are static and cannot be easily corrected.

Websites

Surveillance units may develop and launch specific websites that cover surveillance activities, objectives, and results. Websites are flexible, easily updated, link to other relevant websites and literature, can handle virtually unlimited amounts of information, and are accessible to everybody who has access to the internet.
As similar topics are discussed on other websites, which are not always backed by scientific data and research, some readers may get confused.

E-mails

E-mails are useful for rapidly communicating new information or data to a selected audience, mostly people involved in surveillance or stakeholders involved in complementary programmes (mosquito control, disease control, etc.).
As e-mails are not a formal data dissemination method, the accuracy and correctness of the data may seem less to some readers, particularly when compared to other formats (e.g. reports and scientific publications).

Press release

National press releases are a useful medium for informing the press and subsequently the general public. If used properly, press releases will greatly enhance transparency, improve involvement of the general public, and demonstrate the usefulness of the implemented surveillance programme. Moreover, press releases can inspire journalists to publish complimentary or more detailed information.
As always, there is a risk that information is misused or misinterpreted, or that the message is exaggerated, alarming rather than informing the public.

Local media

Local media provide coverage of events in a local context. Usually this includes local newspapers, radio and/or TV stations, which inform the local population on issues regarding a defined and relatively small area (mostly ranging from municipality to region/province level). Communication/dissemination via local media does usually not cover detailed information, but may increase in frequency and level of detail if the situation so requires (e.g. presence of IMS coincides with VBD transmission). Local media often depend on information acquired from involved locals, which may bias the message. Often, IMS issues occur in the summer, a slow time for news, and local journalists may allocate a disproportionate amount of time to IMS events, which may be an advantage, but may also garner unwarranted attention from the local population. To inform the local population on IMS-related issues, avoid misinterpretation of facts, and avoid being accused of withholding information that affects the local population, it is advisable to provide a 'press release' (see above) with contact details for well-informed, media-savvy personnel.

Door-to-door leaflets

Door-to-door leaflets are distributed to people living in an IMS-affected area. This dissemination method is effective in reaching the people that are most likely to be affected. In addition to disseminating IMS surveillance results, door-to-door leaflets are arguably amongst the best methods of involving people in an IMS-affected area in preventive and/or control actions that need public involvement. The strength of this approach is that the accuracy of data and advice are assured, and that a lot of information can be provided indirectly by adding website addresses or other internet links.
A weakness is the relatively high cost (editing, printing, and distribution), and the fact that leaflets may not include the most up-to-date information on IMS dispersal, especially if the IMS is new to the area.

Scientific publications

Scientific information on IMS surveillance data is mostly published in scientific journals or as research monographs in book form. Manuscripts published in peer-reviewed journals are considered validated. Scientific publications are authored and co-authored by experts and researchers, all of whom must agree to their content before publication. Published literature is permanently available (and thus useful for trend analysis), and the analysis of surveillance data is assured.
One drawback is the long process from data collection to analysis, writing, and publication, so that often the most recent information cannot be included. Also, the terminology and language used in scientific publications is very technical and may not be readily understood by the general public. Access to some published manuscripts can be restricted or costly.

Telephone

With the advent of the smartphone, the concept of 'telephone' began to change. Voice-only communication with one person is still useful when sharing selected surveillance data and/or recent developments, or when trying to resolve a specific situation. Also, personal contact by phone is helpful but unfortunately also prone to misunderstandings and misinterpretations, especially in international teams with different native languages. Moreover, no records of shared information are kept, which may be an advantage or a disadvantage.
Modern smartphones provide multimedia capabilities and fast online access. Often, smartphones are used to quickly communicate with office and laboratory personnel: transferred images, for example, can help to assess a complicated situation in the field. It should be noted that smartphones are increasingly used as data communication tools and GPS transmitters to connect to servers in advanced vector-surveillance programmes (e.g. VecMap and MODIRISK).

Box: Access to private property

Access to private property is essential for the efficient control of IMS in breeding containers. Owners usually grant access, but access can also be denied or simply not possible if the owners cannot be reached. In tourist areas, many properties are used only part of the year (vacation homes). Abandoned properties are less numerous but even more problematic in terms of accessibility and mosquito proliferation (they usually offer many breeding choices).

Several European countries have already adopted legislation that forces private owners to allow the work of mosquito abatement or health-unit personnel to be carried out on their properties, e.g. in Germany, where prevention measures are included in legislation. However, this is rarely enforced because of the difficulty and length of the application process. Countries should consider legislation which allows rapid public health intervention methods on abandoned properties.

Surveillance and control personnel may be assisted by an official mandate and corresponding identification documents; local authorities should provide advance information to communities in order to prevent hostility from owners (see also the upcoming EMCA/WHO strategic document).
